# Metallomimetic Chemistry of a Cationic, Geometrically Constrained Phosphine in the Catalytic Hydrodefluorination and Amination of Ar–F Bonds

**DOI:** 10.1021/jacs.2c13318

**Published:** 2023-02-04

**Authors:** Karina Chulsky, Irina Malahov, Deependra Bawari, Roman Dobrovetsky

**Affiliations:** School of Chemistry, Raymond and Beverly Sackler Faculty of Exact Sciences, Tel Aviv University, Tel Aviv 69978, Israel

## Abstract

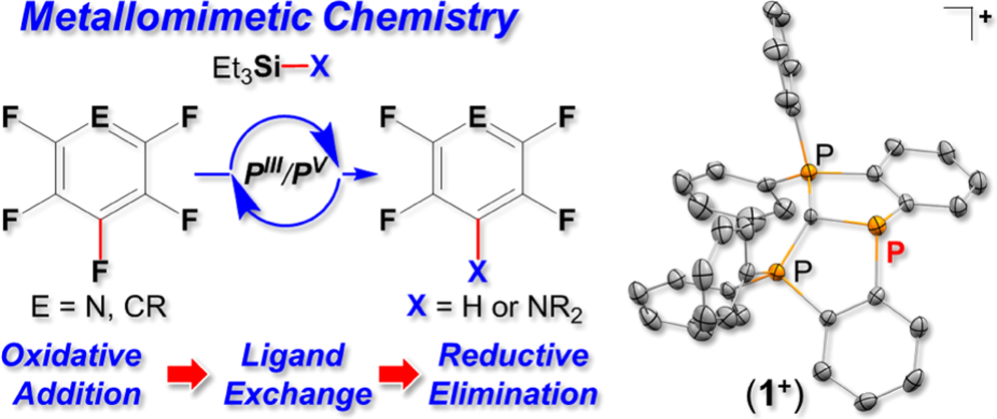

The synthesis, isolation, and reactivity of a cationic, geometrically constrained σ^3^-P compound in the hexaphenyl-carbodiphosphoranyl-based pincer-type ligand (**1**^**+**^) are reported. **1**^**+**^ reacts with electron-poor fluoroarenes via an oxidative addition-type reaction of the C–F bond to the P^III^-center, yielding new fluorophosphorane-type species (P^V^). This reactivity of **1**^**+**^ was used in the catalytic hydrodefluorination of Ar–F bonds with PhSiH_3_, and in a catalytic C–N bond-forming cross-coupling reactions between fluoroarenes and aminosilanes. Importantly, **1**^**+**^ in these catalytic reactions closely mimics the mode of action of the transition metal-based catalysts.

## Introduction

The past decade witnessed a growing interest in the chemistry of geometrically constrained main-group centers and their reactivity.^1^ A lot of work in this field is focused on the chemistry of geometrically constrained P^III^ centers due to their ability to cycle between two stable oxidation states, P^III^ and P^V^, which makes them a potent target for metallomimetic chemistry in catalysis.^2^ In comparison to phosphines with typical trigonal pyramidal geometry that are usually only nucleophilic, geometrically constrained phosphines have an ambiphilic, both nucleophilic and electrophilic, reactivity toward small molecules and often react by insertion of the P^III^ center into strong bonds via an oxidative addition-type reaction.^3^

**Figure 1:**
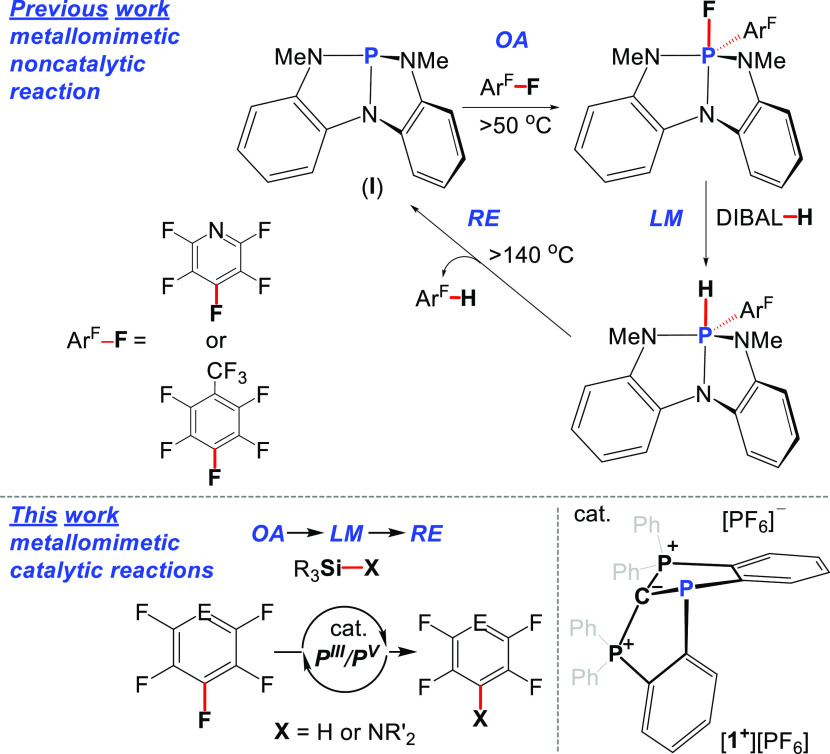
Metallomimetic chemistry of geometrically constrained P^III^ species. Previously reported stepwise, noncatalytic hydrodefluorination of fluoroarenes (top); this work, catalytic hydrodefluorination and amination of fluoroarenes (bottom).

In 1986, Arduengo reported the first *C*_2*v*_ fold, phosphorus center in ONO pincer-type ligand.^4^ Radosevich, in 2012, reported the use of this phosphine to activate ammonia borane and used it to catalytically transfer hydrogen to azobenzene.^5^ Two years later, Radosevich reported a new geometrically constrained P^III^-center with a *C*_*s*_ local symmetry (**I**, Figure 1),^6^ which showed ambiphilic reactivity in small-molecule activation.^7^ Kinjo reported on a diazadiphosphapentalene with a geometrically constrained phosphorus center that activated ammonia by a P-center ligand-assisted process.^8^ Aldridge and Goicoechea reported on a constrained P^III^-center in an ONO pincer-type ligand that showed ambiphilic reactivity toward amines and alcohols.^9^ In 2018, we reported the synthesis of the first geometrically constrained amphiphilic phosphenium cation, which activated water, alcohols, and ammonia, while the activation of ammonia was reversible.^10^ Recently, we also reported on the phosphenium cation in an NNN pincer-type ligand that reacted with O–H and N–H bonds by the P-center ligand-assisted process, and with Si–H bonds by an oxidative addition-type reaction.^11^ In our last work, we reported on the intramolecular oxidative addition-type reactions of polar C–N bonds with a geometrically constrained P^III^-center.^12^

Importantly, despite the progress made in the field of geometrically constrained P^III^ compounds,^2−12^ which led to a number of P^III^/P^V^ catalytic transformations,^13^ their catalytic application in a metallomimetic fashion, i.e., following “oxidative addition” (OA) → “ligand metathesis” (LM) → “reductive elimination” (RE) steps, is scarce.^5,11^ In fact, to the best of our knowledge there are only two recent reports in which metallomimetic cycles of this type were shown.^7b,11^ In 2020, a noncatalytic hydrodefluorination reaction of pentafluoropyridine and octafluorotoluene following the OA → LM → RE steps using geometrically constrained P^III^ triamide species (**I**) was reported (Figure 1).^7b^ In 2022, we reported a metallomimetic catalytic hydrosylilation of benzaldehyde using a geometrically constrained P^III^ cation.^11^ It is important to mention here that much progress has been recently done in the metallomimetic catalysis involving bismuth-based catalysts,^14^ a heavier analogue of phosphorus. A noteworthy example of Bi^I^/Bi^III^ metallomimetic catalysis following the OA → LM → RE steps was recently reported for hydrodefluorination of fluoroarenes.^15^

Continuing with our efforts to synthesize new geometrically constrained P^III^ cations and study their chemistry in small-molecule activation, we report here the synthesis of a cationic, geometrically constrained P^III^ species (**1**^**+**^) in a hexaphenyl-carbodiphosphoranyl-based CCC pincer-type ligand. The reactivity of **1**^**+**^ in activation of electron-poor fluoroarenes by oxidative addition-type reaction of C–F bonds to the P^III^ center and its use as a catalyst in hydrodefluorination and C–N bond-forming cross-coupling reactions is reported (Figure 1). The mechanism of these catalytic reactions was studied both experimentally and by density functional theory (DFT) computations and closely mimics the transition metal-based catalysis.^16^

## Results and Discussion

**Figure 2:**
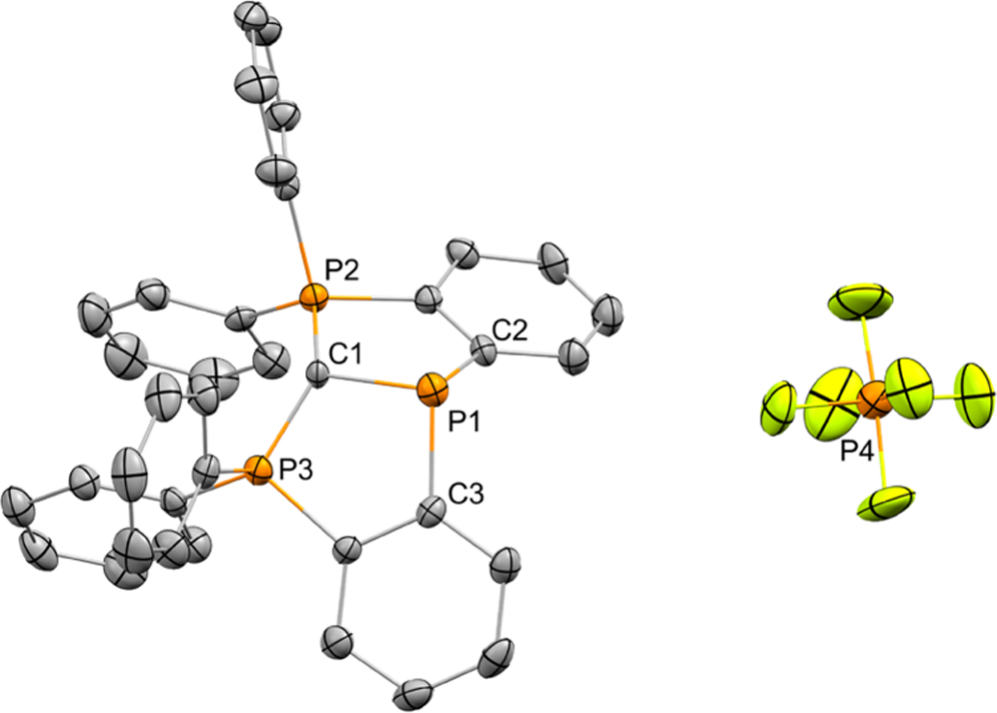
POV-ray depiction of [**1**^**+**^][PF_6_]. Thermal ellipsoids at 30% probability; hydrogen atoms were omitted for clarity.

**Scheme 1:**
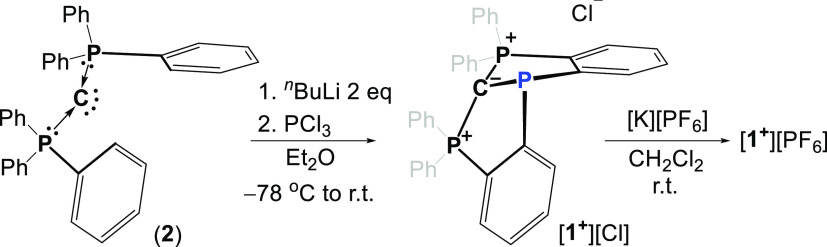
Synthesis of [**1**^**+**^][Cl] and [**1**^**+**^][PF_6_]

**1**^**+**^ was prepared using a similar methodology recently reported by Sundermeyer, which showed that hexaphenyl-carbodiphosphorane can be doubly deprotonated by ^*n*^BuLi, producing a dilithiated hexaphenyl-carbodiphosphorane ligand,^17^ which can be used as a dianionic tridentate CCC pincer-type ligand.^17,18^ Thus, hexaphenyl-carbodiphosphorane (**2**) was treated with 2 equiv of ^*n*^BuLi followed by addition of PCl_3_, which resulted in precipitation of the desired [**1**^**+**^][Cl] (Scheme 1). The ^31^P NMR of [**1**^**+**^][Cl] in CDCl_3_ was measured, showing two broad singlets with chemical shifts of δ = 29.80 and 54.24 ppm with the integration ratio of 2:1, respectively. Thus, the signal at 29.80 ppm was attributed to two phosphorus centers of the carbodiphosphorane unit and the signal at 54.24 ppm to the new P^III^-center. The attempts to crystallize [**1**^**+**^][Cl] failed; however, after Cl^–^ exchange with the weakly coordinating [PF_6_]^−^ anion, and by reaction of [**1**^**+**^][Cl] with [K][PF_6_] (Scheme 1), [**1**^**+**^][PF_6_] was crystallized from a saturated CH_2_Cl_2_/hexane (1:10) solution, and its molecular structure was determined using X-ray crystallography (Figure 2).

Importantly, the ^31^P NMR spectrum of [**1**^**+**^][PF_6_] is nearly identical to that of [**1**^**+**^][Cl] with the exception of a chemical shift at δ = −144.73 ppm appearing as a septet, which corresponds to the [PF_6_]^−^ anion. This means that similarly to [**1**^**+**^][PF_6_], [**1**^**+**^][Cl] is probably a separated ion pair in solution. Noteworthy, in 2018, Uhl reported the synthesis of a neutral geometrically constrained phosphine in CCC pincer-type ligand that exhibited unusual reactivity in coordination chemistry^19^ and synthesis of new heterocyclic molecules.^20^

The geometry around P1 in **1**^**+**^ is significantly distorted from the trigonal pyramidal geometry of the analogous, not geometrically constrained carbodiphosphorane-PPh_2_ adduct [(Ph_3_P)_2_C–PPh_2_]^+^ that adopts a local *C*_3*v*_ symmetry.^21^ The rigid, tridentate carbodiphosphorane-based ligand enforces a strained geometry around the P1 center in **1**^**+**^ with a significant distortion along the P1–C1 axis, resulting in a local *C*_*s*_ symmetry. The two bond angles ∠C1–P1–C3 = 95.66° and ∠C1–P1–C2 = 94.67° in **1**^**+**^ are essentially similar and significantly narrower than those of the previously reported bond angles in [(Ph_3_P)_2_C–PPh_2_]^+^.^21^ The ∠C2–P1–C3 bond angle in **1**^**+**^ of 105.88° is wider than the other two angles and is in the range of previously reported species.^21^ The geometrical distortion of P1 in **1**^**+**^ is however less pronounced than for the previously reported geometrically constrained P^III^ center in the CCC trianionic pincer-type ligand.^19^ Overall, the local geometry around the P1 center in **1**^**+**^ approximates a cis-divacant pseudo trigonal bipyramid in which C2 and C3 atoms of the carbodiphosphorane unit are at the equatorial positions and the central C1 is at the axial one. The P1–C1 bond length of 1.833 Å is typical to P–C single bonds, while P2–C1 and P3–C1 bond lengths of 1.713 and 1.719 Å, respectively, are shorter than a typical P–C single bond.

**Figure 3:**
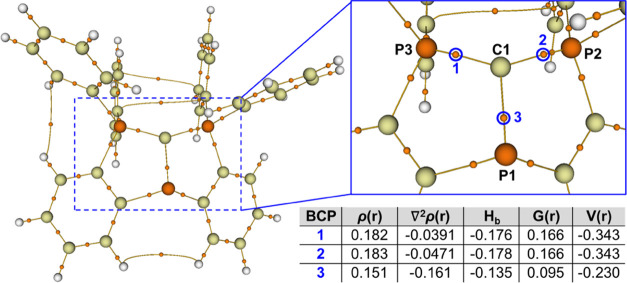
QTAIM analysis of **1**^**+**^.

To get a deeper insight into the structural features of **1**^**+**^, DFT calculations at the BP86-D3/def2TZVP^22^ level of theory were performed. The calculated geometrical parameters of **1**^+^ were in good agreement with the ones obtained from a single-crystal X-ray molecular structure analysis. Natural bond orbital (NBO) analysis revealed the presence of one s-type lone pair (1.86 e^–^ occupancy) at the P1 phosphorus center residing on a sp hybrid (51.00% s and 48.96% p), and a p-type lone pair (1.65 e^–^ occupancy) on the C1 carbon center (97.43% p). The lower electron occupancy at the C1 center is a result of the negative hyperconjugation of its p-type lone pair mostly into the parallel σ*(P(2)/P(3)–C_Ph_) orbitals (0.12965 e^–^ and 0.12445 e^–^ occupancies), which also explains the short P2–C1 and P3–C1 bond lengths. The NBO charges of −1.37962, +0.89314, +1.62167, and +1.62827 on C1, P1, P2, and P3 centers, respectively, were calculated. The Wiberg bond index (WBI) values for C1–P1, C1–P2, and C1–P3 bonds of 0.9089, 1.0450, and 1.0533, respectively, indicate a single-bond nature of these three bonds. The Baders quantum theory of atoms in molecules (QTAIM) was performed to gain insight into the topology of the electron density in **1**^+^ (Figure 3). A negative Laplacian (∇^2^ρ(r)) at the bond critical points (BCP) BCP1 (−0.160802), BCP2 (−0.047073), and BCP3 (−0.039161) (Figure 3) indicates the covalent nature of these bonds, which is also supported by the negative *H*(*r*_c_) (Figure 3). Based on these data, we suggest that the structure of **1**^**+**^ is best described with covalent C1–P1, C1–P2, and C1–P3 single bonds, with +1 formal charges at the P2 and P3 centers, −1 formal charge at the C1 center, and a neutral P1 center.

Molecular orbitals (MO) of the computed **1**^+^ were analyzed. Thus, the highest occupied molecular orbital (HOMO) is localized mostly on P1 and C1 atoms, while the lowest unoccupied molecular orbitals (LUMO) to LUMO + 11 are sets of degenerate orbitals that are mostly localized on the phenyl rings attached to P2 and P3 atoms, which is to be expected, due to the formal positive charge at these two P-centers. The first energetically accessible empty orbital at the central P1 atom is LUMO + 12. The HOMO–LUMO + 12 energy gap in **1**^**+**^ is 3.85 eV, which is 0.85 eV larger than the HOMO–LUMO gap in **I** (Figure 1), computed at the same level of theory (see Supporting Information for more details).

**Scheme 2:**
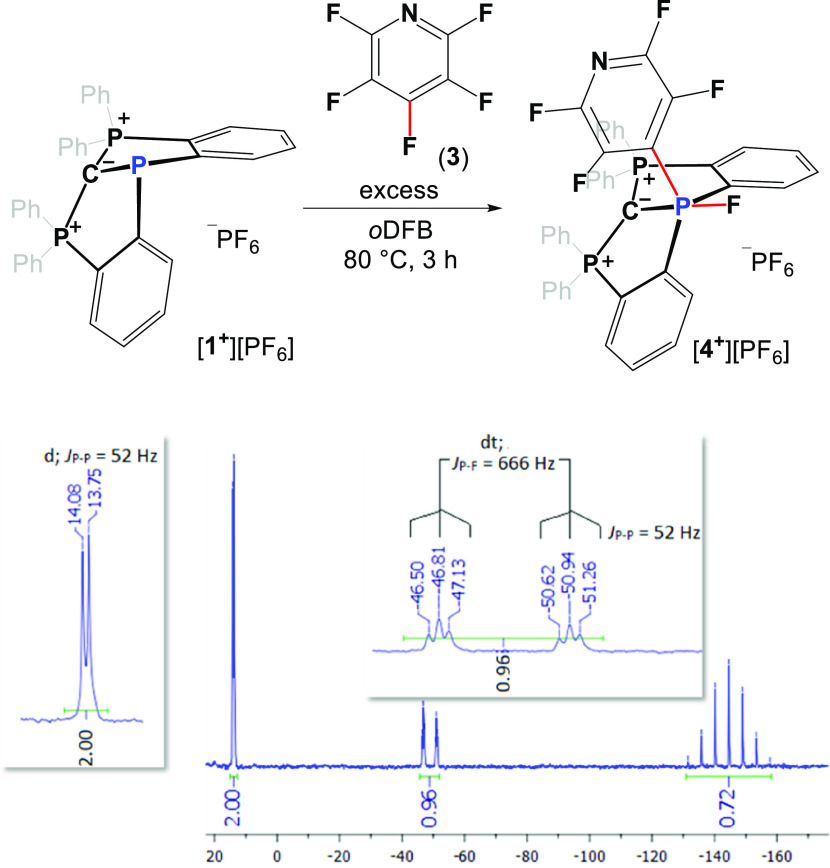
Reaction between [**1**^**+**^][PF_6_] and Excess of **3** Producing the Product of a Formal Oxidative Addition-type Reaction, [**4**^**+**^][PF_6_] The ^31^P NMR spectrum of [**4**^+^][PF_6_] is shown in the inset.

The preliminary reactivity of [**1**^**+**^][PF_6_] with small molecules was tested. While [**1**^**+**^][PF_6_] did not react with Et_2_NH, MeOH, and Et_3_SiH even at elevated temperatures, it did react with electron-poor fluoroarenes. Thus, first [**1**^**+**^][PF_6_] was mixed with excess of pentafluoropyridine (**3**) in 1,2-difluorobenzene (*o*DFB) and heated to 80 °C and the reaction progress was monitored by NMR. After 3 h, two new signals were measured by ^31^P NMR, one as a doublet (^1^*J*(PF) = 666 Hz) of triplets (^2^*J*(PP) = 52 Hz) at δ = −48.87 ppm, and the other as a doublet (^2^*J*(PP) = 52 Hz) at δ = 13.91 ppm (Scheme 2, inset). In ^19^F NMR, a complementary doublet (^1^*J*(PF) = 666 Hz) at δ = 1.92 ppm and two new singlets at δ = −89.32 and −133.71 ppm were measured (Figures S8–S11). Based on the PF and PP coupling in both ^31^P and ^19^F NMR spectra as well as the region of the chemical shifts in ^31^P NMR, we confidently attributed these new signals to the formal oxidative addition product of the C–F bond (at fourth position) in **3** to the P^III^ center in [**1**^**+**^][PF_6_], i.e., compound [**4**^**+**^][PF_6_] (Scheme 2).

Notably, while the oxidative addition-type reaction of Ar–F bonds is not known for typical σ^3^-P compounds, which undergo addition to fluoroarenes by S_N_Ar without forming stable σ^5^-P adducts,^23^ a geometrically constrained **I** was shown to react with Ar–F bonds via an oxidative addition-type reaction, producing stable P^V^ compounds (Figure 1).^7b^ [**4**^**+**^][PF_6_] was then isolated (90% yields) by evaporation of solvents and further characterized by multinuclear NMR and high-resolution mass spectrometry (HRMS). Crystallization of [**4**^**+**^][PF_6_] was attempted from a variety of solvents and at different conditions; however, all of these attempts failed.^24a^

**Scheme 3:**
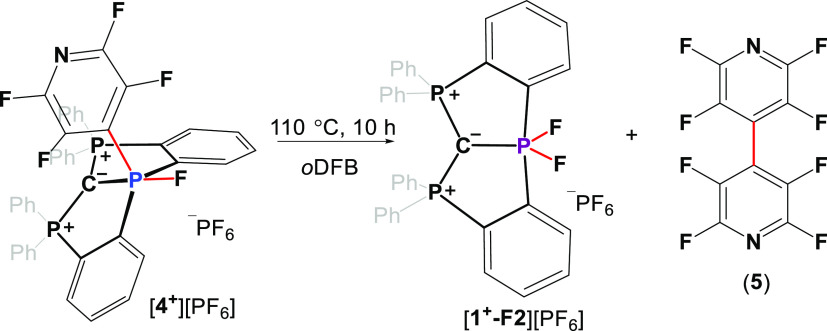
Thermally Induced Reaction of [**4**^**+**^][PF_6_] Producing [**1**^**+**^**-F2**][PF_6_] and **5**

Interestingly, when isolated [**4**^**+**^][PF_6_] dissolved in *o*DFB was heated to 110 °C for 10 h, [**1**^**+**^**-F2**][PF_6_] and perfluoro-4,4′-bipyridine (**5**) were obtained (Scheme 3).^24b^ Although the mechanism for this reaction is not entirely clear, based on the recent report on the heavier Bi analogue,^15^ in which a similar reaction proceeded via disproportionation for series of ligand exchange reactions followed by elimination of **5**,^15^ we assume a similar reaction path occurs in our case.

**Figure 4:**
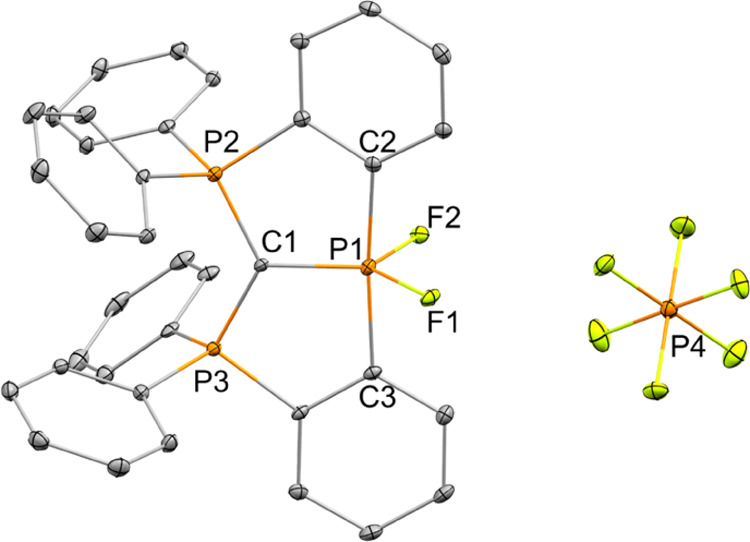
POV-ray depiction of [**1**^**+**^**-F2**][PF_6_]. Thermal ellipsoids at 30% probability; hydrogen atoms were omitted for clarity.

[**1**^**+**^**-F2**][PF_6_] was isolated by crystallization from CH_2_Cl_2_/hexane (1:10). The molecular structure of [**1**^**+**^**-F2**][PF_6_] was determined using X-ray crystallography (Figure 4). The P1 in **1**^**+**^**-F2** has a slightly distorted trigonal bipyramidal geometry with bond angles ∠C1–P1–C2 = 92.50°; ∠C1–P1–C3 = 92.32°; ∠C2–P1–C3 = 173.53°; ∠F1–P1–F2 = 108.57°; ∠C1–P1–F1 = 131.62°; ∠C1–P1–F2 = 119.80°. Interestingly, the rigid carbodiphosphoranyl ligand forces the two F^–^, the most electronegative atoms, to occupy the equatorial positions (Figure 4), which is atypical for difluorophosphoranes in which the fluorides occupy the axial positions.^21,23^ It is important to note here that P^V^ dihalides (with Cl, Br, and I) prepared previously by the reaction of the geometrically constrained P^III^-center in ONO^9b^ or CCC^19^ pincer-type ligand with dihalogens had either square pyramidal geometries with one halogen at basal and other at apical positions for the ONO system,^9b^ or a heavily distorted trigonal bipyramidal geometry with halides (F, Cl, Br) at the equatorial position.^19^

**Scheme 4:**
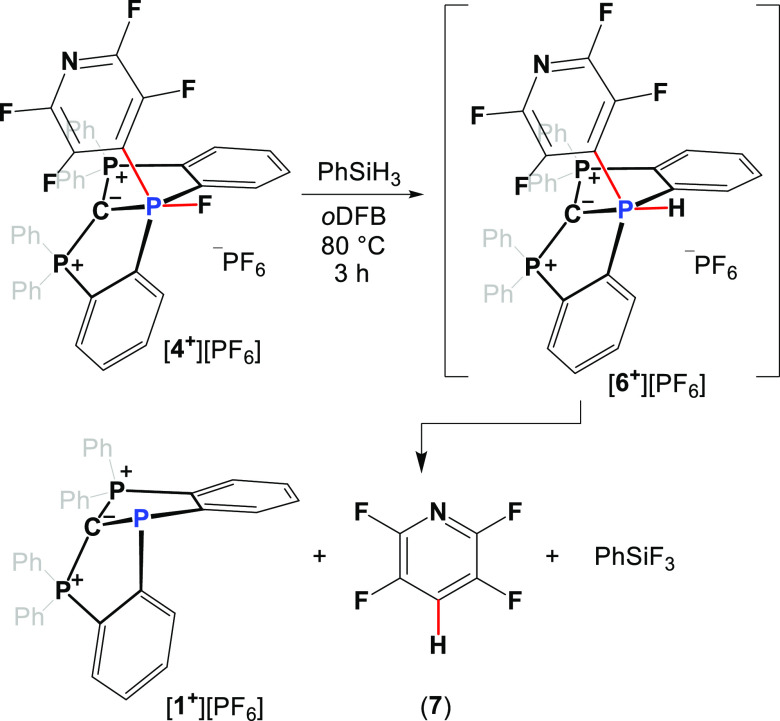
Stoichiometric Reaction between [**4**^**+**^][PF_6_] and PhSiH_3_ Producing the Product of Hydrodefluorination **7**, [**1**^**+**^][PF_6_], and PhSiF_3_ via Intermediate [**6**^**+**^][PF_6_]

We next reacted [**4**^**+**^][PF_6_] with PhSiH_3_ in order to reduce P–F to P–H and obtain hydrophosphorane [**6**^**+**^][PF_6_]. This however led after 3 h at 80 °C directly to [**1**^**+**^][PF_6_], PhSiF_3_, and the product of hydrodefluorination, **7**, while [**6**^**+**^][PF_6_] was not obtained at all.^24c^ This we assumed was a result of a fast reductive elimination-type reaction of the C–H bond from the P-center in the intermediate [**6**^**+**^][PF_6_] (Scheme 4). It is important to note that in a previously reported similar reaction, the product of P–F to P–H exchange was stable and could be isolated in the reaction with DIBAL-H (Figure 1).^7b^ To make sure that the inability to obtain [**6**^**+**^][PF_6_] in our case was not related to the reaction with PhSiH_3_, we performed the reaction of [**4**^**+**^][PF_6_] with DIBAL-H; however, the outcome was similar and [**6**^**+**^][PF_6_] was not obtained (Figure S29).

The inability of [**1**^**+**^][PF_6_] to react with hydrosilanes and at the same time the hydrodefluorination reaction described in Scheme 4 provide an opportunity to use [**1**^**+**^][PF_6_] as the catalyst in the hydrodefluorination reaction of fluoroarenes. Thus, a reaction of **3** with PhSiH_3_ in the presence of a catalytic amount of [**1**^**+**^][PF_6_] (10 mol %) was done in *o*DFB at 80 °C, which after 3 h led to **7** in 95% yield (Table 1). The reaction with pentafluorobenzonitrile with PhSiH_3_ proceeded similarly in the presence of [**1**^**+**^][PF_6_] (10 mol%) and gave after 3 h at 80 °C product **8** (90%) (Table 1). The catalytic hydrodefluorination reaction of methyl pentafluorobenzoate using PhSiH_3_ and [**1**^**+**^][PF_6_] as the catalyst (10 mol %) led to product **9** after 16 h at 120 °C (Table 1). Remarkably, the pyridine in **7**, the nitrile group in **8**, and ester group in **9** all remained intact in these hydrodefluorination reactions, meaning that this method is tolerant toward these functional groups. The [**1**^**+**^][PF_6_]-catalyzed hydrodefluorination reactions of octafluorotoluene and decafluorobiphenyl with PhSiH_3_ leading to **10** and **11**, respectively, proceeded much slower (9 and 19 h, respectively) and required a higher temperature of 120 °C (Table 1). Notably, the reaction of tris(pentafluorophenyl)phosphine with PhSiH_3_ in the presence of [**1**^**+**^][PF_6_] (10 mol %) led after 48 h at 120 °C to product **12**, in which all of the fluorides at the para-position were substituted by hydrides (Table 1).

**Table 1:**
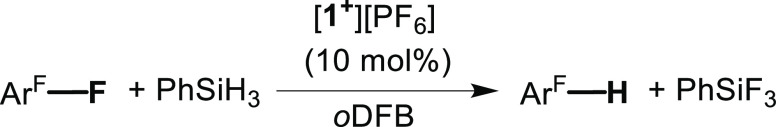

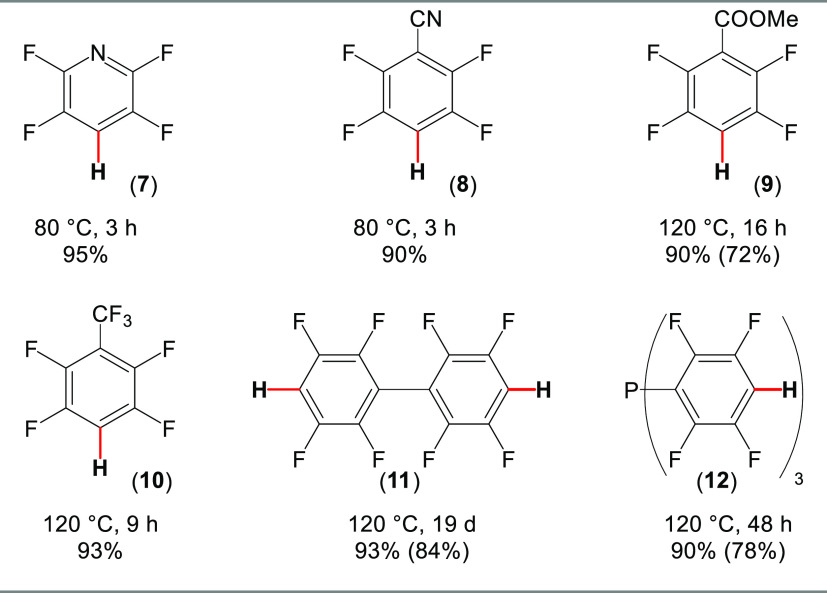
Hydrodefluorination of Fluoroarenes Using PhSiH_3_^24c^ Catalyzed by [**1**^**+**^][PF_6_]^a^,^b^

a Isolated yields in parentheses.

b The reactions of some of the substrates with PhSiH_3_ were attempted without the presence of [**1**^+^][PF_6_] and did not produce any reactivity even after prolonged heating.

Interestingly, the selectivity of [**1**^**+**^][PF_6_] in the catalytic hydrodefluorination reactions shown above is completely different from that of a previously reported dicationic P^III^ species, which catalyzed the hydrodefluorination of alkyl fluorides only via the Lewis acidic path,^25^ pointing to a different mechanism of these hydrodefluorination reactions. Although, as was previously mentioned, the oxidative addition-type reaction of the electron-poor Ar–F to P^III^ centers was reported (Figure 1),^7b^ the catalytic hydrodefluorination reaction of fluoroarenes in a metallomimetic P^III^/P^V^ redox cycle has not yet been shown to the best of our knowledge. While the reason for the inability to perform catalysis with the previously mentioned **I** (Figure 1) was not specifically mentioned,^7b^ it is likely that **I** reacts with DIBAL-H leading to the deactivation of the catalyst; this assumption is supported by the fact that **I** reacts with B–H bonds via the P-center/ligand-assisted path.^7a^ As mentioned previously, however, the metallomimetic catalytic hydrodefluorination reaction of fluoroarenes using the Bi^I^-based catalyst with a lower catalyst loading and a larger scope, milder conditions, and shorter reaction times (compared to the catalysis using [**1**^**+**^][PF_6_]) was recently achieved.^15^

Next, we were interested in applying the reactivity of **1**^**+**^ with Ar^F^–F bonds in the catalytic C–N bond-forming cross-coupling reactions. Thus, we first reacted **3** with Et_3_Si–NEt_2_ (**13**) in the presence of 10 mol% of [**1**^**+**^][PF_6_] in *o*DFB, which led after 2.5 h at 80 °C to the product of C/N cross-coupling (**14**) in 92% yield (Table 2). A similar result was obtained for the catalytic reaction of pentafluorobenzonitrile with **13**, which after 2.5 h led to complete conversion to the product of C/N cross-coupling (**15**) (Table 2). Methyl pentafluorobenzoate reacted with **13** and the catalytic amount of [**1**^**+**^][PF_6_] (10 mol %) very slowly, leading after 15 days to a mixture of two C/N cross-coupling products at ortho and para positions (**16** and **17**, respectively) in 1:4 ratio (Table 2). The cross-coupling reaction between **13** and octafluorotoluene catalyzed by [**1**^**+**^][PF_6_] gave after 23 h at 120 °C **18** in 93% yield. Decafluorobiphenyl reacted extremely slowly with **13** in the presence of [**1**^**+**^][PF_6_], producing **19** (90%) after 25 days at 120 °C (Table 2). The catalytic C–N bond-forming cross-coupling reaction of the primary silylamine, Et_3_Si–N(H)Bn (**13a**), with **3** and pentafluorobenzonitrile catalyzed by [**1**^**+**^][PF_6_] (10 mol%) was performed as well, leading to **14a** (87%) and **15a** (95%), respectively, after 16–22 h at 80 °C (Table 2). [**1**^**+**^][PF_6_] (10 mol%)-catalyzed amination of tris(pentafluorophenyl)phosphine using **13** (1 equiv) led to product **20** after 55 h at 120 °C (Table 2). It is important to note here that the transition metal-free catalytic amination of fluoroarenes using the magnesium-based catalyst was recently reported; however, the mechanism for this amination reaction proceeded through an S_N_Ar-type reaction.^26^

**Table 2:**
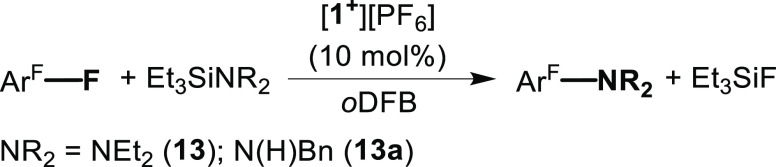

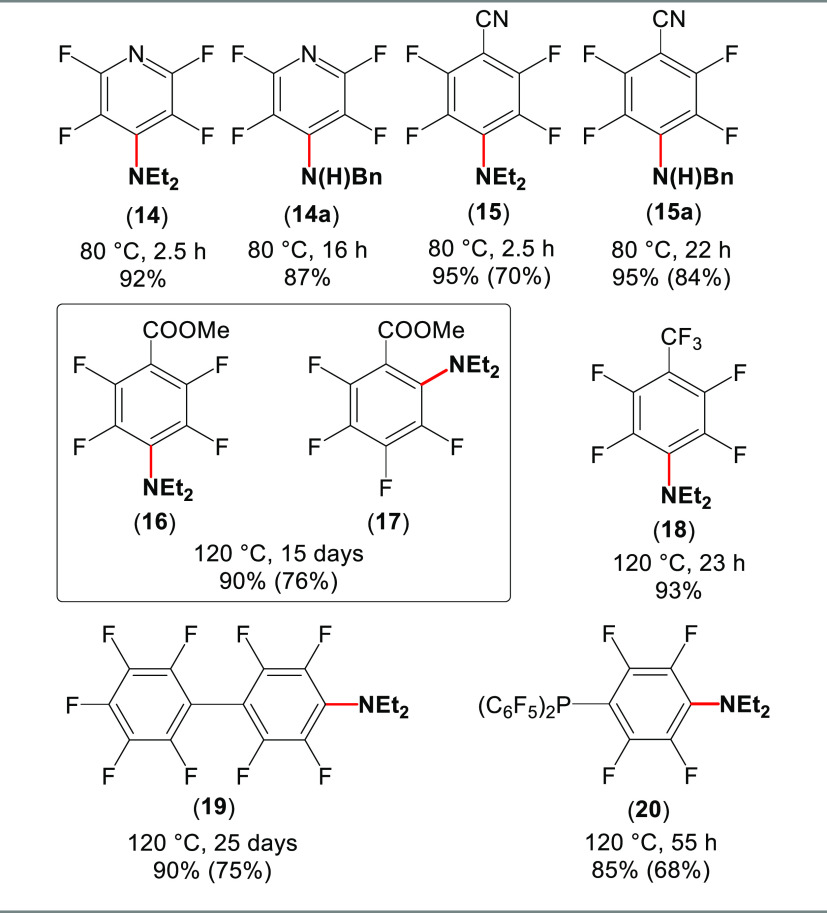
[**1**^**+**^][PF_6_]-Catalyzed Amination of Fluoroarenes^a^,^b^

a Isolated yields in parentheses.

b The reactions of some of the substrates with Et_3_SiNEt_2_ were attempted without the presence of [**1**^**+**^][PF_6_], and did produce products in a very low conversion ratio (below 10%) after much longer heating.

**Scheme 5:**
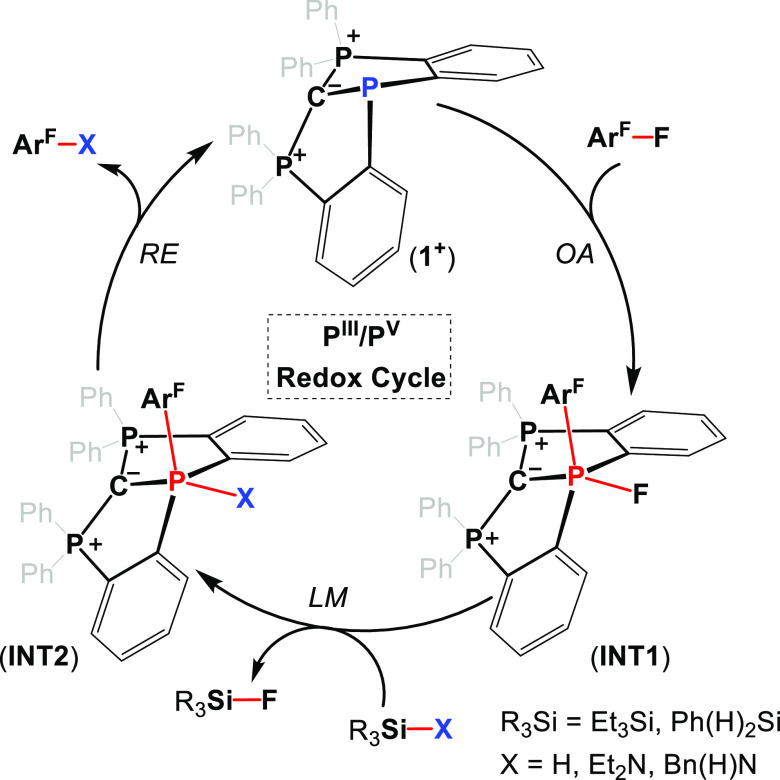
Hydrodefluorination and C–N Bond-Forming Cross-Coupling Reactions Catalyzed by **1**^**+**^

We believe that both catalytic processes, hydrodefluorination and C–N bond-forming cross-coupling, proceed via similar steps in which **1**^**+**^ mimics the transition metal catalyst’s behavior. Thus, the first step is the oxidative addition-type reaction (OA) of the C–F bond to the geometrically constrained P^III^ center in **1**^**+**^, giving a stable intermediate (**INT1**) (Scheme 5). The next step is the ligand metathesis (LM)-type reaction, in which the fluoride at the P-center is replaced by either a hydride or an amino group leading to the intermediate (**INT2**) (Scheme 5). The last step of this catalytic cycle is the reductive elimination-type reaction (RE) of C–H or C–N bonds from the P-center in **INT2** producing the products of the hydrodefluorination or the C–N bond-forming cross-coupling and regenerating the catalyst (**1**^**+**^) (Scheme 5).

**Figure 5:**
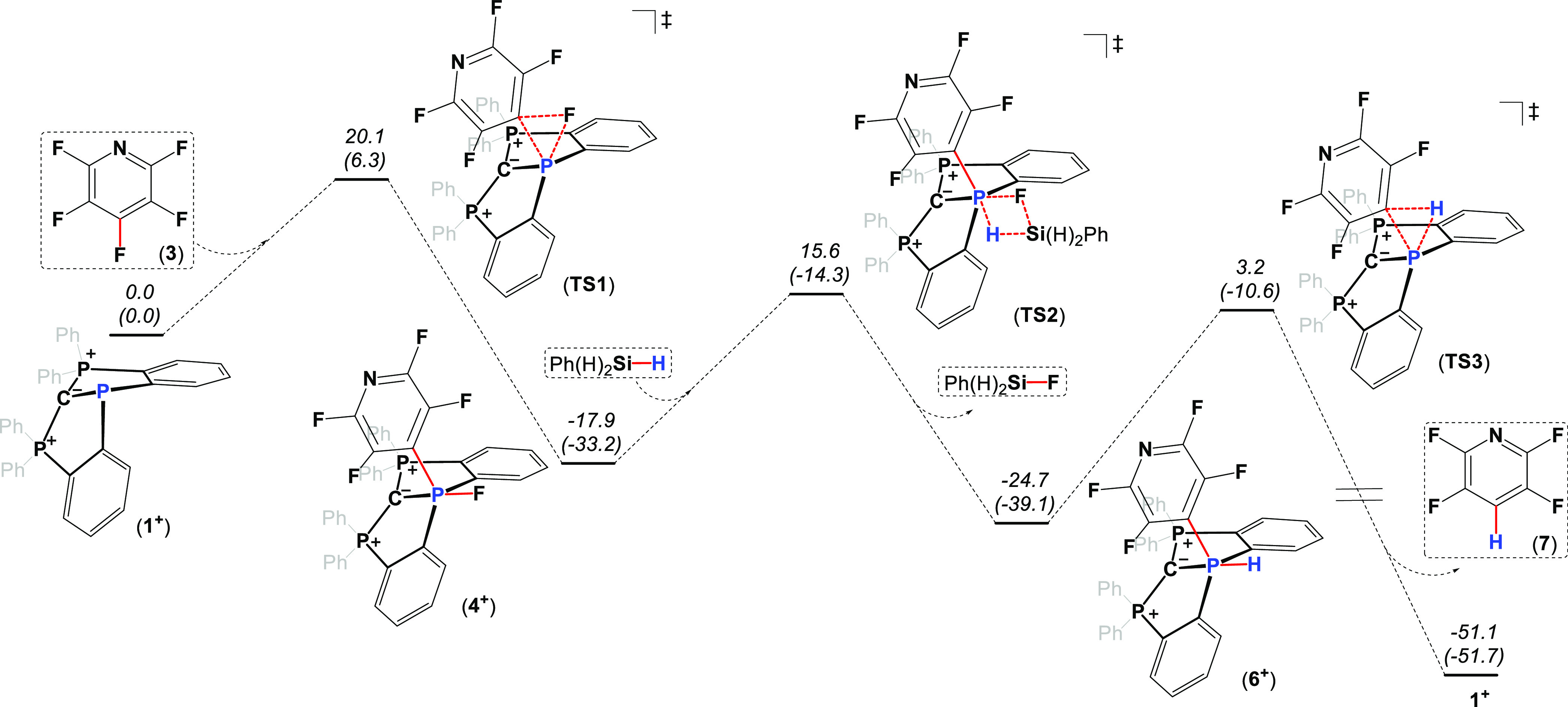
DFT-calculated (BP86-D3/def2TZVP)^22^ potential energy surface of the proposed mechanism of **1**^**+**^-catalyzed hydrodefluorination of **3** by PhSiH_3_. Free Gibbs energies (enthalpies) are given relative to the starting materials.

To study further the mechanism of these catalytic reactions, DFT computations of the potential energy surface (PES) for the hydrodefluorination reaction of pentafluoropyridine (**3**) using PhSiH_3_ catalyzed by **1**^**+**^ were performed at the BP86-D3/def2TZVP level theory.^22^ As a result, the first step of the reaction, which is the oxidative addition-type reaction of the C–F bond to the P^III^ center leading to **4**^**+**^, is exergonic (Δ*G* = −17.9 kcal·mol^–1^) and strongly exothermic (Δ*H* = −33.2 kcal·mol^–1^), which proceeds through the free Gibbs energy barrier of Δ*G*^‡^= 20.1 kcal·mol^–1^ (**TS1**) (Figure 5). The next step, a ligand metathesis process, presumably producing **6**^**+**^, is again exergonic and exothermic with ΔG = −6.8 and Δ*H* = −5.9 kcal·mol^–1^ and proceeds via the transition state **TS2** with Δ*G*^‡^ = 33.5 kcal·mol^–1^ (Figure 5). Based on the calculated geometry of **TS2**, the ligand exchange is a concerted σ-bond metathesis process. Importantly, the ligand metathesis (**4**^**+**^ to **6**^**+**^) is a rate-determining step, which explains the inability to observe **6**^**+**^ in the reaction. The last step of the reaction, which is the reductive elimination-type reaction of the C–H bond from the P^V^ center in **6**^**+**^ leading to **7** and **1**^**+**^, is strongly exergonic and exothermic (Δ*G* = −26.4 and Δ*H* = −12.6 kcal·mol^–1^) and proceeds via transition state **TS2** with Δ*G*^‡^ = 27.9 kcal·mol^–1^ (Figure 5). This mechanistic picture is also supported by the variable temperature (VT) NMR experiment, in which only [**1**^**+**^][PF_6_] was measured by ^31^P-NMR during the catalysis, meaning that it is indeed the resting state of this catalytic cycle (Figure S28). We believe that the reaction with aminosilanes proceeds via similar mechanistic steps (Figure S61 for DFT calculation).

## Conclusions

To conclude, a new cationic, geometrically constrained P^III^ species supported by a carbodiphosphorane-based pincer-type ligand [**1**^**+**^][PF_6_] was synthesized. [**1**^**+**^][PF_6_] reacted with electron-poor fluoroarenes via oxidative addition-type reaction of the C–F bonds to the central P^III^ center. This reactivity of [**1**^**+**^][PF_6_] was used for catalytic hydrodefluorination and the C–N bond, forming cross-coupling reactions. The mechanism of these two catalytic processes was investigated both experimentally and computationally and proceeds in a metallomimetic fashion by following the OA → LM → RE steps. We continue to study the chemistry of the geometrically constrained P centers and their potential in catalysis.

## Supporting Information Available

NMR; experimental; and computational details (PDF)
