# Supplementary material for: Metallomimetic Chemistry of a Cationic, Geometrically Constrained Phosphine in the Catalytic Hydrodefluorination and Amination of Ar–F Bonds
Source: J Am Chem Soc. 2023 Feb 4;145(6):3786–94. doi: 10.1021/jacs.2c13318 (PMC9936586; doi:10.1021/jacs.2c13318)
Supplement: Supplementary file 1 — ja2c13318_si_001.pdf [file ja2c13318_si_001.pdf]

Metallomimetic Chemistry of a Cationic, Geometrically Constrained Phosphine in the Catalytic Hydrodefluorination and Amination of Ar−F Bonds

Karina Chulsky, Irina Malahov, Deependra Bawari and Roman Dobrovetsky*

School of Chemistry, Raymond and Beverly Sackler Faculty of Exact Sciences, Tel Aviv University, Tel Aviv 69978, Israel

Table of Contents

1. General experimental considerations..S2

2. X-ray Crystallography S2

2. Synthetic Procedures..S2

4. DFT Computations...S41

5. References..S68

1. General experimental considerations:

All preparations were carried out under an anhydrous N_2 atmosphere using standard Schlenk and glove box techniques. All glassware was oven dried and cooled under vacuum before use. Commercial reagents were purchased from Sigma Aldrich, Strem or Apollo Scientific and used without further purification unless indicated otherwise. **2** was prepared following the reported procedure.[1] NMR spectra were recorded at room temperature using a Bruker AvanceIII-400 MHz spectrometer. Data for ^{1}H NMR are reported as follows: chemical shift (*δ* ppm), integration, multiplicity (s = singlet, d = doublet, t = triplet, q = quartet, quin = quintet, sep = septet, m = multiplet, br = broad), coupling constant (Hz), assignment.

2. X-ray Crystallography:

Single crystal X-ray diffraction data were collected on a Bruker KAPPA APEX II diffractometer equipped with an APEX II CCD detector using a TRIUMPH monochromator with a MoK*α* X-ray source (*α* = 0.710 73 Å). The crystals were mounted on a cryoloop with Paratone oil, and all data were collected at 100(2) K. Unit cell determination and refinement and data collection were done using the Bruker APEX-II suite,[2] data reduction and integration were performed using SAINT v8.34A (Bruker, 2013)[3] and absorption corrections and scaling were done using SADABS-2014/5 (Bruker,2014/5)[4]. All the crystal structures were solved through OLEX2[5] package using SHELXT[6] and the structures were refined using SHELXL[6]. All non-hydrogen atoms were refined anisotropically. All the figures were generated using Mercury 3.10.2.

3. Synthetic Procedures:

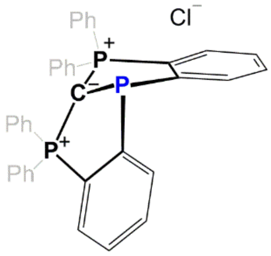

Synthesis of [1⁺][Cl]: Dilithiated hexaphenyl-carbodiphosphorane (**2-Li₂**) ligand was prepared in Et_2O (40 mL) solution from **2** (610 mg, 1.14 mmol) using a reported procedure.[7] PCl_3 (1 mL, 1.14 mmol) was added at -78 ^{o}C. The reaction mixture was allowed to warm to room temperature and stirred for overnight. A pale-white powder precipitated from the colorless solution. The Et_2O solution was decanted and the remaining solid was dried under vacuum and washed with hexane (50 mL). The solid obtained was dissolved in DCM and filtered to remove LiCl. All the volatiles were evaporated under vacuum to afford a yellow-creamy powder. Yield: 520 mg (76.11%). **^{1}H-NMR** (500 MHz, $CDCl_3$), *δ*: 6.91 (m, 4H), 7.13 (t, *J* = 8 Hz, 4H), 7.41

(t, *J* = 8 Hz, 2H), 7.64-7.65 (br-m, 8H), 7.68 (m, 2H), 7.74 (t, *J* = 8 Hz, 2H), 7.97 (m, *J* = 8 Hz, 4H), 8.14 (t, 8 Hz, 2H). **^{13}C-NMR** (125.8 MHz, $CDCl_3$), δ: 120.57 (d, *J* = 72 Hz), 128.72, 129.94, 130.22, 130.76, 131.75, 132.75, 133.50, 133.77, 134.23, 151.27 (br) ppm. **^{31}P-NMR** (202.5 MHz, $CDCl_3$): δ = 29.80 (s, C*P*$_2$), 54.24 (s, C*P*) ppm. HRMS (ES+): Calculated for $C_{37}H_{28}P_3$: 565.1404 [M-Cl]$^+$; Obs: 565.1417.

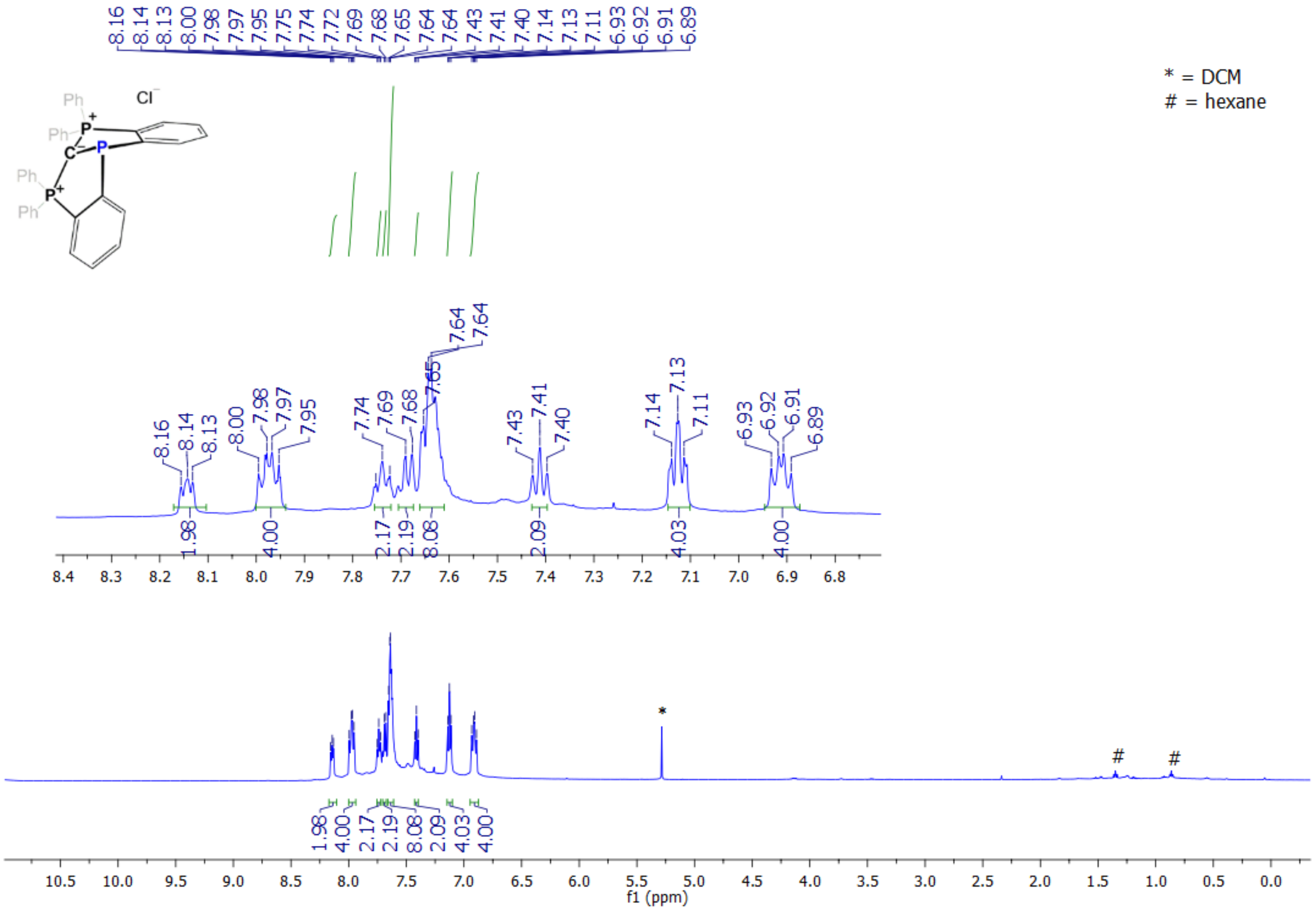

Figure S1. ^{1}H NMR spectrum (500 MHz, in $CDCl_3$) of [**1**$^+$][Cl].

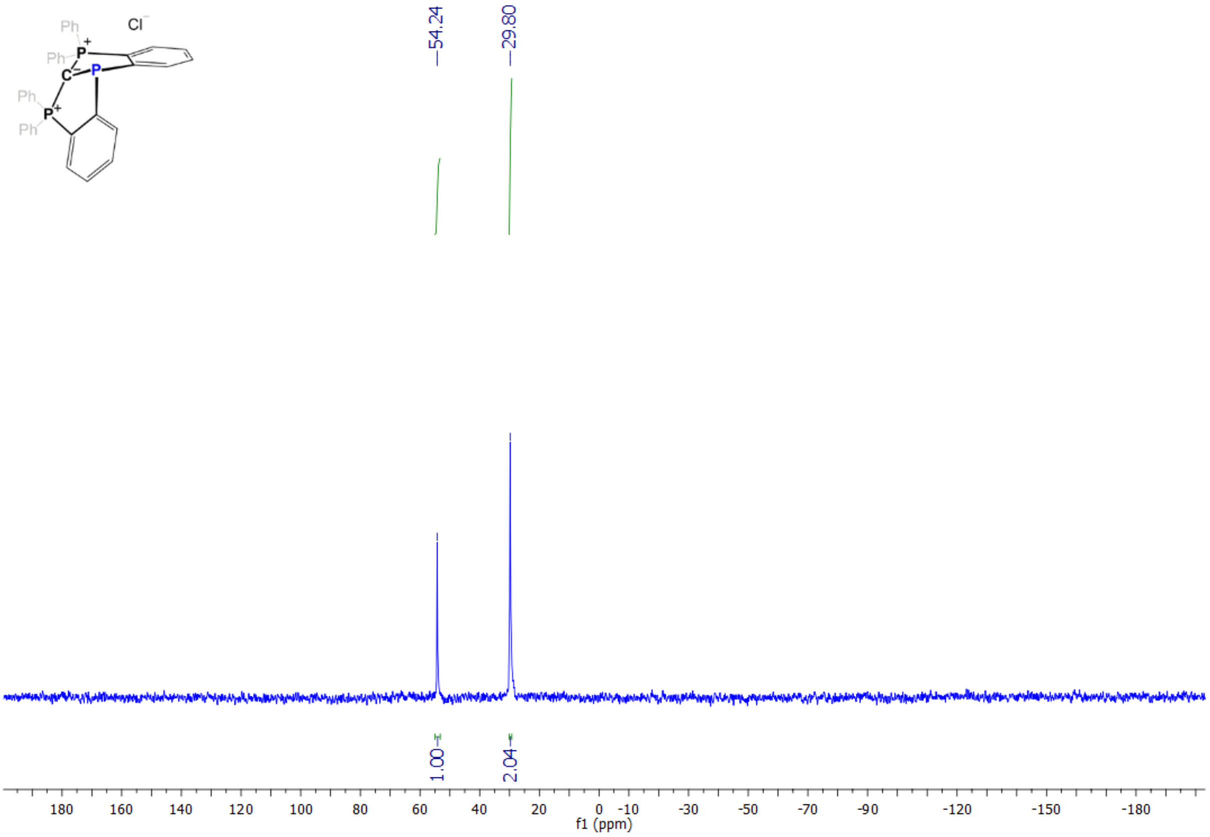

Figure S2. ^{31}P NMR spectrum (202.5 MHz, in $CDCl_3$) of [**1**$^+$][Cl].

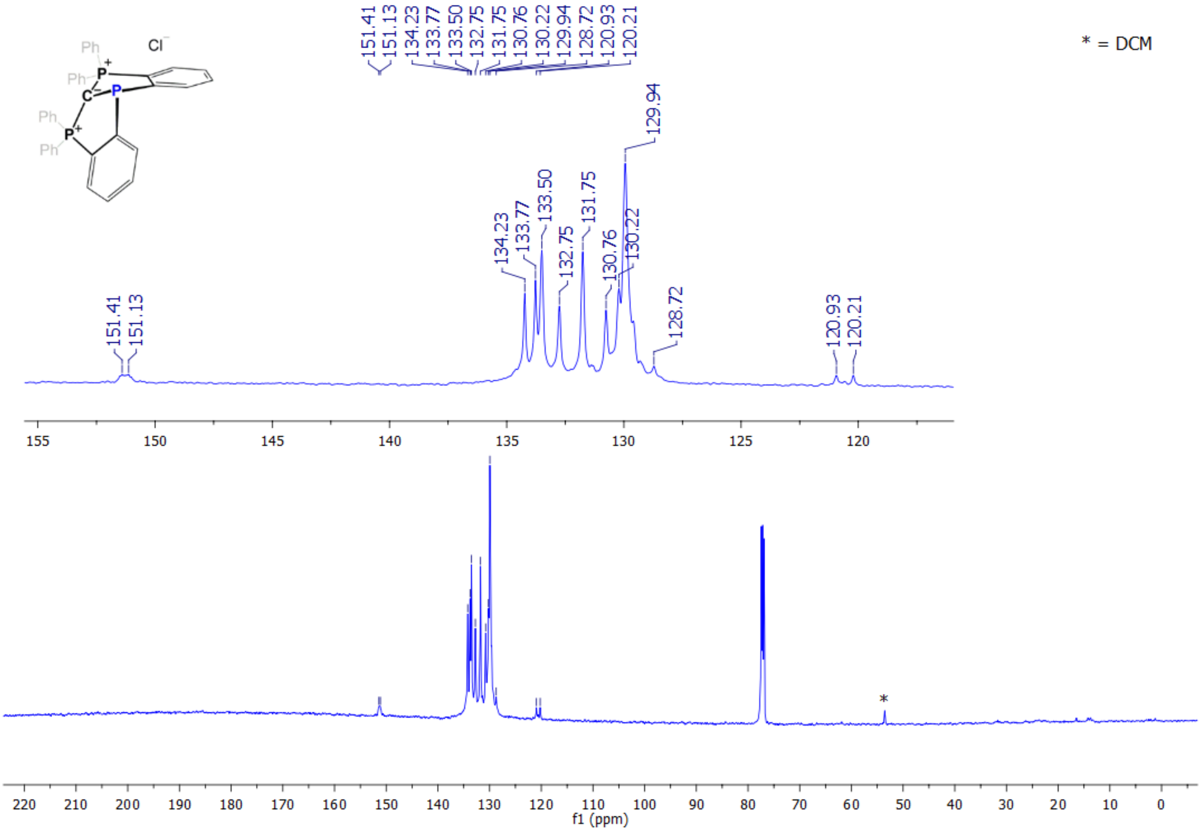

Figure S3. ^{13}C NMR spectrum (125.8 MHz, in $CDCl_3$) of [**1**$^+$][Cl].

Synthesis of [1⁺][PF₆]: KPF_6 (175.2 mg, 0.952 mmol) was added to a 10 mL DCM solution of [**1**⁺][Cl] (520 mg, 0.865 mmol) and the mixture was stirred overnight at room temperature. The solution was filtered to separate KCl salt. All the volatiles were evaporated under vacuum to produce a yellow-beige powder. Dissolving this powder in 1:10 DCM/hexane mixture and slow evaporation of this solution at room temperature after 2 days afforded yellow crystals. Yield: 605 mg (98.41%). **^{1}H-NMR** (400 MHz, $CDCl_3$), *δ*: 6.82 (m, 4H), 7.00 (t, *J* = 8 Hz, 4H), 7.30 (t, *J* = 8 Hz, 2H), 7.51-7.63 (m, 12H), 7.91 (m, 4H), 8.05 (t, *J* = 8 Hz, 2H). **^{13}C-NMR** (100 MHz, C_6D_6), *δ*: 120.55 (d, *J* = 90 Hz), 129.85, 130.24, 130.66, 131.74, 132.66, 133.41, 133.66, 134.16, 151.37. **^{31}P-NMR** (162 MHz, $CDCl_3$), *δ*: -144.73 (sep, *J* = 711 Hz, PF_6), 31.41 (s, CP_2), 55.70 (s, *CP*) ppm. **^{19}F-NMR** (376.5 MHz, $CDCl_3$), *δ*: -73.85 (d, 711 Hz) ppm. **HRMS** (ES+): Calculated for $C_{37}H_{28}P_3$: 565.1404 $[M-PF_6]^+$; Obs: 565.1417.

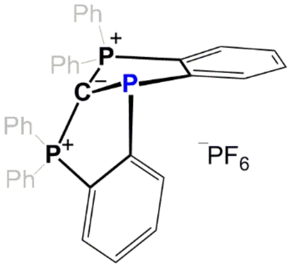

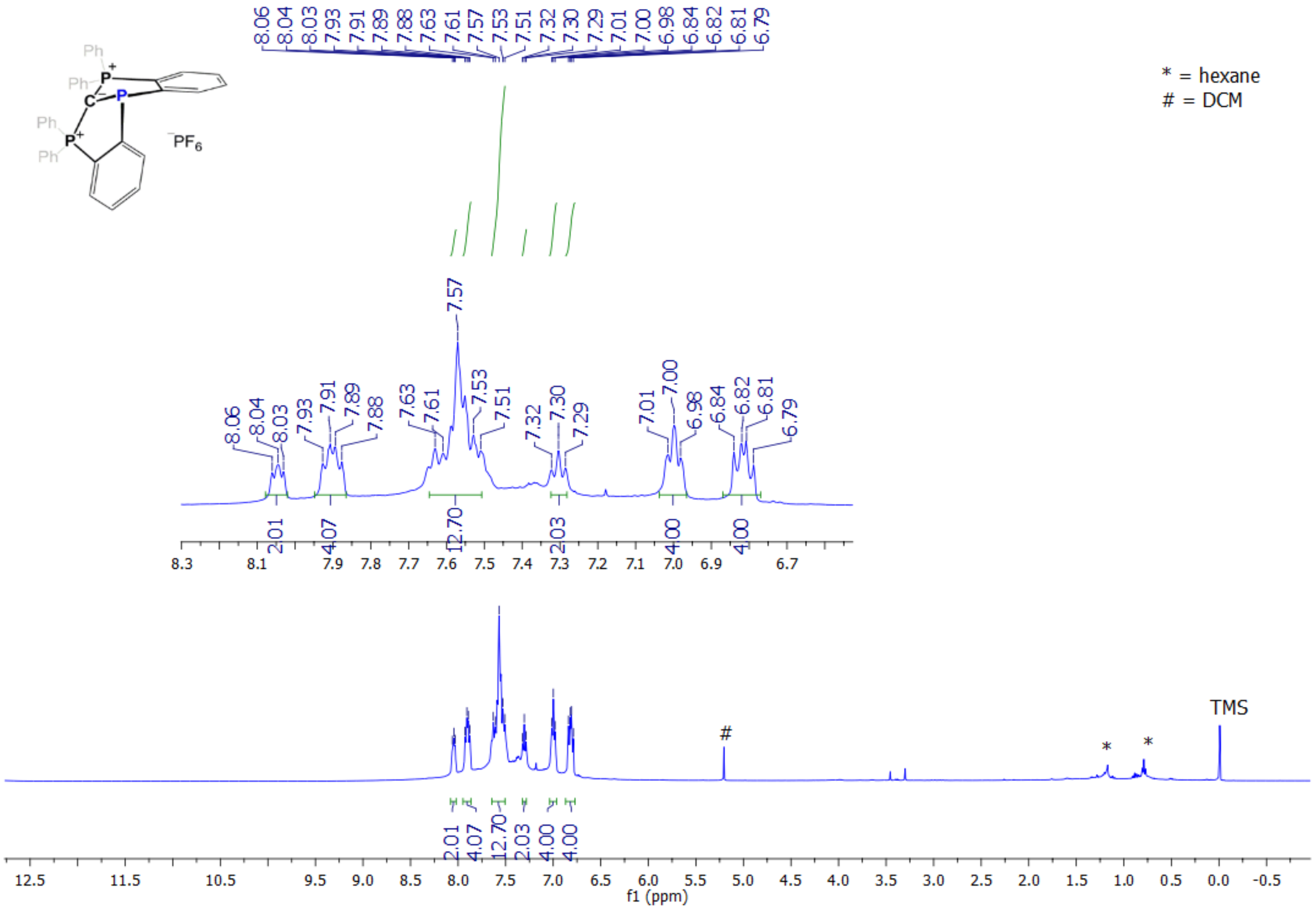

Figure S4. ^{1}H NMR spectrum (400 MHz, in $CDCl_3$) of [**1**⁺][PF_6].

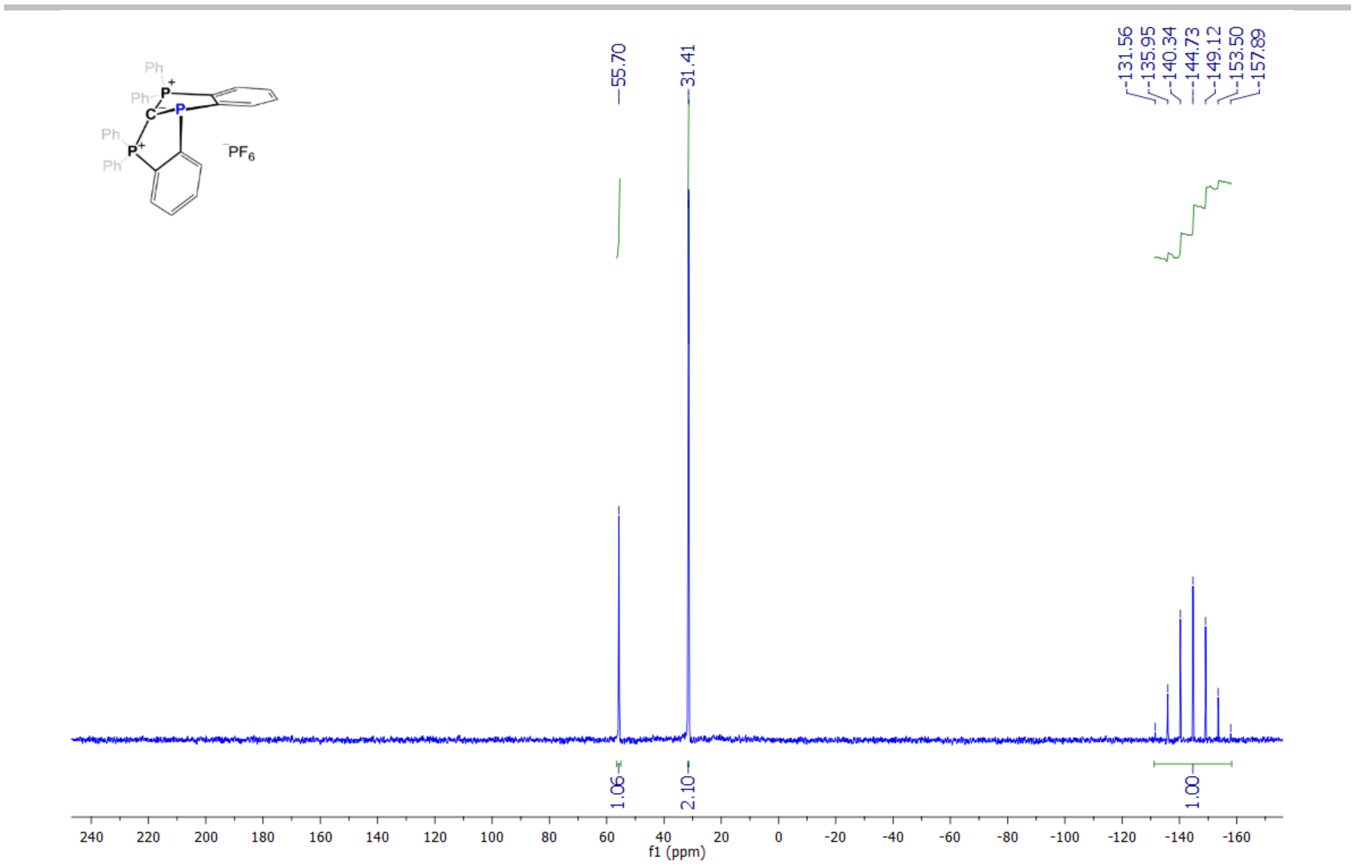

Figure S5. ^{31}P NMR spectrum (162 MHz, in $CDCl_3$) of [**1**$^{+}$][PF_6].

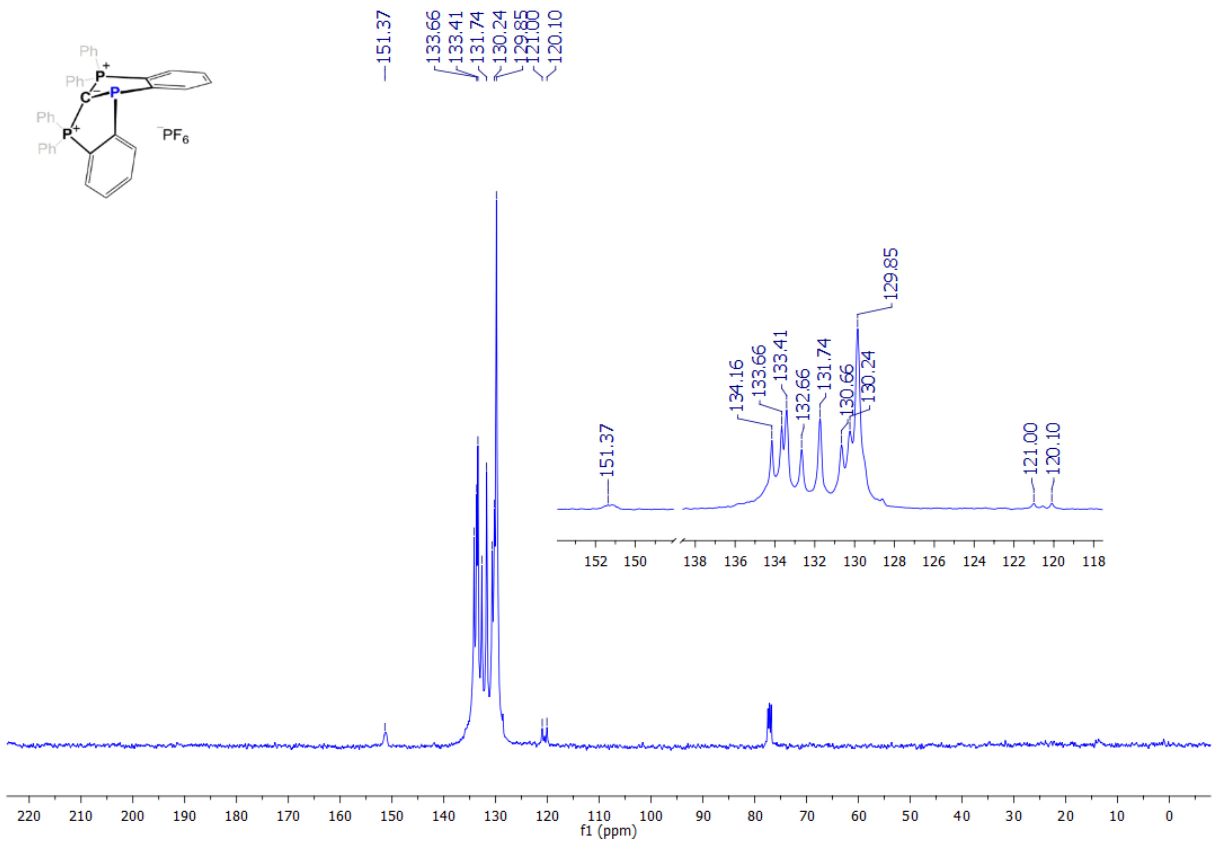

Figure S6. ^{13}C NMR spectrum (100 MHz, in $CDCl_3$) of [**1**$^{+}$][PF_6].

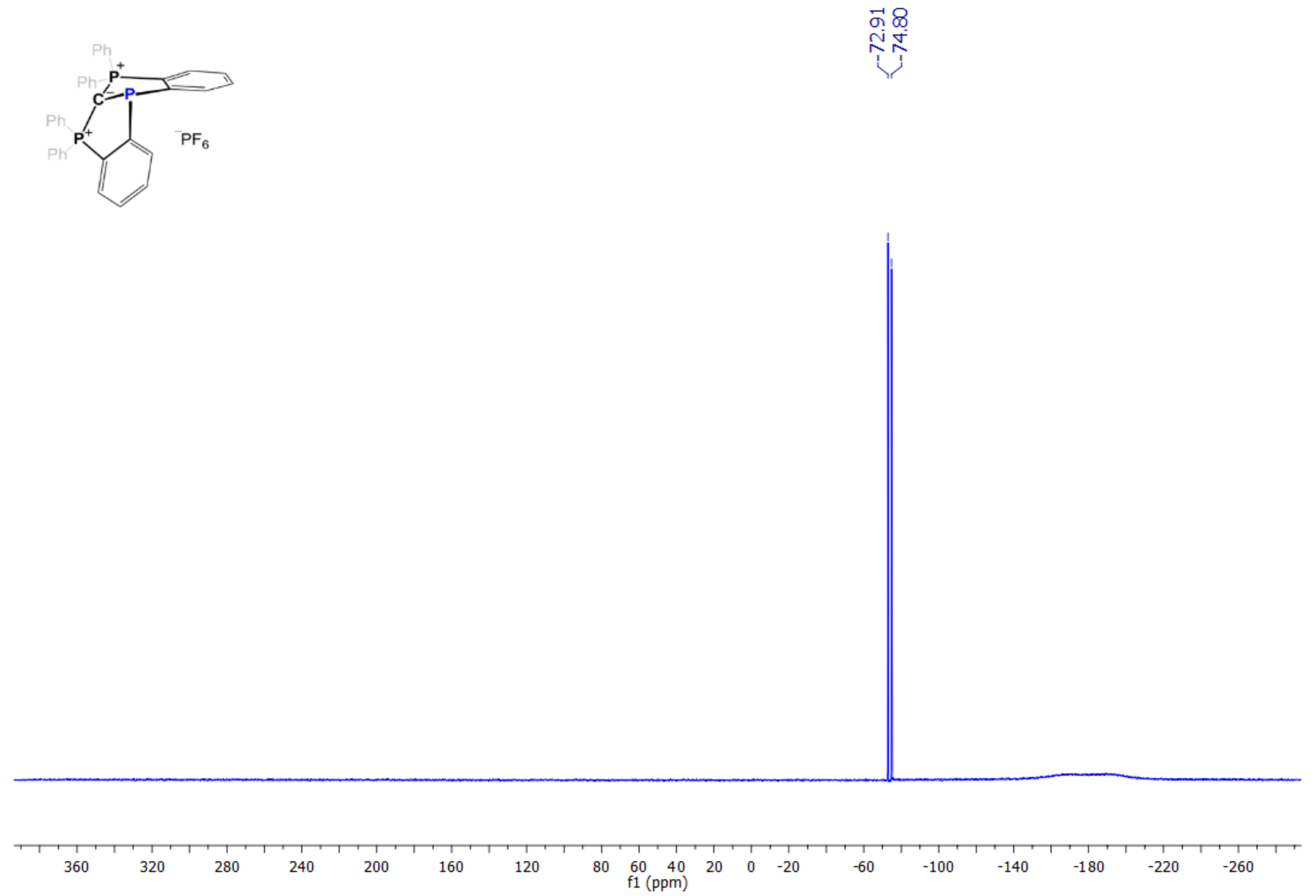

Figure S7. ^{19}F NMR spectrum (376.5 MHz, in $CDCl_3$) of [**1**$^+$][PF_6].

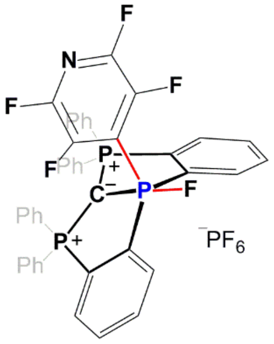

Synthesis of [4$^+$][PF$_6$]: 10 equivalents of pentafluoropyridine (232 µL, 2.11 mmol) were added to a J-Young NMR tube containing 0.5 mL *o*-DFB solution of [**1**$^+$][PF_6] (150 mg, 0.211 mmol). The reaction progress was monitored by NMR spectroscopy and left for heating at 80 ^{o}C for 3 hours until its full conversion to **4**$^+$. All the volatiles were evaporated under vacuum that yielded product [**4**$^+$][PF_6] (90% NMR yield).

***(4**) is contaminated with a minute amount of oxidized (P=O) product of [**1**$^+$][PF_6]. **^{1}H-NMR** (400 MHz, $CDCl_3$), *δ*: 6.73 (m, 4H), 7.00 (t, *J* = 8 Hz, 4H), 7.27 (t, *J* = 8 Hz, 2H), 7.47 (m, 6H), 7.62 (m, 2H), 7.77-7.91 (m, 8H), 8.74 (m, 2H) ppm. **^{13}C-NMR** (100 MHz, $CDCl_3$), *δ*: 128.46, 129.79, 131.94, 132.59, 133.38, 134.01, 139.16 ppm. **^{31}P-NMR** (162 MHz, $CDCl_3$), *δ*: -144.57 (sep, *J* = 711 Hz, P*F*$_6$), -48.87 (dt, *J* = 666 Hz, 52 Hz, *P*-F), 13.91 (d, *J* = 52, C*P*$_2$) ppm. **^{19}F-NMR** (376.5 MHz, $CDCl_3$), *δ*: -133.71 (s, Ar-*F*), -89.32 (s, Ar-*F*), -73.50 (d, *J* = 715 Hz, P*F*$_6$), 1.92 (d, *J* = 666 Hz, P-*F*) ppm. **HRMS** (ES+): Calculated for $C_{42}H_{28}NF_5P_3$: 734.1355 [M-PF_6]$^+$; Obs: 734.1357.

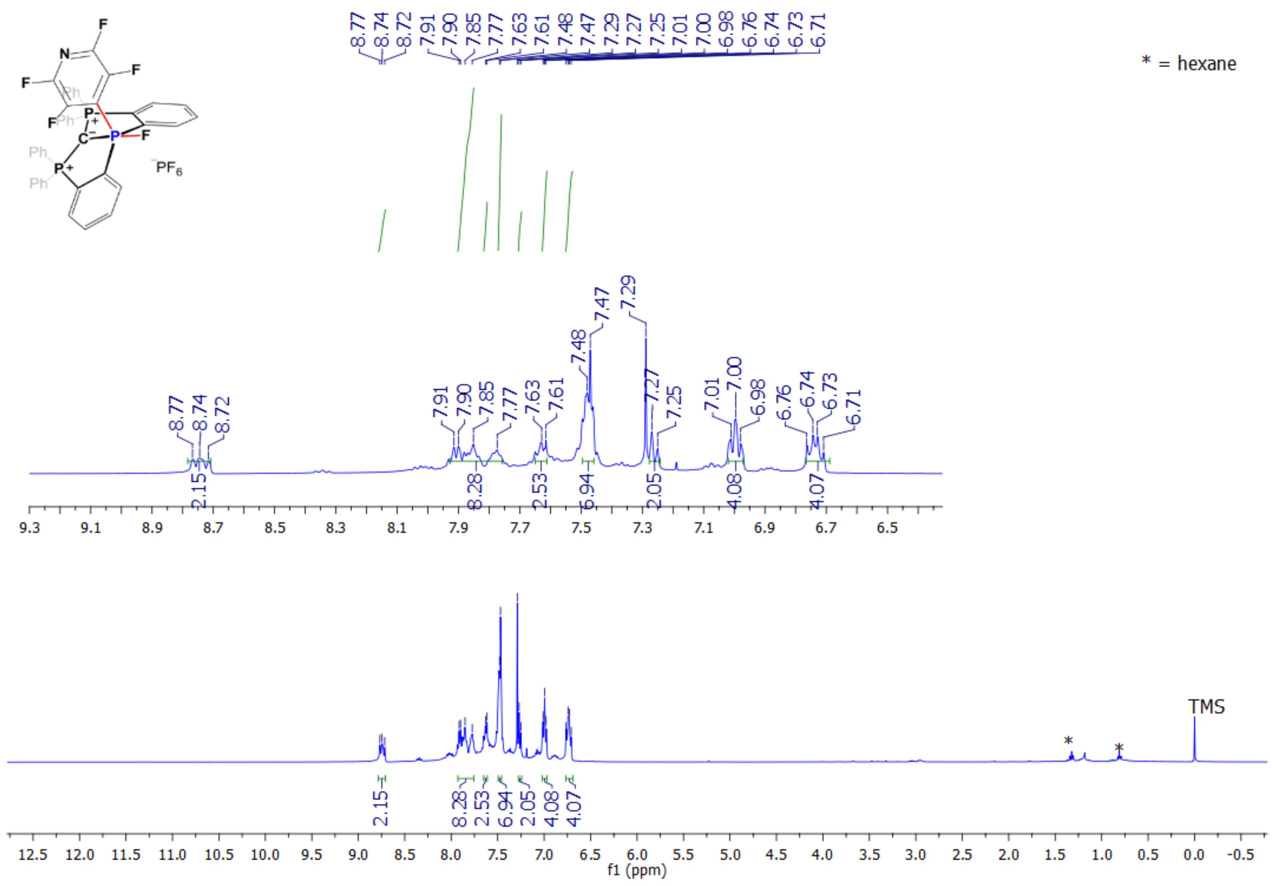

Figure S8. ^{1}H NMR spectrum (400 MHz, in $CDCl_3$) of [**4**$^{+}$][PF_6].

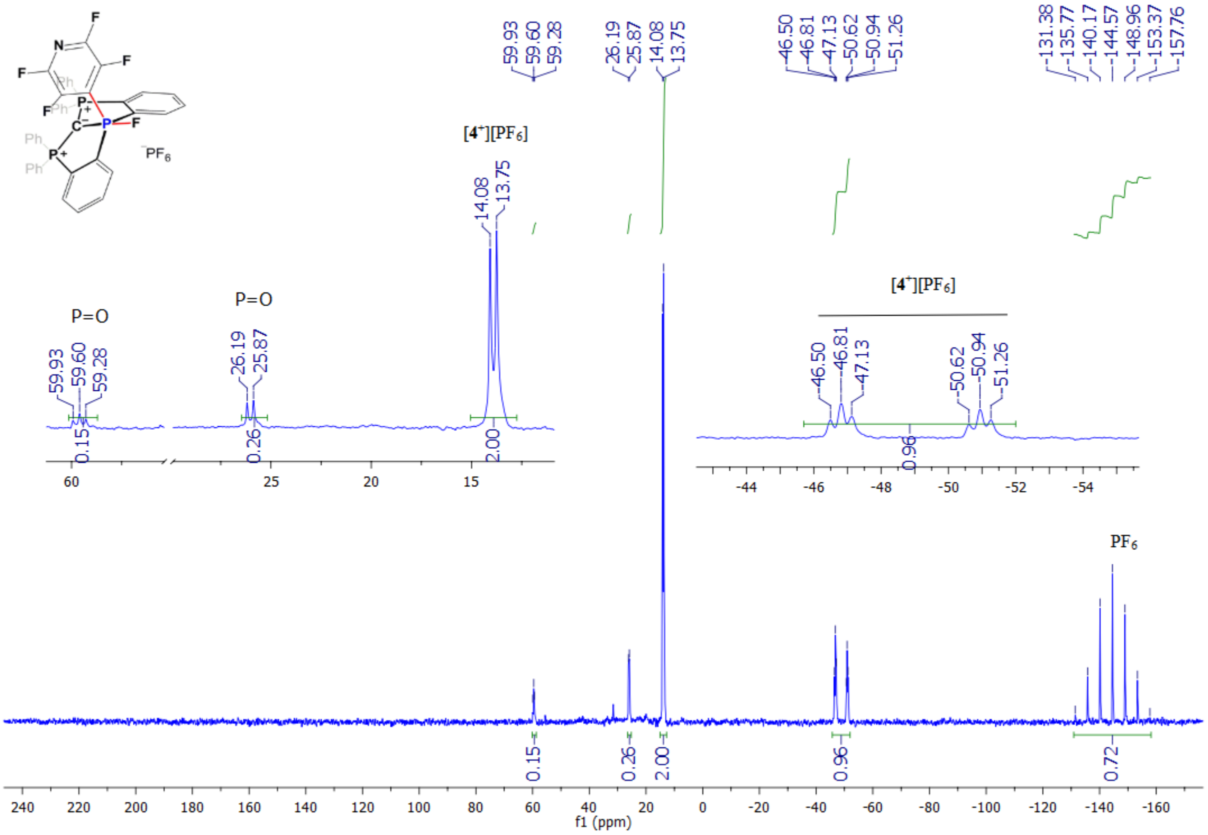

Figure S9. ^{31}P NMR spectrum (162 MHz, in $CDCl_3$) of [**4**$^{+}$][PF_6].

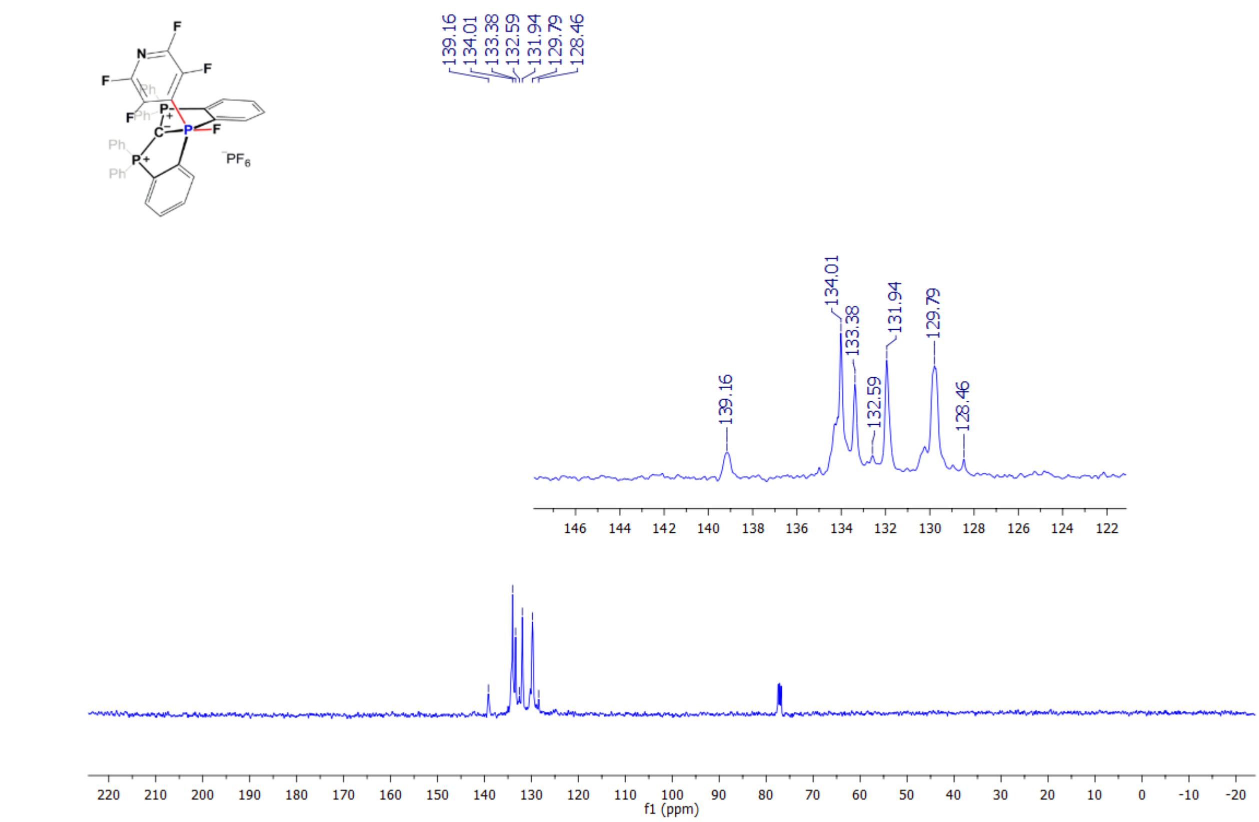

Figure S10. ^{13}C NMR spectrum (100 MHz, in $CDCl_3$) of [**4**$^+$][PF_6].

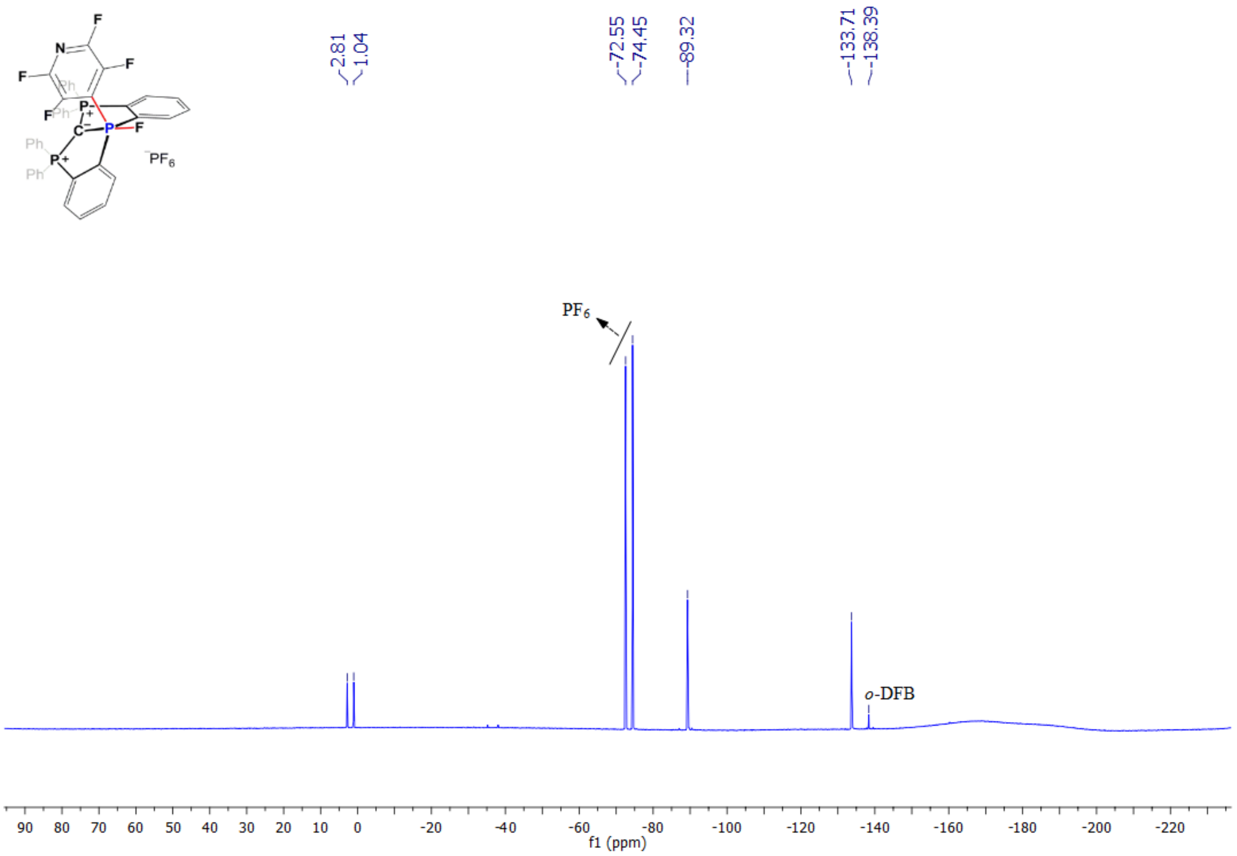

Figure S11. ^{19}F NMR spectrum (376.5 MHz, in $CDCl_3$) of [**4**$^+$][PF_6].

Synthesis of [1^+-F_2][PF_6] and [1^+-O][PF_6]: A J-Young NMR tube was charged with oDFB (0.5 mL) solution of [**4^+**][PF_6] (152 mg, 0.173 mmol). The solution was heated at 110 °C and monitored by NMR spectroscopy. After 10 hours the reaction solution was filtered, evaporated, and fully dried. Crystals of [**1^+-F_2**][PF_6] (Yield: 32 mg (25.07%)) were obtained from by slow evapoartion from DCM/hexane (1:10) solution at room temperature.

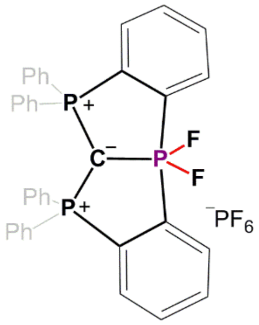

NMR data of [1^+-F_2][PF_6]: **^{1}H-NMR** (400 MHz, in DCM, DMSO-d_6 cap.), *δ*: 7.72-7.81 (m, 16H), 7.97-8.04 (m, 8H), 8.27 (m, 2H), 8.59 (m, 2H). **^{13}C-NMR** (100 MHz, in DCM, DMSO-d_6 cap.), *δ*: 119.80 (t, J = 44 Hz), 127.39 (d, J = 17 Hz), 128.81 (t, J = 6 Hz), 129.94 (br), 131.56 (t, J = 5 Hz), 133.02 (d, J = 12 Hz), 133.28 ppm. **^{31}P-NMR** (162 MHz, in DCM, DMSO-d_6 cap.), *δ* = -145.11 (sep, J = 711 Hz, *P*F_6), -2.08 (tt, J = 1067 Hz, 76 Hz, *P*F_2), 13.74 (d, J = 76 Hz, C*P*$_2$) ppm. **^{19}F-NMR** (376.5 MHz, in DCM, DMSO-d_6 cap.), *δ*: -74.03 (d, J = 711 Hz, P*F*$_6$), -37.41 (d, J = 1069 Hz, P*F*$_2$) ppm. **HRMS** (ES+): Calculated for $C_{37}H_{28}F_2P_3$: 603.1372 [M-PF_6]$^+$; Obs: 603.1386.

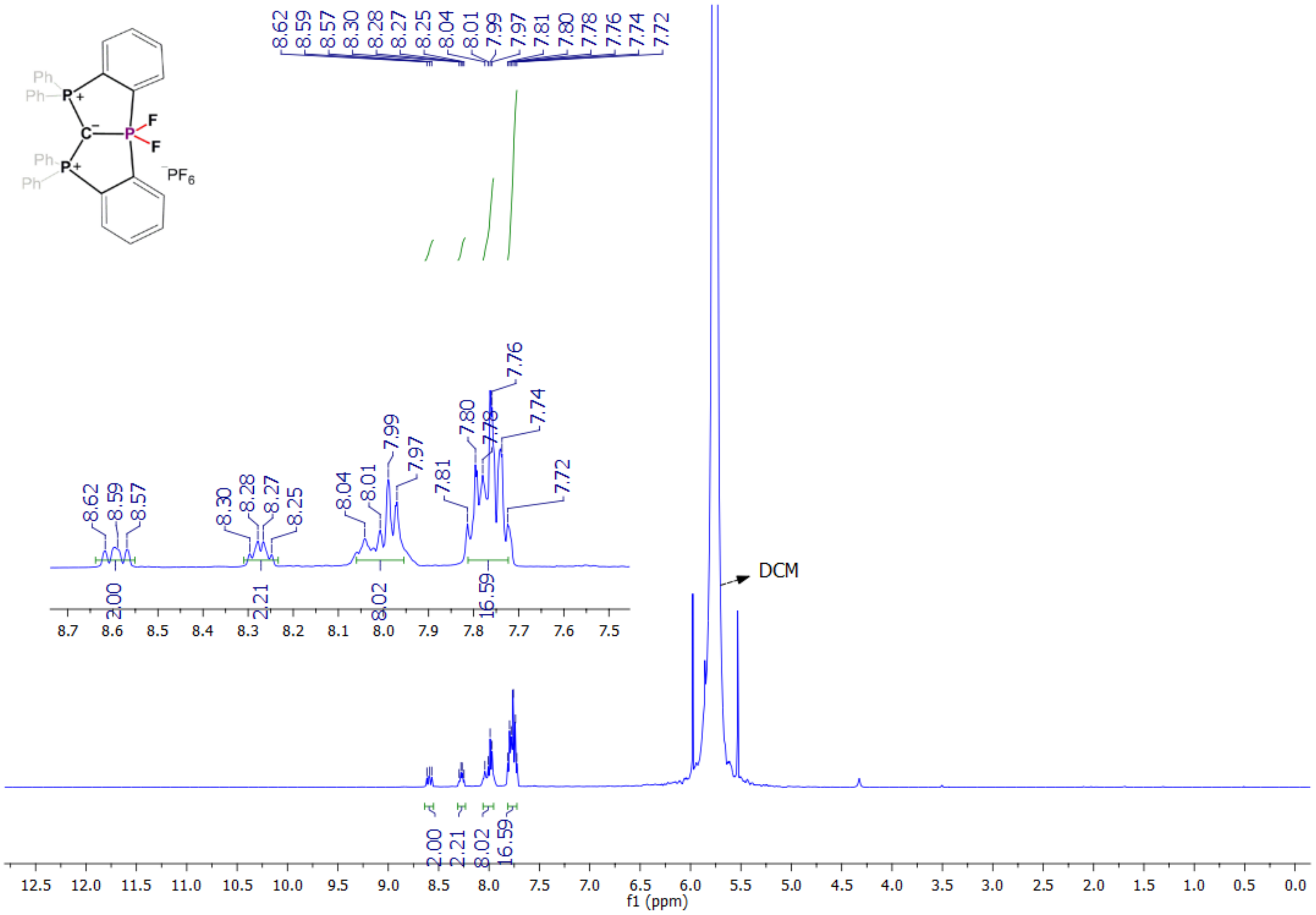

Figure S12. ^{1}H NMR spectrum (400 MHz, in DCM, DMSO-d_6 cap.) of [**1^+-F_2**][PF_6].

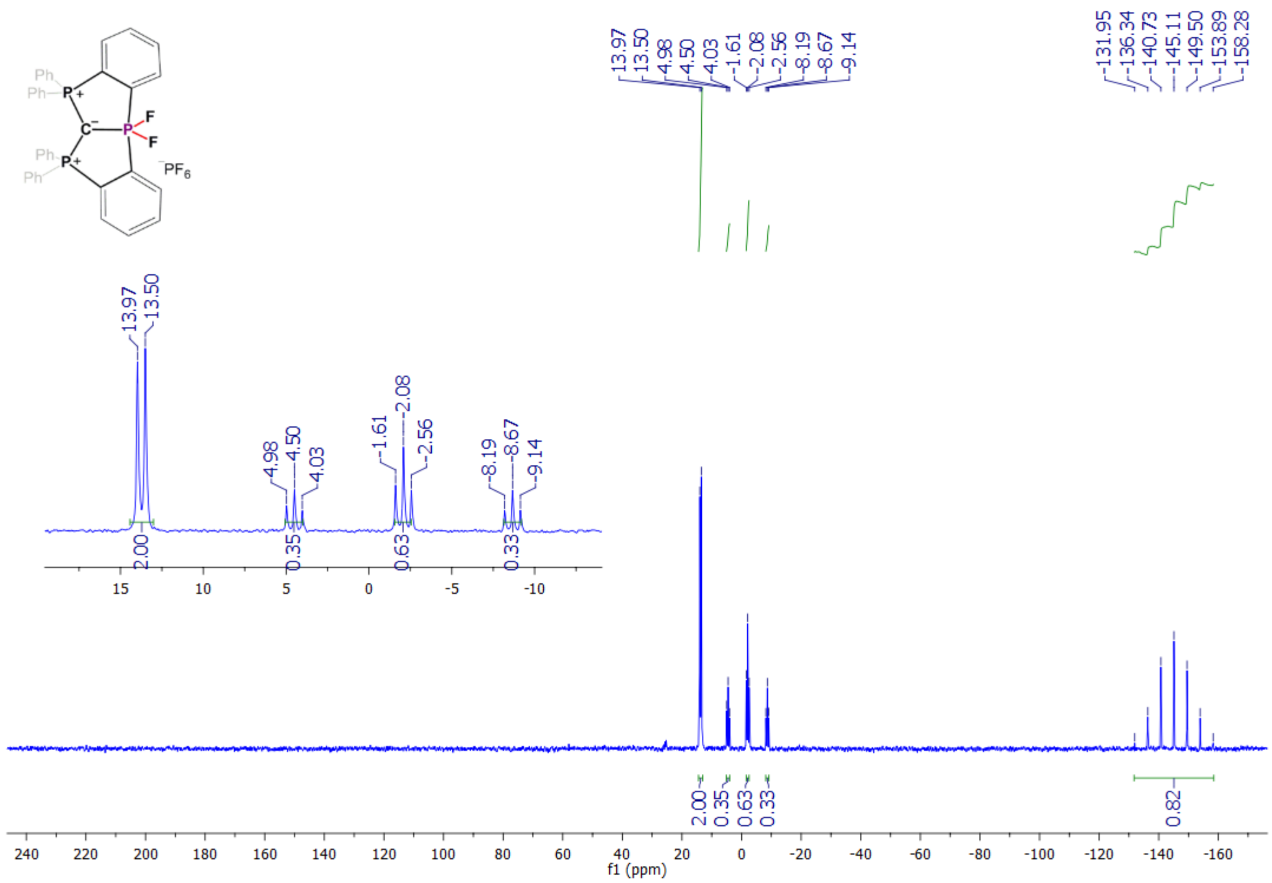

Figure S13. ^{31}P NMR spectrum (162 MHz, in DCM, DMSO-d$_6$ cap.) of [**1$^+$-F$_2$**][PF$_6$].

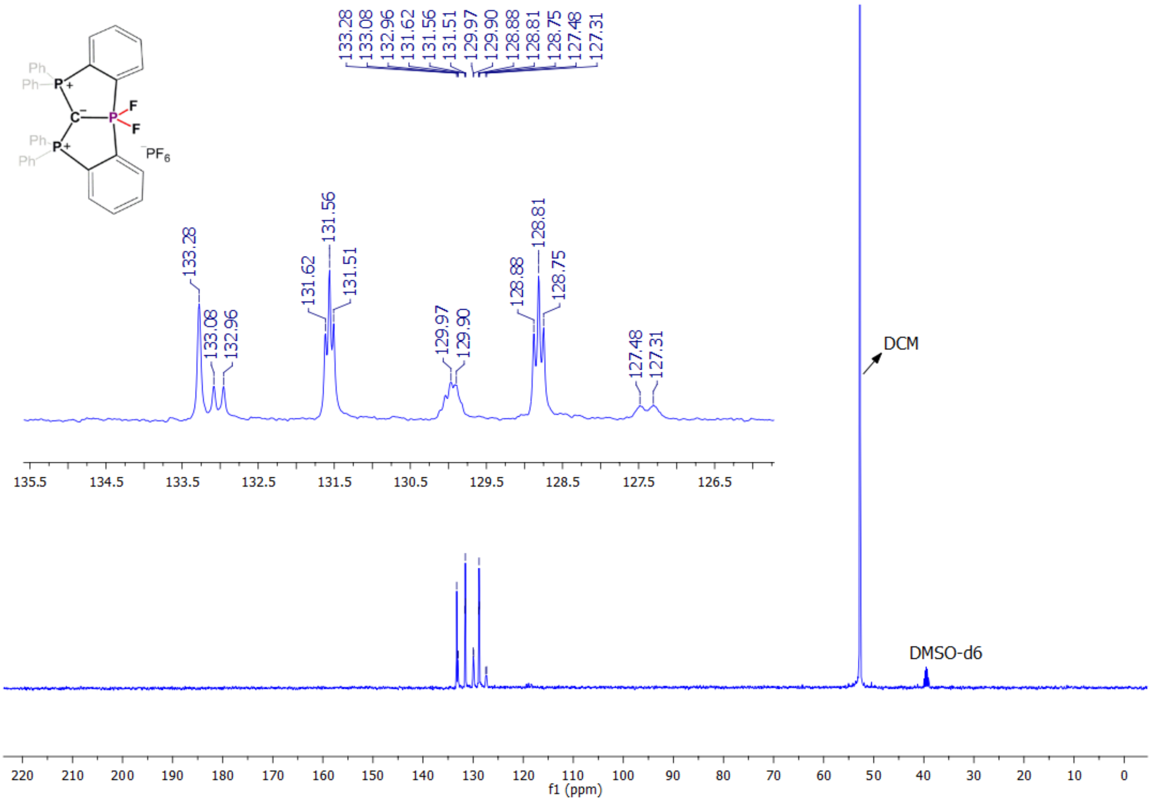

Figure S14. ^{13}C NMR spectrum (100 MHz, in DCM, DMSO-d$_6$ cap.) of [**1$^+$-F$_2$**][PF$_6$].

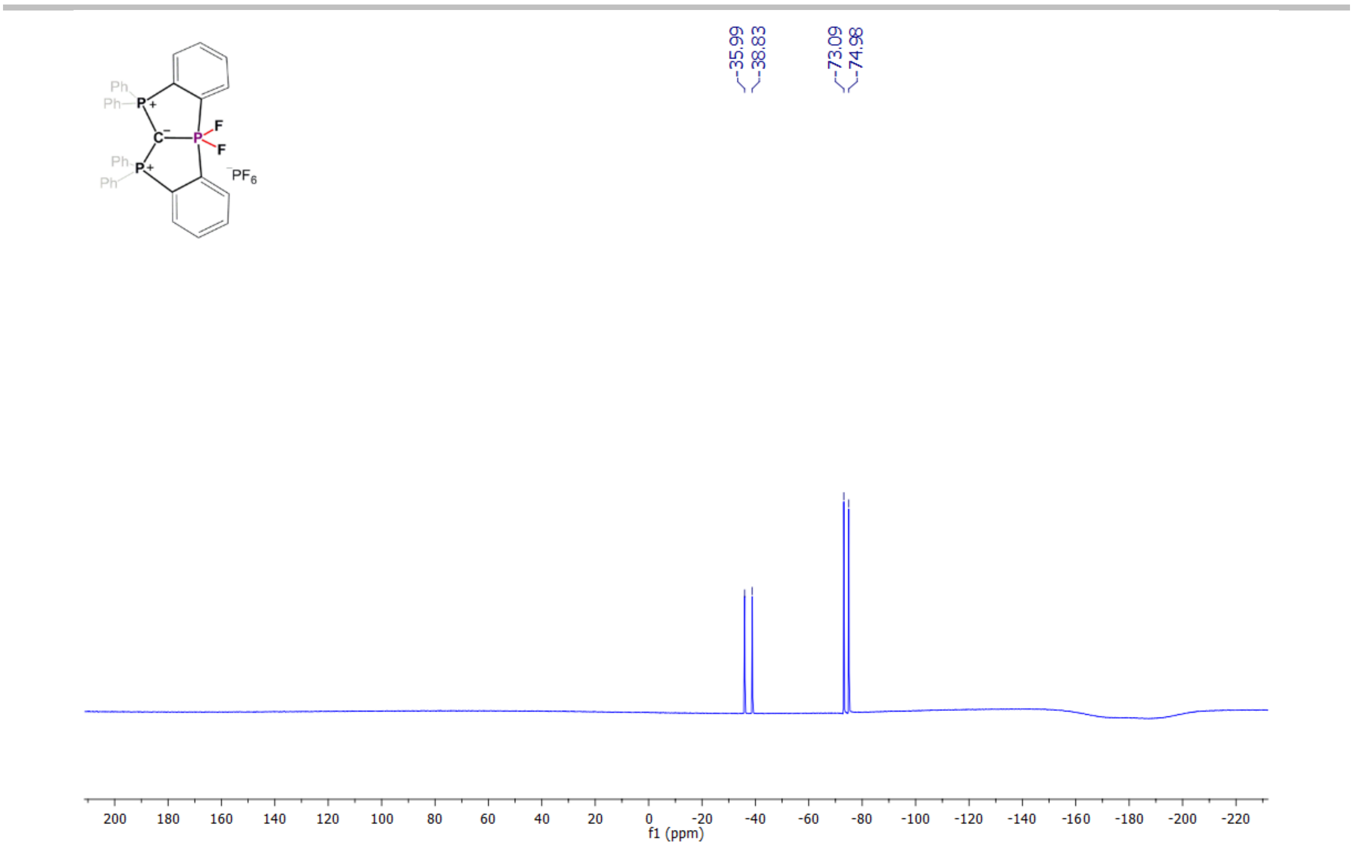

Figure S15. ^{19}F NMR spectrum (376.5 MHz, in DCM, DMSO-d_6 cap.) of [**1$^+$-F$_2$**][PF_6].

NMR data of [1$^+$-O][PF$_6$]: ^{1}**H-NMR** (400 MHz, $CDCl_3$), *δ*: 6.94 (m, 4H), 7.13 (t, *J* = 8 Hz, 4H), 7.43 (t, *J* = 8 Hz, 2H), 7.66-771 (m, 8H), 7.76 (t, *J* = 8 Hz, 2H), 7.90 (m, 2H), 8.08 (m, 4H), 8.39 (t, *J* = 8 Hz) ppm. ^{13}**C-NMR** (100 MHz, $CDCl_3$), *δ*: 117.59 (d, *J* = 103 Hz), 125.84 (d, *J* = 103 Hz), 130.32, 132.59, 133.75, 134.50, 134.98, 140.95 (d, *J* = 110 Hz) ppm. ^{31}**P-NMR** (162 MHz, $CDCl_3$): *δ* = -144.45 (sep, *J* = 711 Hz, *P*F$_6$), 26.18 (d, J = 52 Hz ,C*P*$_2$), 59.68 (t, *J* = 52 Hz, *P*=O) ppm. ^{19}**F-NMR** (376.5 MHz, $CDCl_3$), *δ*: -73.55 (d, *J* = 711 Hz, P*F*$_6$) ppm. HRMS Calculated for $C_{37}H_{28}OP_3$: 581.1353 [M-PF$_6$]$^+$; Obs: 581.1362.

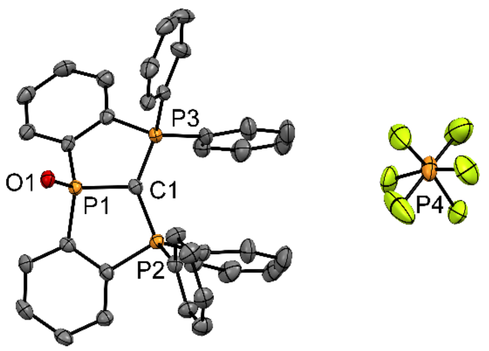

Figure S16. Single crystal X-ray structure of [**1-O$^+$**][PF_6]. Thermal ellipsoids at 50% probability, hydrogen atoms were omitted for clarity.

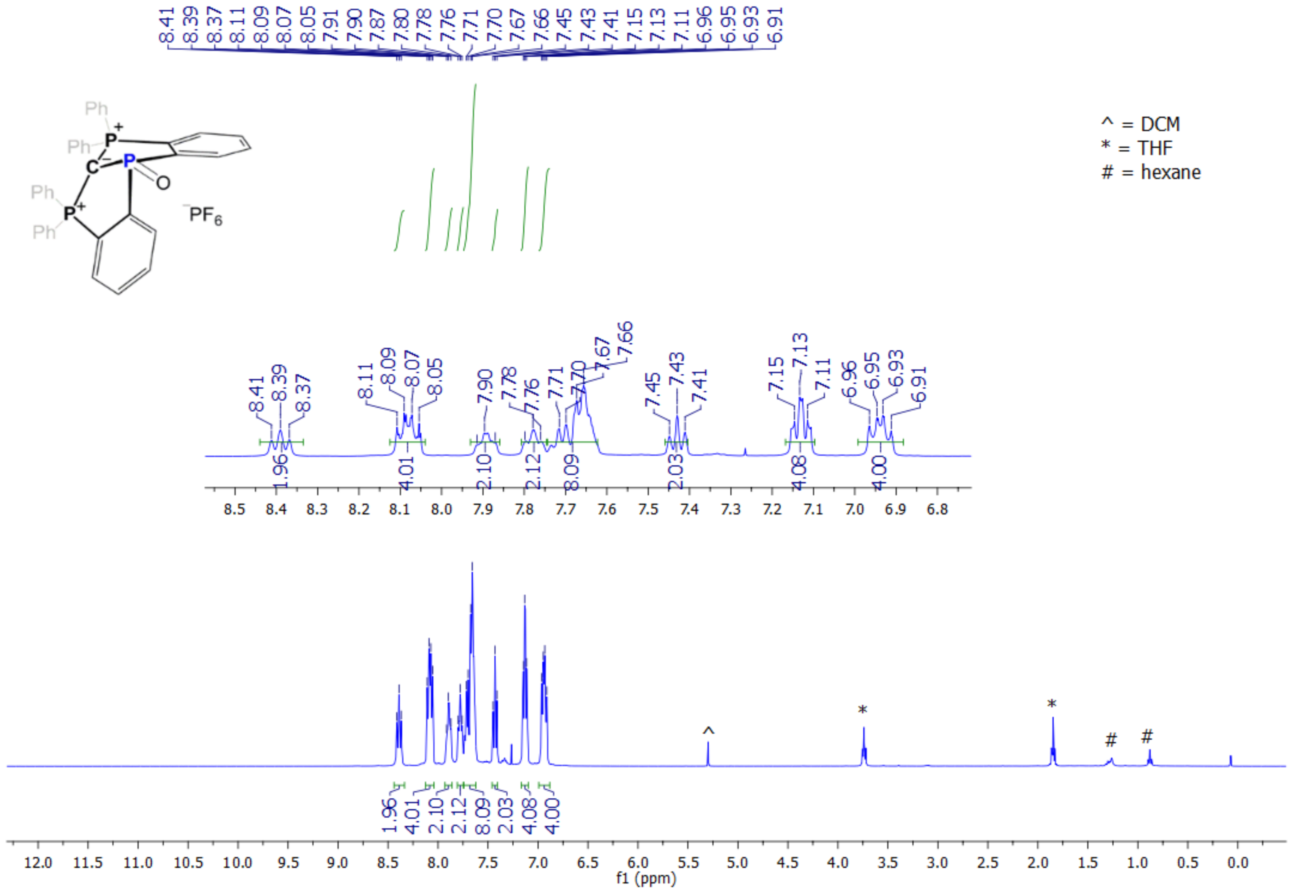

Figure S17. ^{1}H NMR spectrum (400 MHz, $CDCl_3$) of [**1$^+$-O**][PF_6].

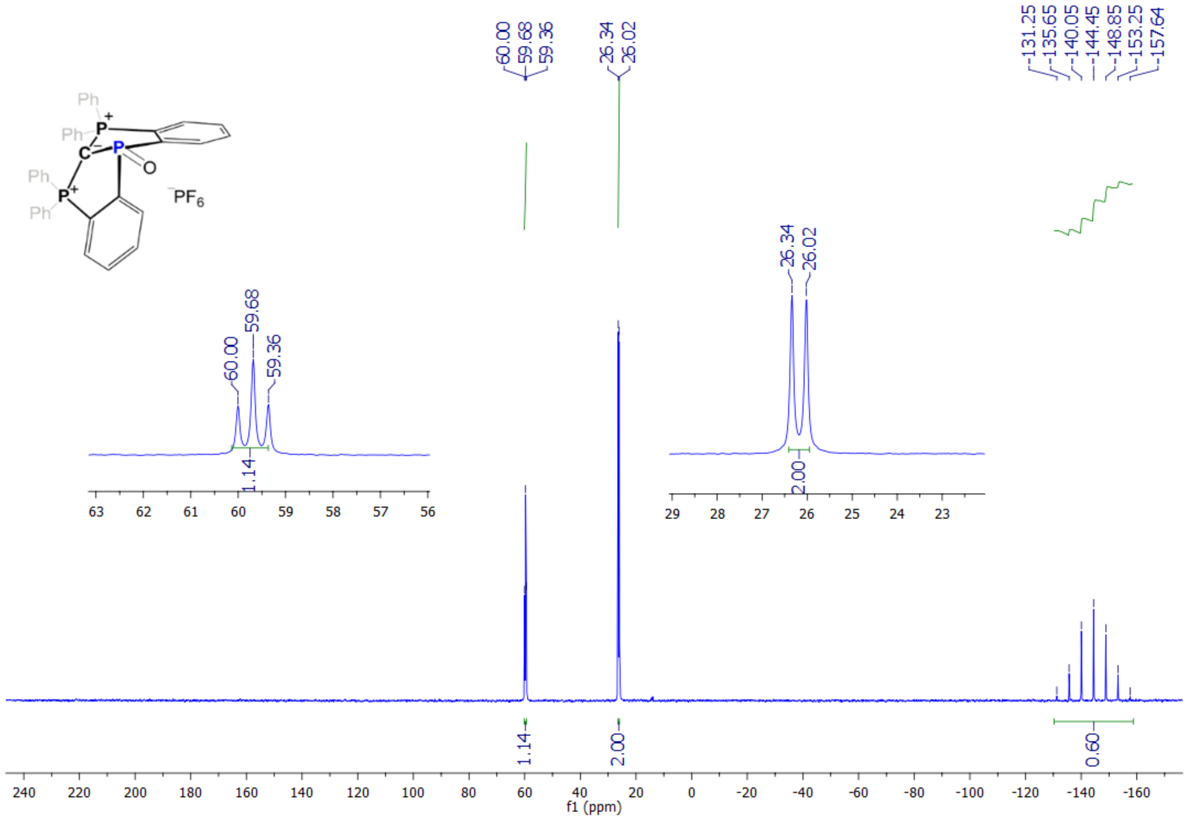

Figure S18. ^{31}P NMR spectrum (162 MHz, $CDCl_3$) of [**1$^+$-O**][PF_6].

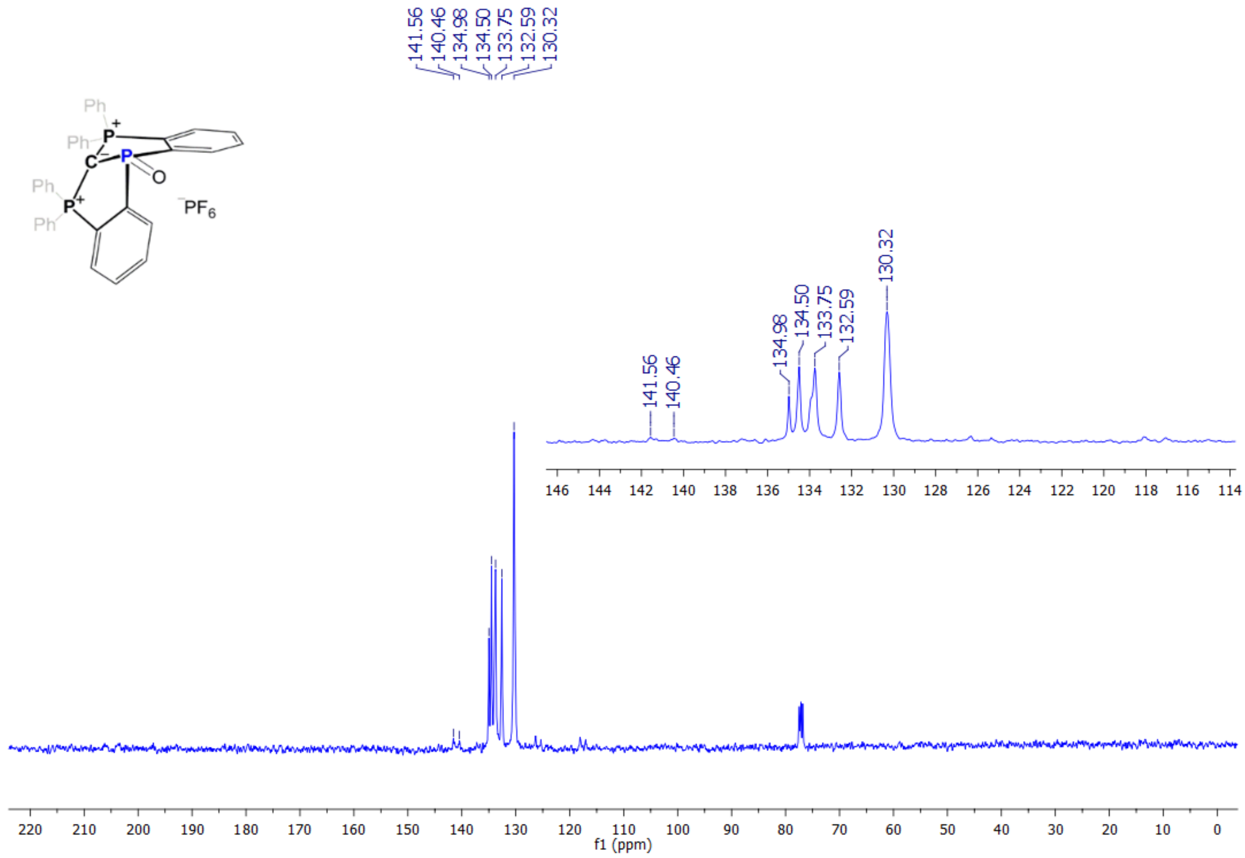

Figure S19. ^{13}C NMR spectrum (100 MHz, $CDCl_3$) of [**1$^+$-O**][PF_6].

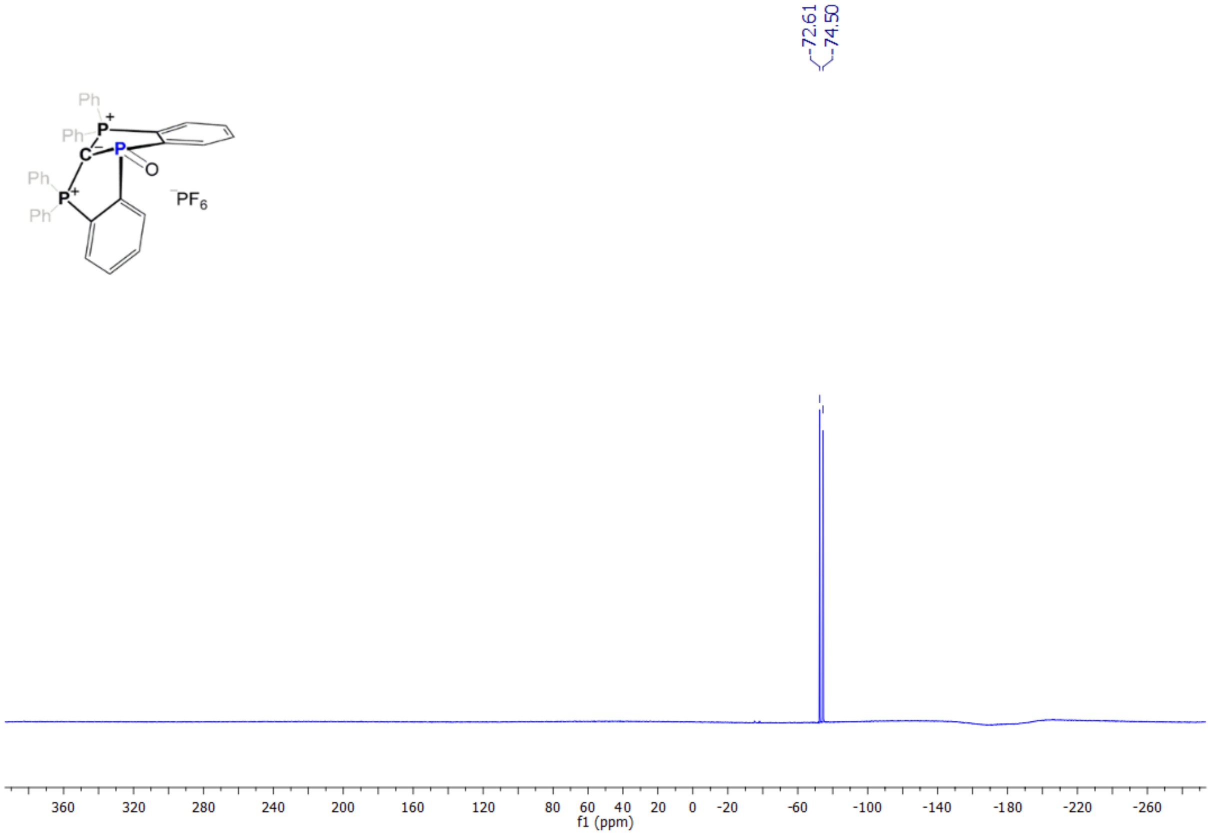

Figure S20. ^{19}F NMR spectrum (376.5 MHz, $CDCl_3$) of [**1$^+$-O**][PF_6].

Perfluoro-4,4'-bipyridine (5): ^{19}F-NMR (376.5 MHz, $CDCl_3$), δ: -137.87 (s, 2F), -87.12 (s, 2F) ppm.[8]

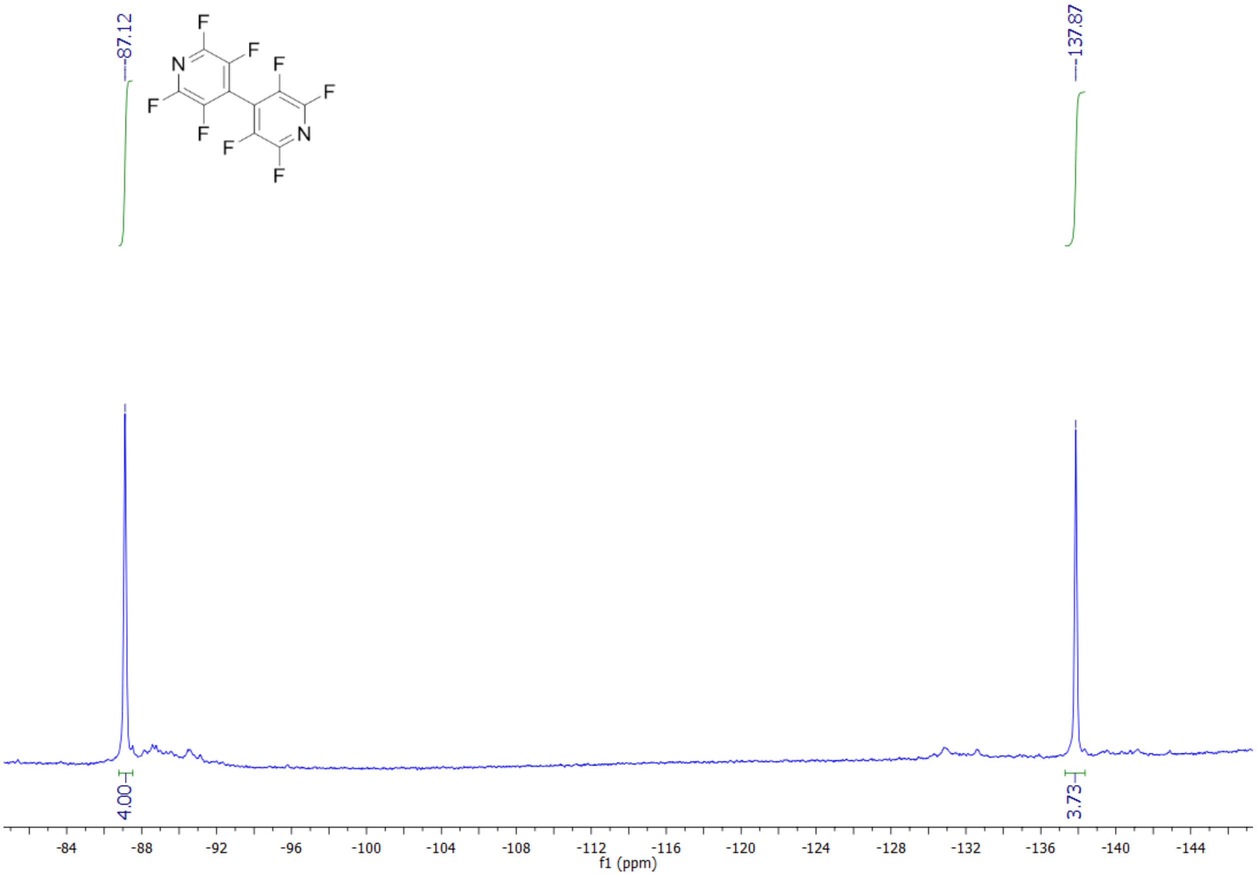

Figure S21. ^{19}F NMR spectrum (376.5 MHz, in $CDCl_3$) of **5**.

General procedure:

0.1 equiv. of [**1**$^+$][PF_6] was dissolved in *o*DFB. Next, 1.0 equivalent of Ar-F and 1.0 equivalent of $PhSiH_3$ (it is important to note that only $PhSiF_3$ was observed in the ^{19}F NMR spectra after reaction completion, meaning that 0.3 equivalent of $PhSiH_3$ is consumed in these reactions) were added to the solution and transferred to a J-Young NMR tube. The tube was heated to monitor the progress of reaction (conditions specified in Table 1). After completion, the solution was evaporated and fully dried. The residue was dissolved in n-hexane and the resulting mixture was passed through celite column. Hexane was then evaporated and the product thoroughly dried. If the hydrodefluorination product is volatile, the reaction mixture was analyzed by quantitative ^{19}F NMR with a sealed glass capillary containing DMSO-d_6.

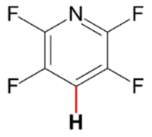

2,3,5,6-tetrafluoropyridine (7): **^{19}F-NMR** (376.5 MHz, in *o*-DFB, DMSO cap.), *δ*: -141.00 (s, 2F), -92.57 (s, 2F) ppm. NMR data is consistent with the reported literature.[9]

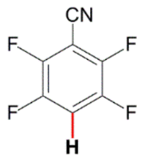

2,3,5,6-tetrafluorobenzonitrile (8): **^{19}F-NMR** (376.5 MHz, in *o*-DFB, DMSO cap.), *δ*: -136.78 (s, 2F), -134.57 (s, 2F) ppm. NMR data is consistent with the reported literature.[9]

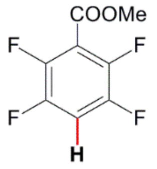

Methyl 4-(diethylamino)-2,3,5,6-tetrafluorobenzoate (9): ^{19}F-NMR (376.5 MHz, $CDCl_3$), *δ*: -139.44 (s, 2F), -137.36 (s, 2F) ppm. NMR data is consistent with the reported literature.[9]

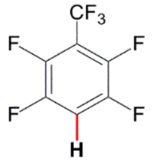

1,2,4,5-tetrafluoro-3-(trifluoromethyl)benzene (10): **^{19}F-NMR** (376.5 MHz, in *o*-DFB, DMSO cap.), *δ*: -141.93 (s, 2F), -137.68 (s, 2F), -57.49 (t, *J* = 22.7 Hz, 3F) ppm. NMR data is consistent with the reported literature.[9]

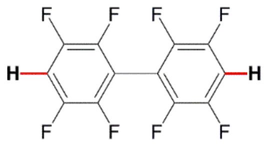

2,2',3,3',4,5,5',6,6'-nonafluoro-1,1'-biphenyl (11): ^{19}F-NMR (376.5 MHz, $CDCl_3$), *δ*: -138.36 (s, 2F), -137.82 (s, 2F) ppm. NMR data is consistent with the reported literature.[9]

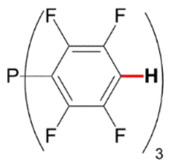

Tris(2,3,5,6-tetrafluorophenyl)phosphane (12): ^{19}F-NMR (376.5 MHz, $CDCl_3$), *δ*: -137.07 (m, 2F), -129.94 (m, 2F) ppm. NMR data is consistent with the reported literature.[9]

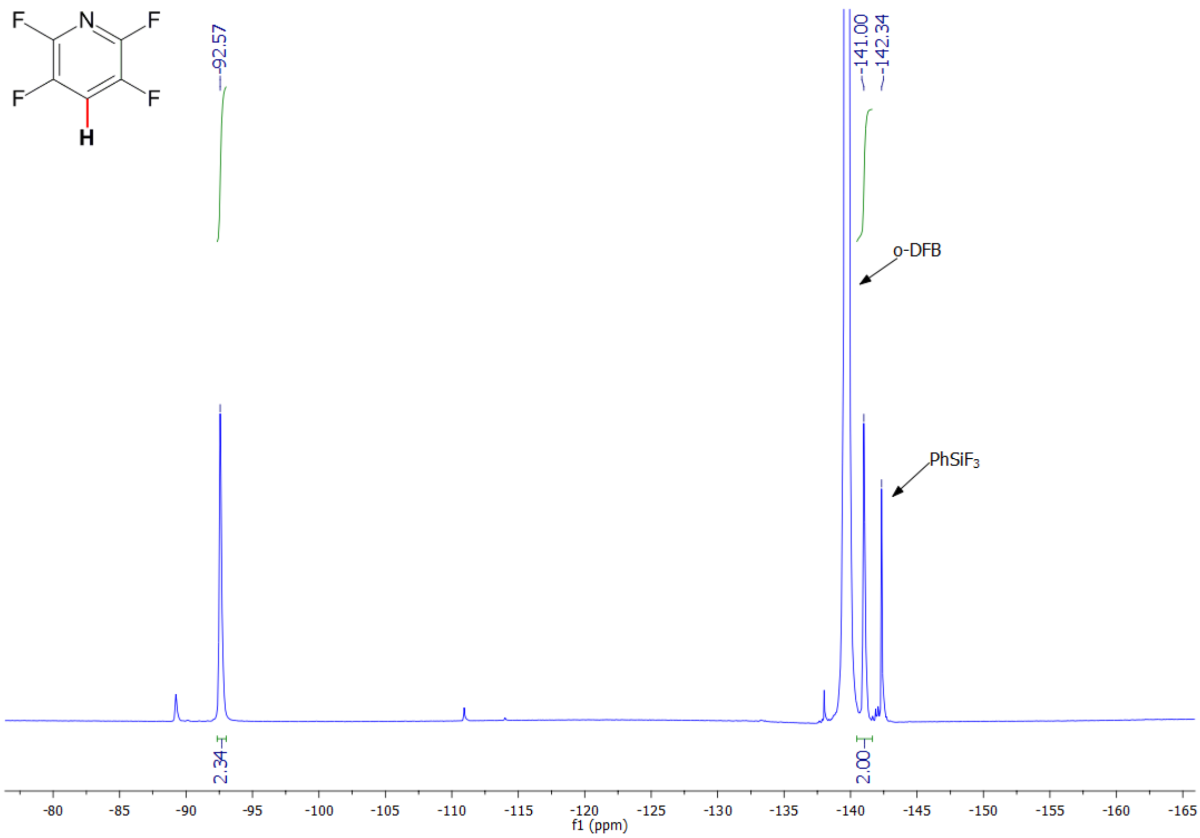

Figure S22. ^{19}F NMR spectrum (376.5 MHz, *o*-DFB) of **7**.

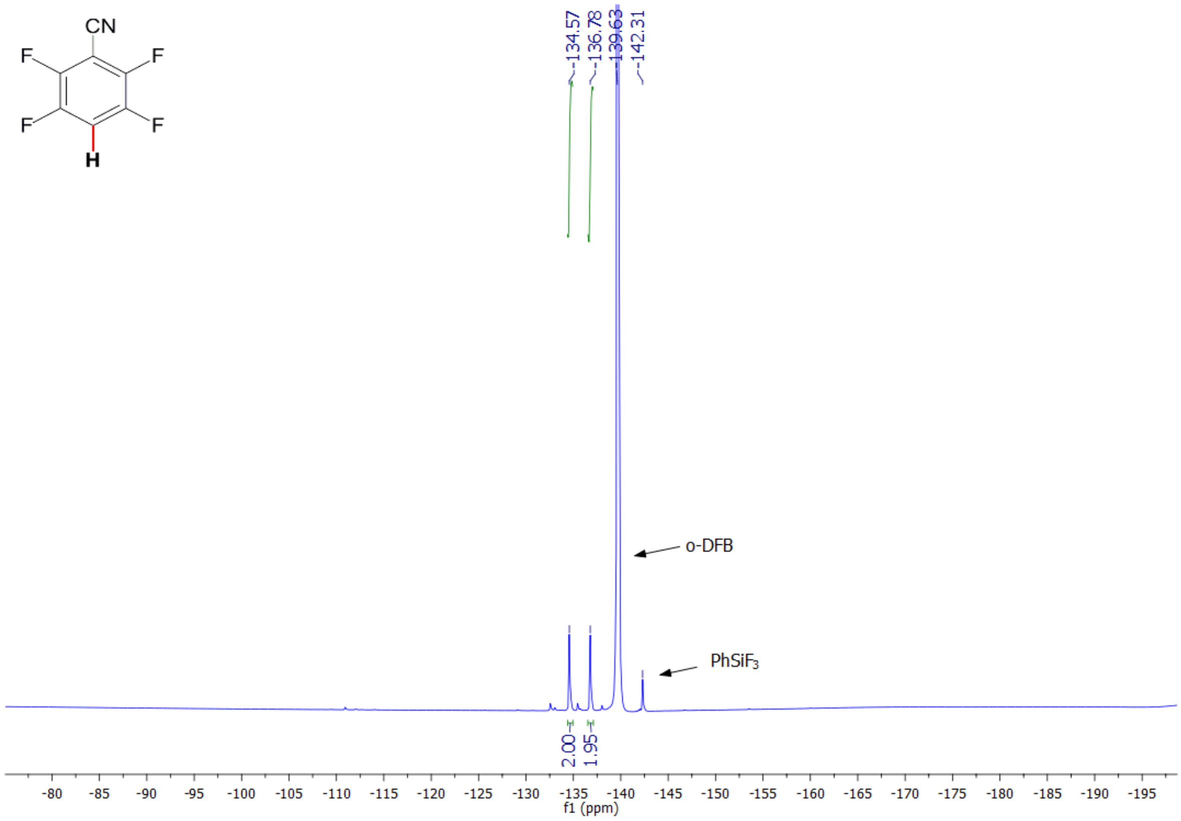

Figure S23. ^{19}F NMR spectrum (376.5 MHz, *o*-DFB) of **8.**

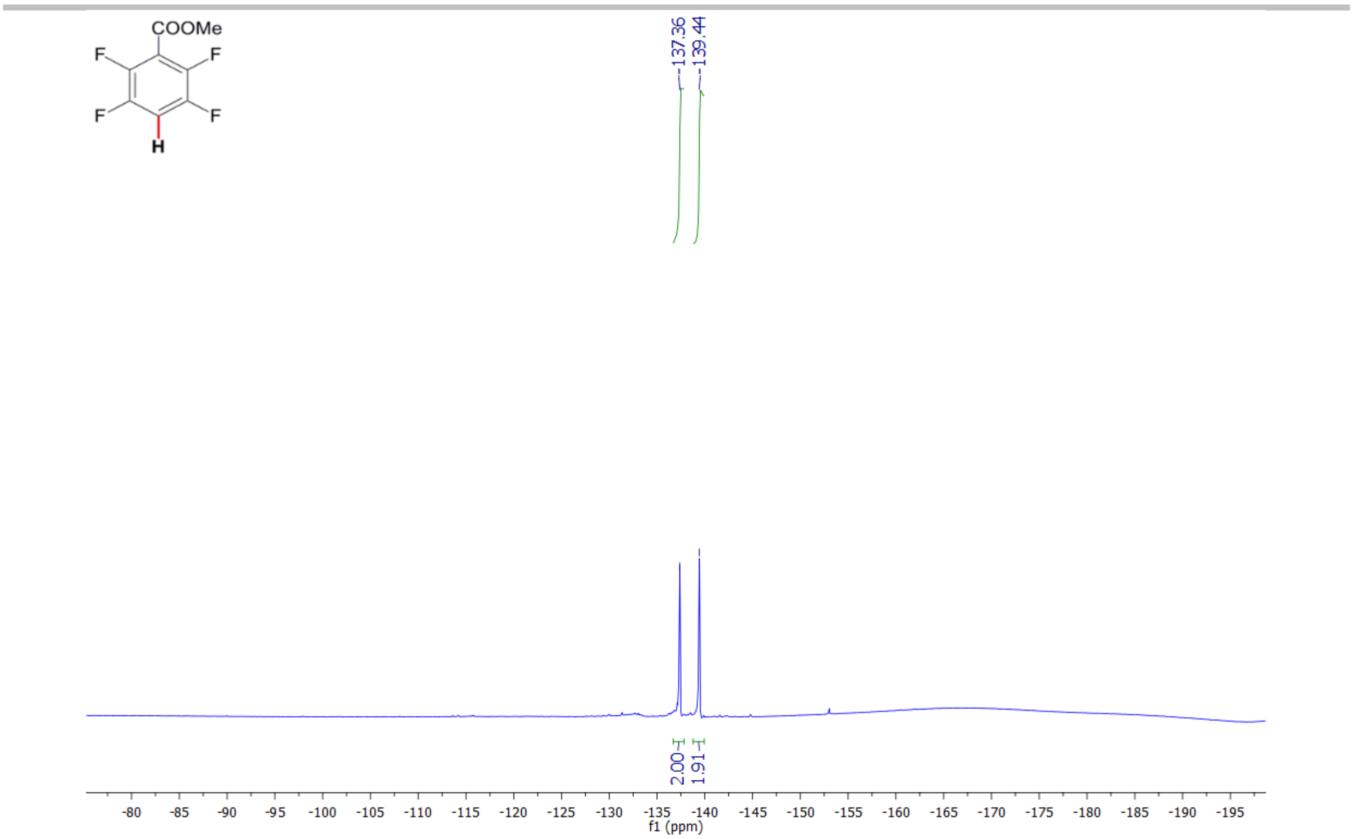

Figure S24. ^{19}F NMR spectrum (376.5 MHz, $CDCl_3$) of **9.**

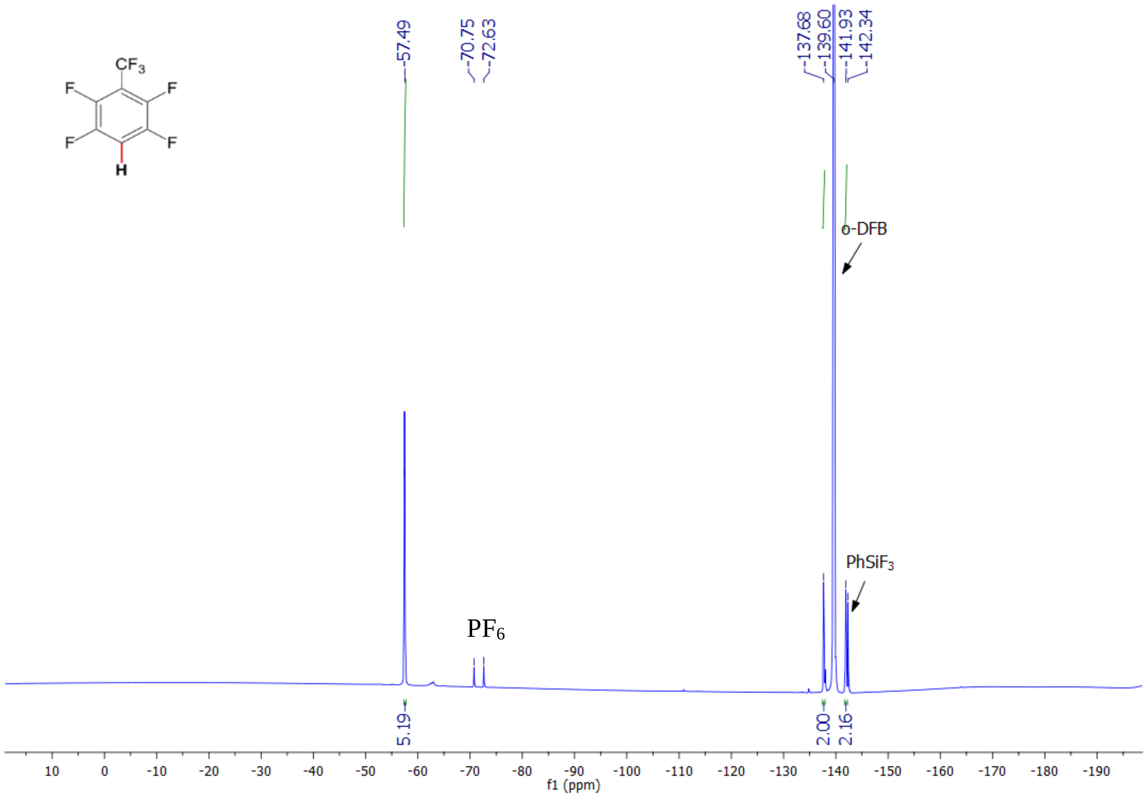

Figure S25. ^{19}F NMR spectrum (376.5 MHz, *o*-DFB) of **10**.

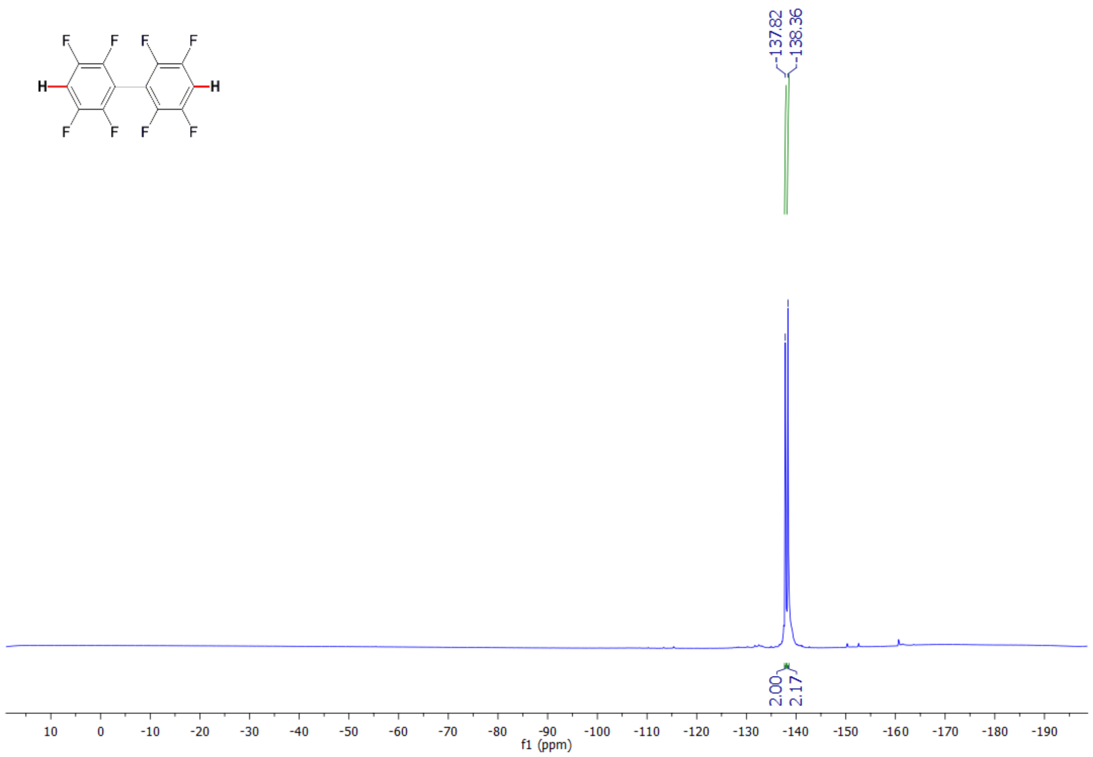

Figure S26. ^{19}F NMR spectrum (376.5 MHz, $CDCl_3$) of **11**.

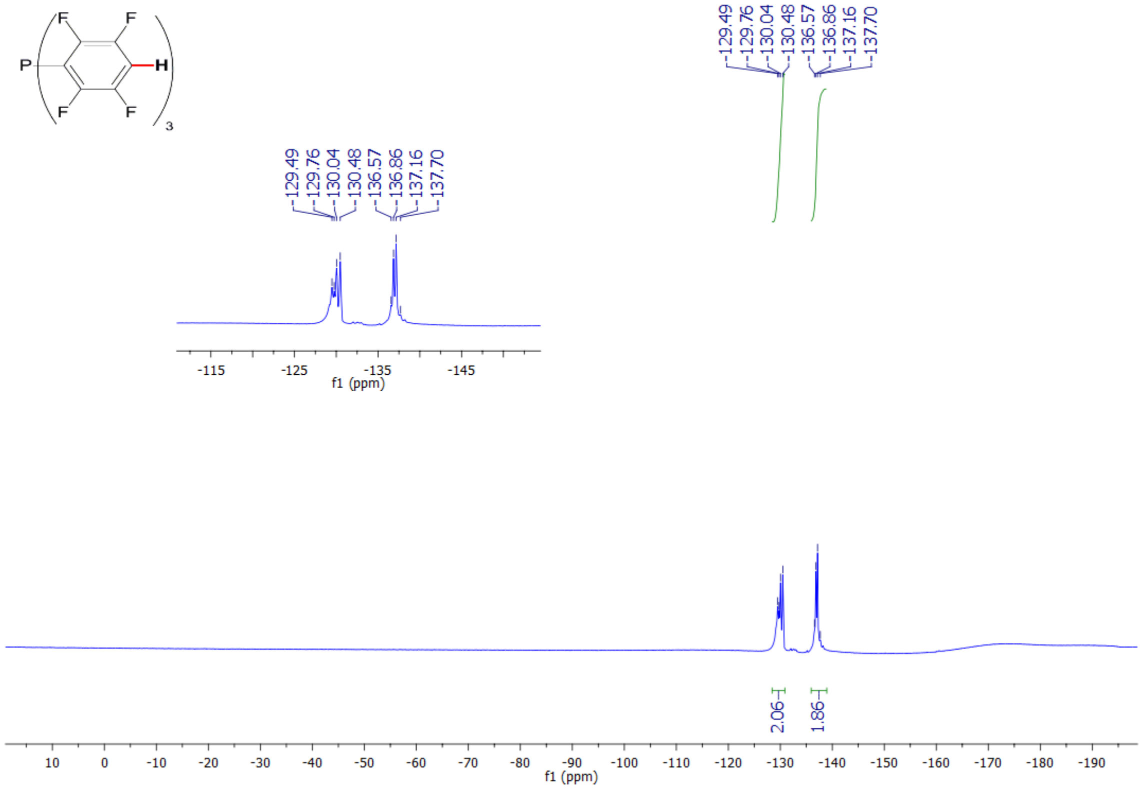

Figure S27. ^{19}F NMR spectrum (376.5 MHz, $CDCl_3$) of **12**.

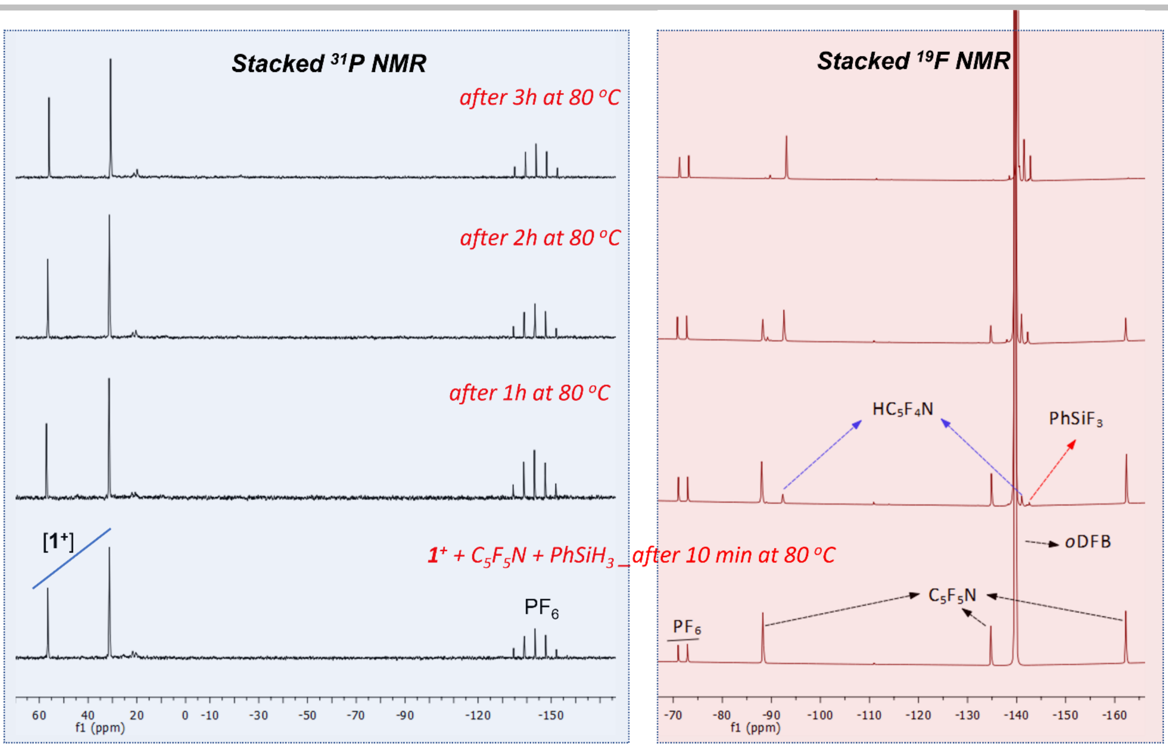

Figure S28. Stacked ^{31}P (162 MHz) and ^{19}F (376.5 MHz) NMR spectra (in *o*DFB) recorded at variable temperatures (VT) to show the resting state during the catalytic hydrodefluorination of C_5F_5N using [**1**$^+$][PF_6].

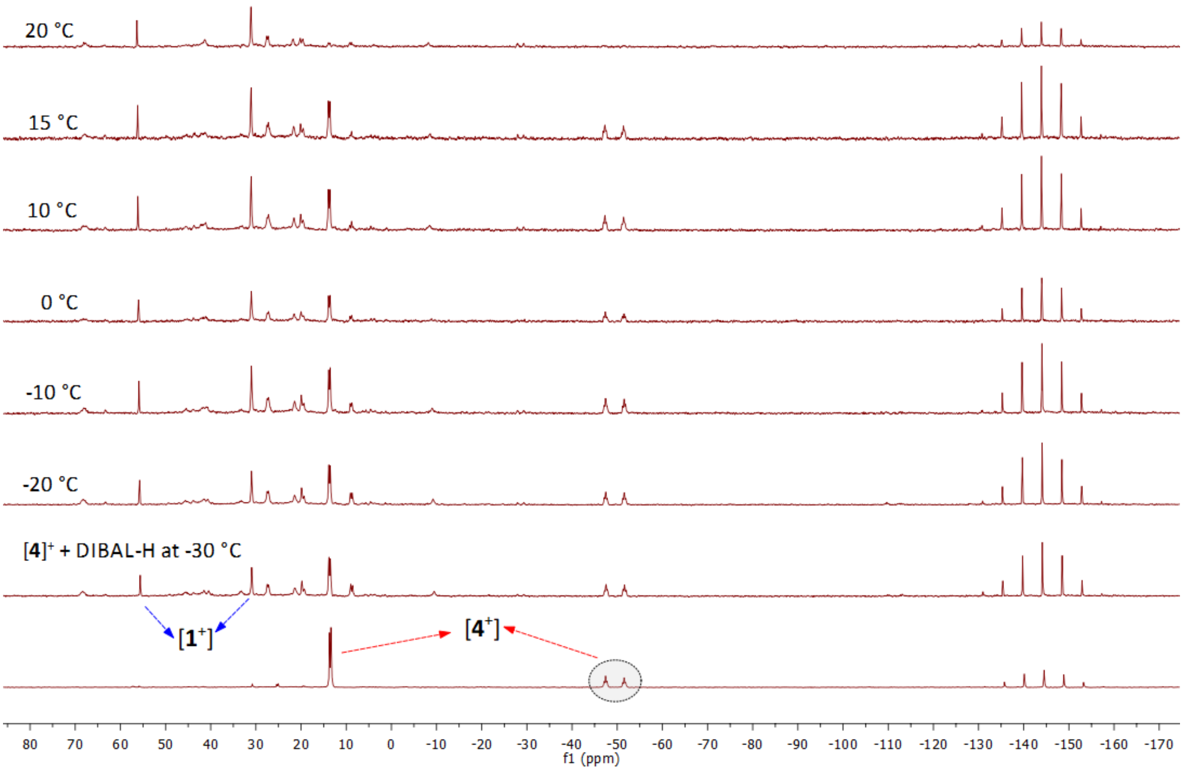

Figure S29. Stacked ^{31}P (162 MHz, in *o*DFB) NMR spectra recorded at variable temperatures (VT) to show the formation of [**1**$^+$][PF_6] from the reaction of [**4**$^+$][PF_6] with DIBAL-H.

Et_3Si-NEt_2:

Et_3Si-NEt_2 was synthesized according to the procedure described earlier.[10] **^{1}H-NMR** (400 MHz, $CDCl_3$), *δ*: 0.53 (q, *J* = 7.7 Hz, 6H), 0.94 (dt, *J* = 15.6, 7.3 Hz, 15H), 2.79 (q, *J* = 6.8 Hz, 4H), ppm. **^{13}C-NMR** (100 MHz, $CDCl_3$), δ: 4.83, 7.64, 16.10, 40.13 ppm. **^{1}H-^{29}Si HMBC NMR** (79.5 MHz, $CDCl_3$), *δ*: 8.8 ppm.

Et_3Si-N(H)Bn:

Et_3Si-N(H)Bn (Bn = $PhCH_2$) was synthesized according to the procedure described earlier.[10] **^{1}H-NMR** (500 MHz, $CDCl_3$), δ: 0.63 (q, *J* = 7.9 Hz, 6H), 0.73 (br-s, 1H), 1.01 (t, *J* = 7.9 Hz, 9H), 4.00 (d, *J* = 7.9 Hz, 2H), 7.25-7.39 (m, 5H) ppm; **^{13}C-NMR** (100 MHz, $CDCl_3$), δ: 4.78, 7.22, 46.16, 126.38, 126.95, 128.25, 144.58 ppm; **^{1}H-^{29}Si HMBC NMR** (79.5 MHz, $CDCl_3$), δ: 9.3 ppm.

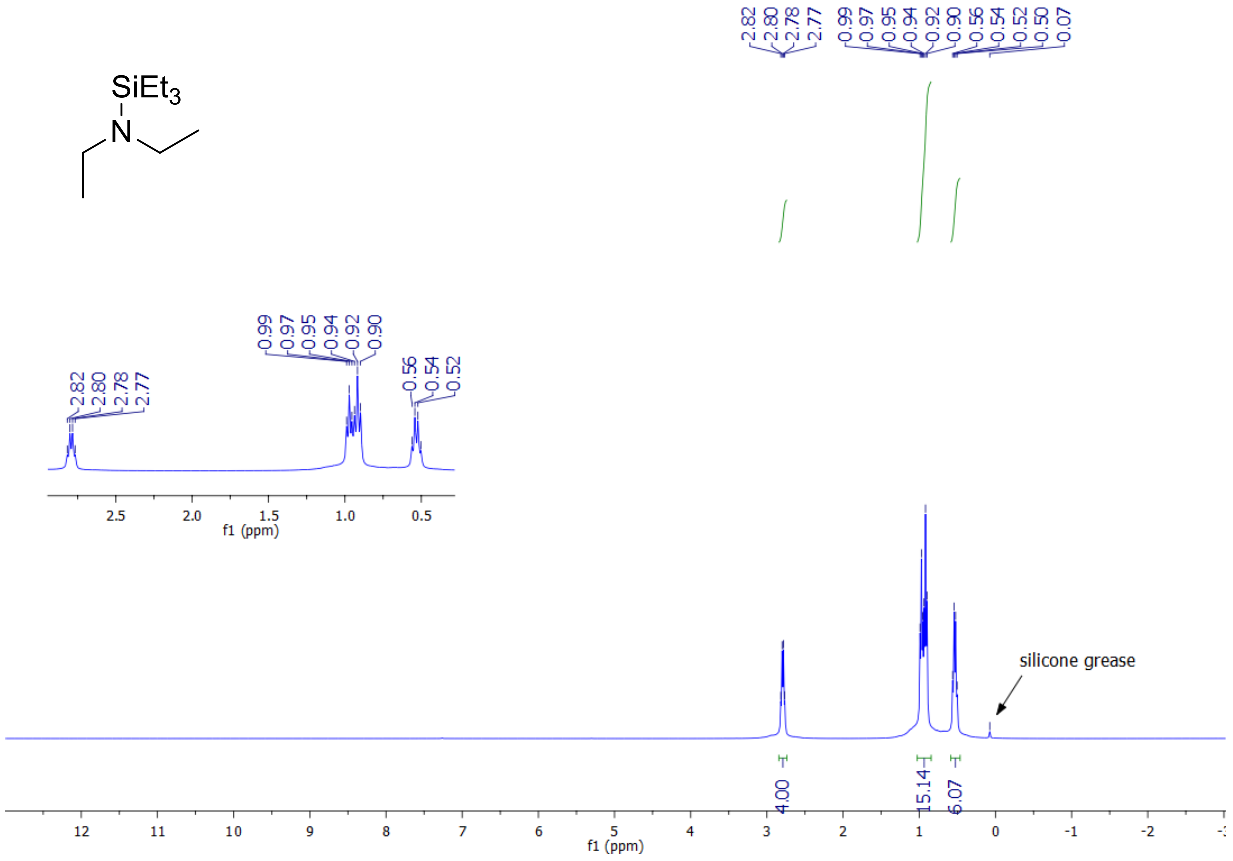

Figure S30. ^{1}H NMR spectrum (400 MHz, $CDCl_3$) of Et_3Si-NEt_2.

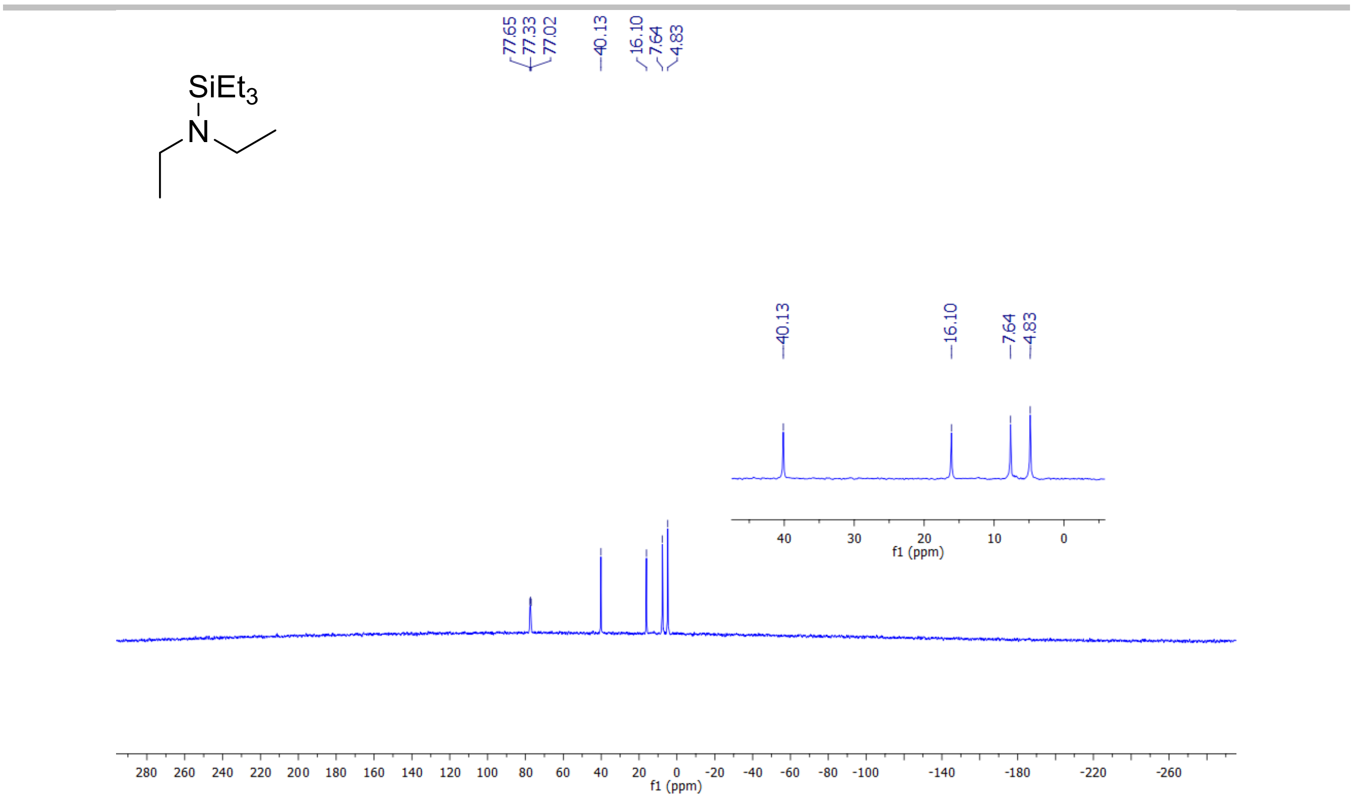

Figure S31. ^{13}C NMR spectrum (100 MHz, $CDCl_3$) of Et_3Si-NEt_2.

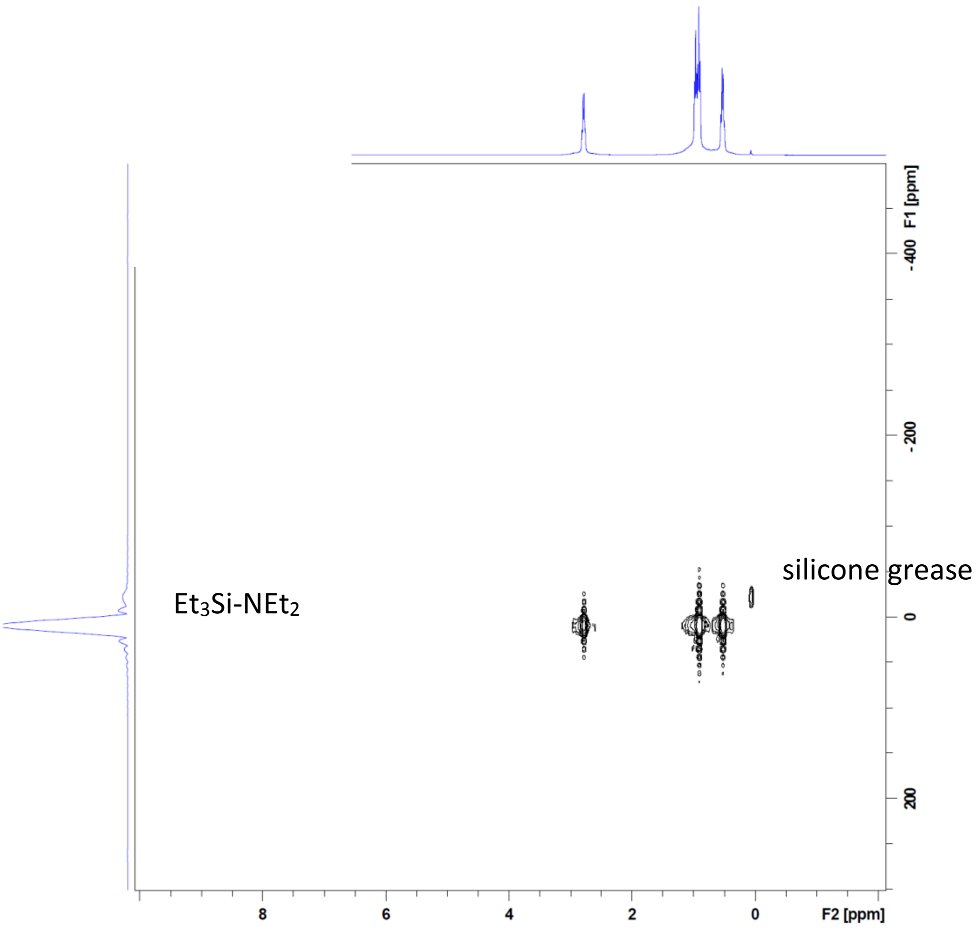

Figure S32. ^{1}H-^{29}Si HMBC NMR spectrum ($CDCl_3$) of Et_3Si-NEt_2.

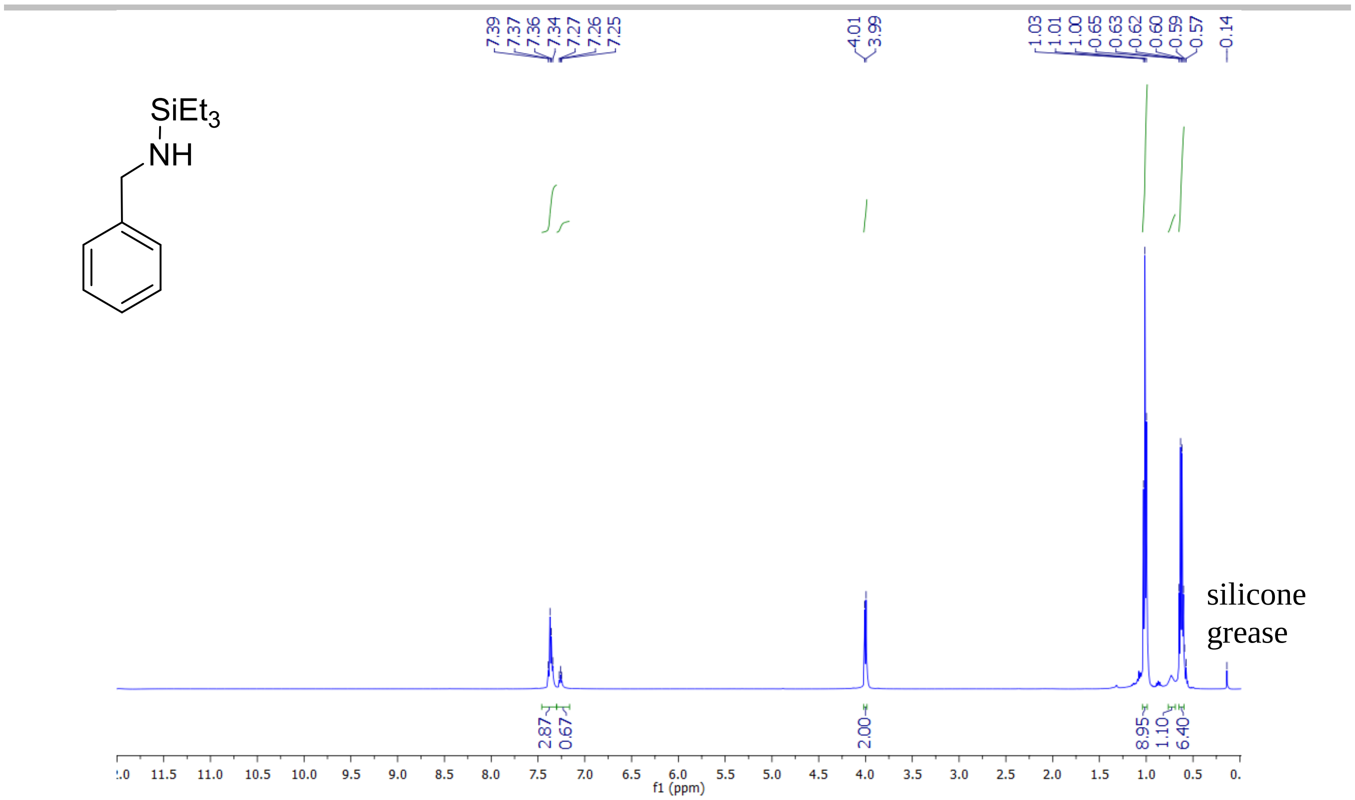

Figure S33. ^{1}H NMR spectrum (400 MHz, $CDCl_3$) of Et_3Si-N(H)Bn.

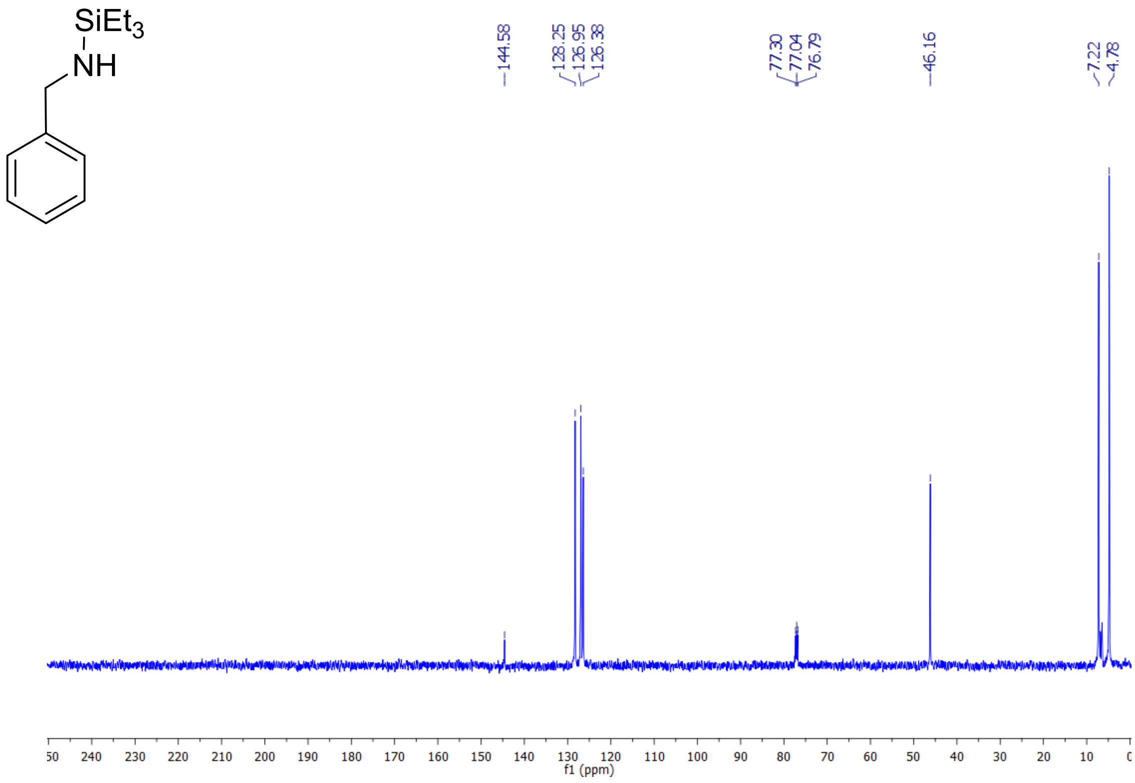

Figure S34. ^{13}C NMR spectrum (100 MHz, $CDCl_3$) of Et_3Si-N(H)Bn.

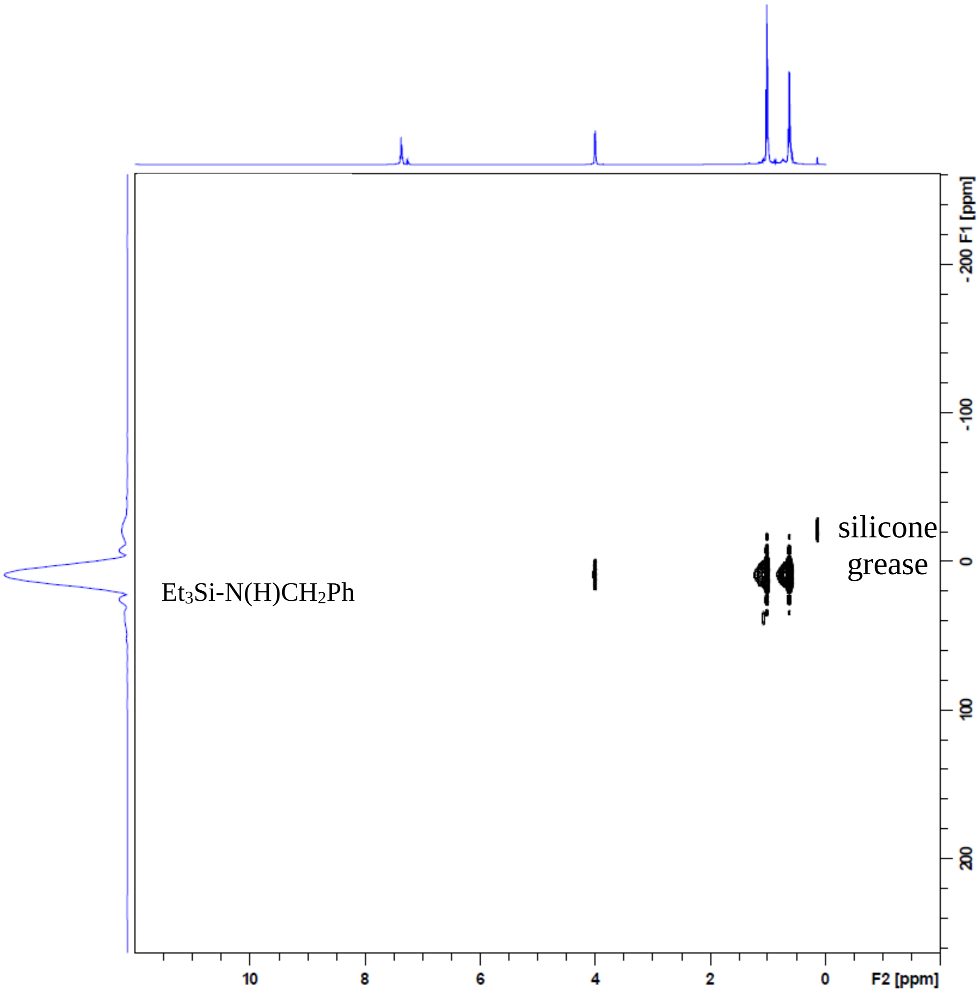

Figure S35. 1H-29Si HMBC NMR spectrum ($CDCl_3$) of Et_3Si-N(H)Bn.

General procedure:

0.1 equiv. of [**1**+][PF_6] was dissolved in *o*DFB. Next, 1.0 equivalent of Ar-F and 1.0 equivalent of Et_3Si-NEt_2 or Et_3Si-N(H)Bn were added to the solution and transferred to a J-Young NMR tube. The tube was heated to monitor the progress of reaction (conditions specified in Table 2). After completion, the solution was evaporated and fully dried. The residue was dissolved in n-hexane and the resulting mixture was passed through celite column. Hexane was then evaporated and the product thoroughly dried.

N,N-diethyl-2,3,5,6-tetrafluoropyridin-4-amine (14):

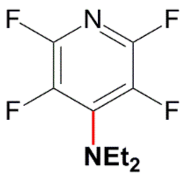

^{1}H-NMR (400 MHz, $CDCl_3$), δ: 1.21 (t, J = 7.1 Hz, 6H), 3.42 (qt, J = 7.0 Hz, 1.5 Hz, 4H) ppm. **^{13}C-NMR** (100 MHz, $CDCl_3$), δ: 13.79, 46.57 (t, J = 4 Hz), 134.30 (dm, J = 248.9 Hz), 139.37, 145.17 (dt, J = 238.4, 16.6 Hz) ppm. **^{19}F-NMR** (376.5 MHz, $CDCl_3$), δ: -94.67 (s, 2F), -156.29 (s, 2F) ppm. NMR data is consistent with the literature.[11]

N-benzyl-2,3,5,6-tetrafluoropyridin-4-amine (14a):

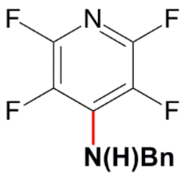

^{1}H-NMR (400 MHz, $CDCl_3$), *δ*: 4.71 (d, *J* = 5.7 Hz, 2H), 4.80 (bs, 1H), 7.32-7.42 (m, 5H) ppm. **^{13}C-NMR** (100 MHz, $CDCl_3$), *δ*: 48.85, 127.52, 128.29, 129.10, 131.32 (dm, *J* = 245.1 Hz), 137.52, 144.23 (dm, *J* = 240.8 Hz). **^{19}F-NMR** (376.50 MHz, $CDCl_3$), *δ*: -163.30 (s, 2F), -93.75 (s, 2F) ppm.

4-(diethylamino)-2,3,5,6-tetrafluorobenzonitrile (15):

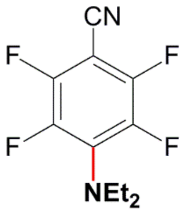

^{1}H-NMR (400 MHz, $CDCl_3$), *δ*: 1.18 (t, *J* = 7.1 Hz, 6H), 3.38 (q, *J* = 7.0 Hz, 4H) ppm. **^{13}C-NMR** (100 MHz, $CDCl_3$), *δ*: 13.84 (s), 46.94 (t, *J* = 4.3 Hz), 108.91 (s), 135.46 (s), 140.89 (dm, *J* = 242.1 Hz), 146.11 (s), 148.36 (dm, *J* = 259.2 Hz) ppm. **^{19}F-NMR** (376.5 MHz, $CDCl_3$), *δ*: -135.21 (s, 2F), -149.92 (s, 2F) ppm. NMR data is consistent with the literature.[12]

4-(benzylamino)-2,3,5,6-tetrafluorobenzonitrile (15a):

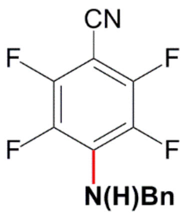

^{1}H-NMR (400 MHz, $CDCl_3$), *δ*: 4.68 (d, *J* = 6.1, 2H), 4.72 (bs, 1H), 7.31-7.41 (m, 5H) ppm. **^{13}C-NMR** (100 MHz, $CDCl_3$), *δ*: 49.32, 108.82, 127.53, 128.34, 129.13, 133.00, 136.03 (dm, *J* = 247.5 Hz), 137.47, 148.04 (dm, *J* = 258.39 Hz). **^{19}F-NMR** (376.5 MHz, $CDCl_3$), *δ*: -158.95 (s, 2F), -135.04 (s,2F) ppm.

Methyl 2-(diethylamino)-3,4,5,6-tetrafluorobenzoate (16):

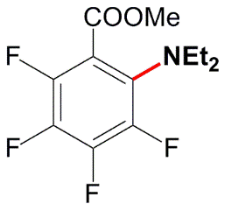

^{1}H-NMR (400 MHz, $CDCl_3$), *δ*: 0.98 (td, *J* = 7.2, 0.5 Hz, 6H), 3.05 (qd, *J* = 7.2, 1.3 Hz, 4H), 3.90 (s, 3H) ppm. **^{19}F-NMR** (376.5 MHz, $CDCl_3$), *δ*: -159.32 (t, *J* = 20.2 Hz, 1F), -154.37 (t, *J* = 18.6 Hz, 1F), -144.84 (s, 1F), -142.54 (s, 1F) ppm.

Methyl 4-(diethylamino)-2,3,5,6-tetrafluorobenzoate (17):

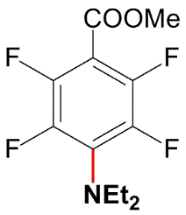

^{1}H-NMR (400 MHz, $CDCl_3$), *δ*: 1.12 (t, *J* = 7.1 Hz, 6H), 3.31 (qt, *J* = 7.1, 1.1 Hz, 4H), 3.92 (s, 3H) ppm. **^{13}C-NMR** (100 MHz, $CDCl_3$), *δ*: 13.54, 46.78 (p), 48.08 (o), 52.76 (p), 52.99 (o), 120.16, 132.75, 136.94-148.41 (m), 163. 33 ppm. **^{19}F-NMR** (376.5 MHz, $CDCl_3$), *δ*: -150.67 (d, *J* = 12.8 Hz, 2F), -140.94 (d, *J* = 12.7 Hz, 2F) ppm.

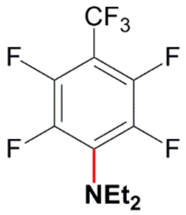

N,N-diethyl-2,3,5,6-tetrafluoro-4-(trifluoromethyl)aniline (18):

^{1}H-NMR (400 MHz, $CDCl_3$), *δ*: 1.14 (t, *J* = 7.1 Hz, 6H), 3.33 (q, *J* = 7.1 Hz, 4H) ppm. **^{13}C-NMR** (100 MHz, $CDCl_3$), *δ*: 13.63, 46.73, 120.24, 122.93, 133.26, 142.29 (d, *J* = 241.4 Hz), 145.11 (d, *J* = 269.2 Hz) ppm; **^{19}F-NMR** (376.5 MHz,

$CDCl_3$), δ: -55.42 (t, J = 19.5 Hz, 3F), -143.26 (t, J = 17.3 Hz, 2F), -149.96 (s, 2F) ppm. NMR data is consistent with the literature.[13]

N,N-diethyl-2,2',3,3',4',5,5',6,6'-nonafluoro-[1,1'-biphenyl]-4-amine (19):

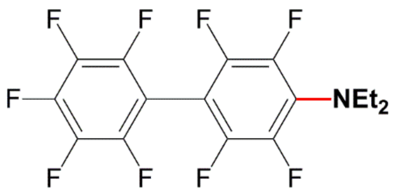

^{1}H-NMR (400 MHz, $CDCl_3$), δ: 1.15 (t, J = 7.1 Hz, 6H), 3.34 (q, J = 7.1 Hz, 4H) ppm. **^{13}C-NMR** (100 MHz, $CDCl_3$), δ: 13.15 (s), 47.30 (s), 98.31 (m), 103.52 (m), 132.16 (m), 138.71 (dm, J = 248.6 Hz), 142.90 (dm, J = 257.9 Hz), 144.00 (dm, J = 246.0 Hz), 145.45 (d, J = 248.2 Hz) ppm. **^{19}F-NMR** (376.5 MHz, $CDCl_3$), δ: -164.23 (s, 2F), -154.53 (s, 1F), -151.23 (s, 2F), -143.16 (s, 2F), -140.46 (s, 2F) ppm.

4-(bis(perfluorophenyl)phosphanyl)-N,N-diethyl-2,3,5,6-tetrafluoroaniline (20):

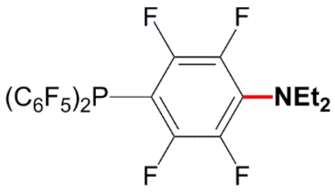

^{1}H-NMR (400 MHz, $CDCl_3$), δ: 1.14 (t, J = 7.1 Hz, 6H), 3.31 (q, J = 7.1, 4H) ppm. **^{13}C-NMR** (100 MHz, $CDCl_3$), δ: 13.50, 46.53, 97.69 (m), 106.11 (m), 132.96 (m), 137.63 (dm, J = 255.7 Hz), 142.43 (dd, J = 247.9, 15.2 Hz), 147.59 (d, J = 250.37 Hz), 148.47 (dm, J = 245.0 Hz). **^{19}F-NMR** (376.5 MHz, $CDCl_3$), δ: -160.08 (broad t, 4F), -149.53 (d, J = 14.0 Hz, 2F), -149.06 (broad t, 2F), -132. 89 (m, 2F), -130.49 (broad t, 4F). **^{31}P-NMR** (162 MHz, $CDCl_3$), δ: -75.13 (quin, J = 35.2, 1P). **HRMS** (AP+): Calculated for $C_{22}H_{11}NF_{14}P$: 586.0406 $[MH]^+$; Obs: 586.0408.

.

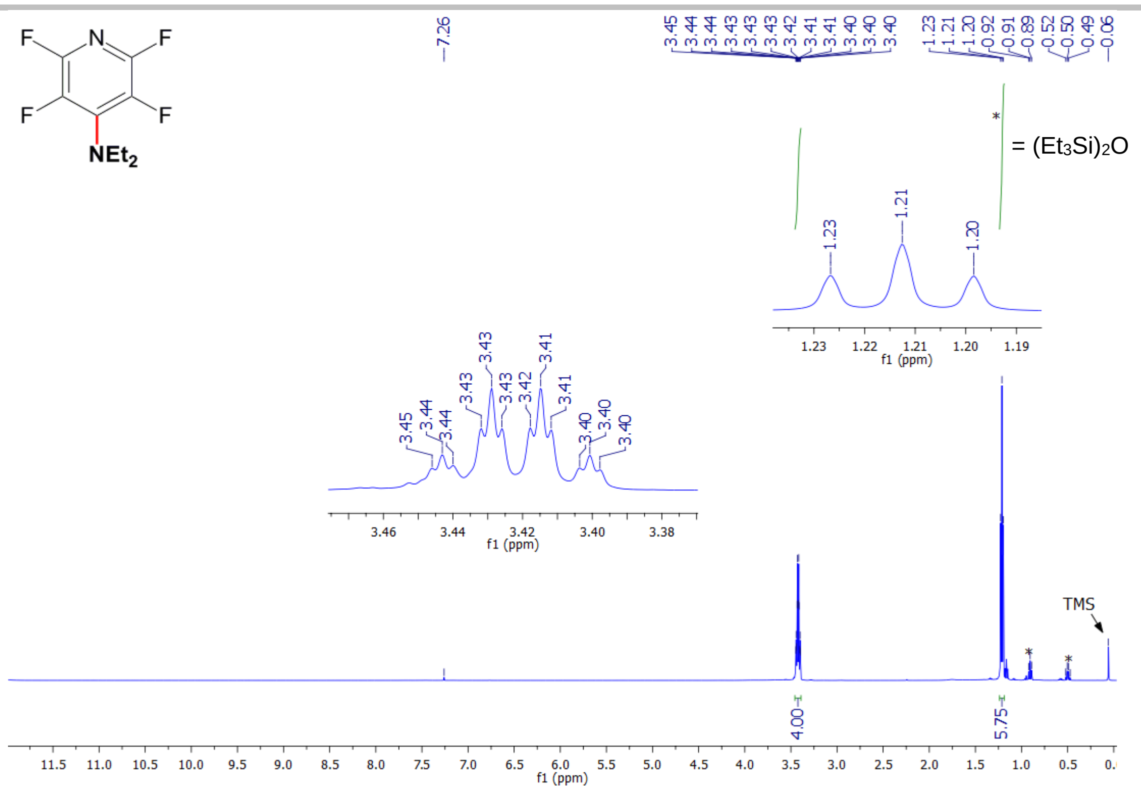

Figure S36. ^{1}H NMR spectrum (400 MHz, $CDCl_3$) of **14**.

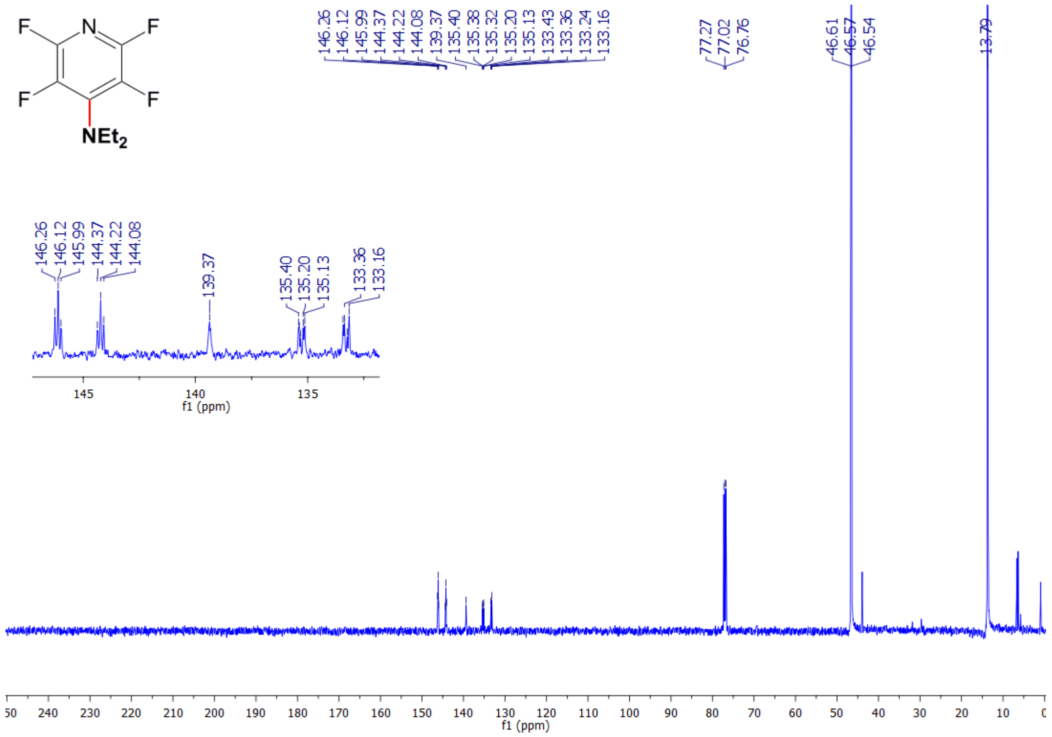

Figure S37. ^{13}C NMR spectrum (100 MHz, $CDCl_3$) of **14**.

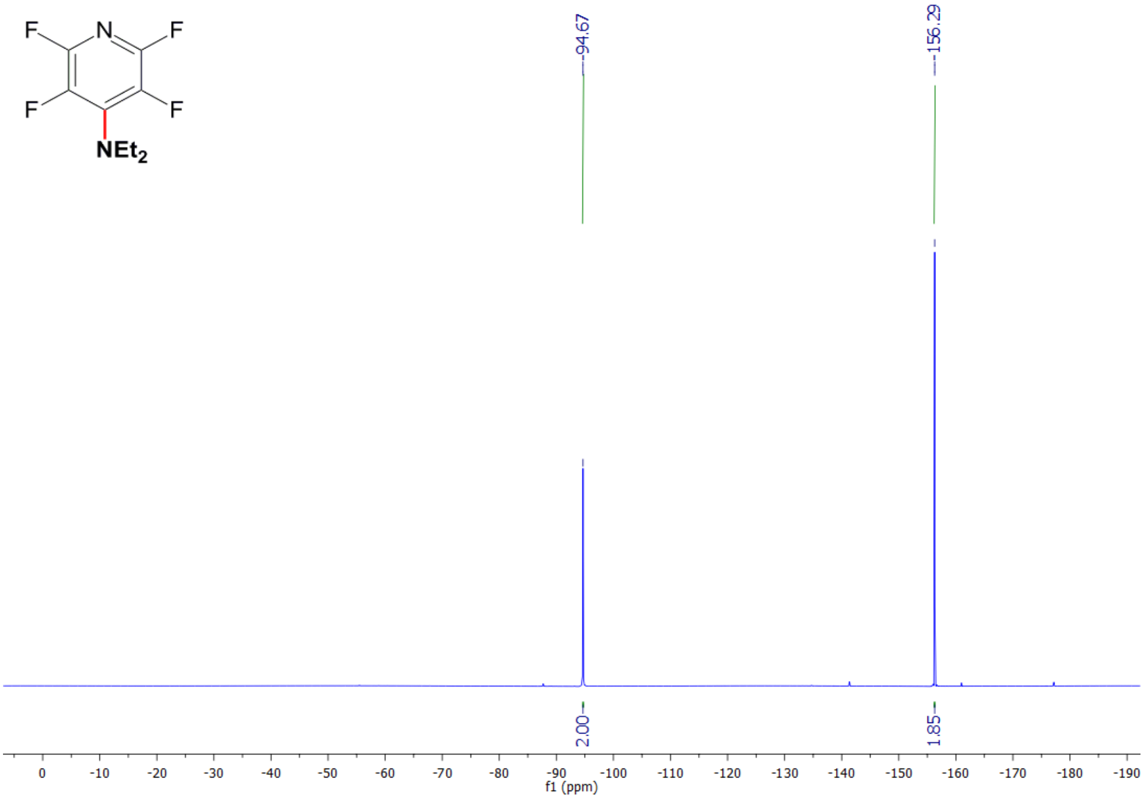

Figure S38. ^{19}F NMR spectrum (376.5 MHz, $CDCl_3$) of **14**.

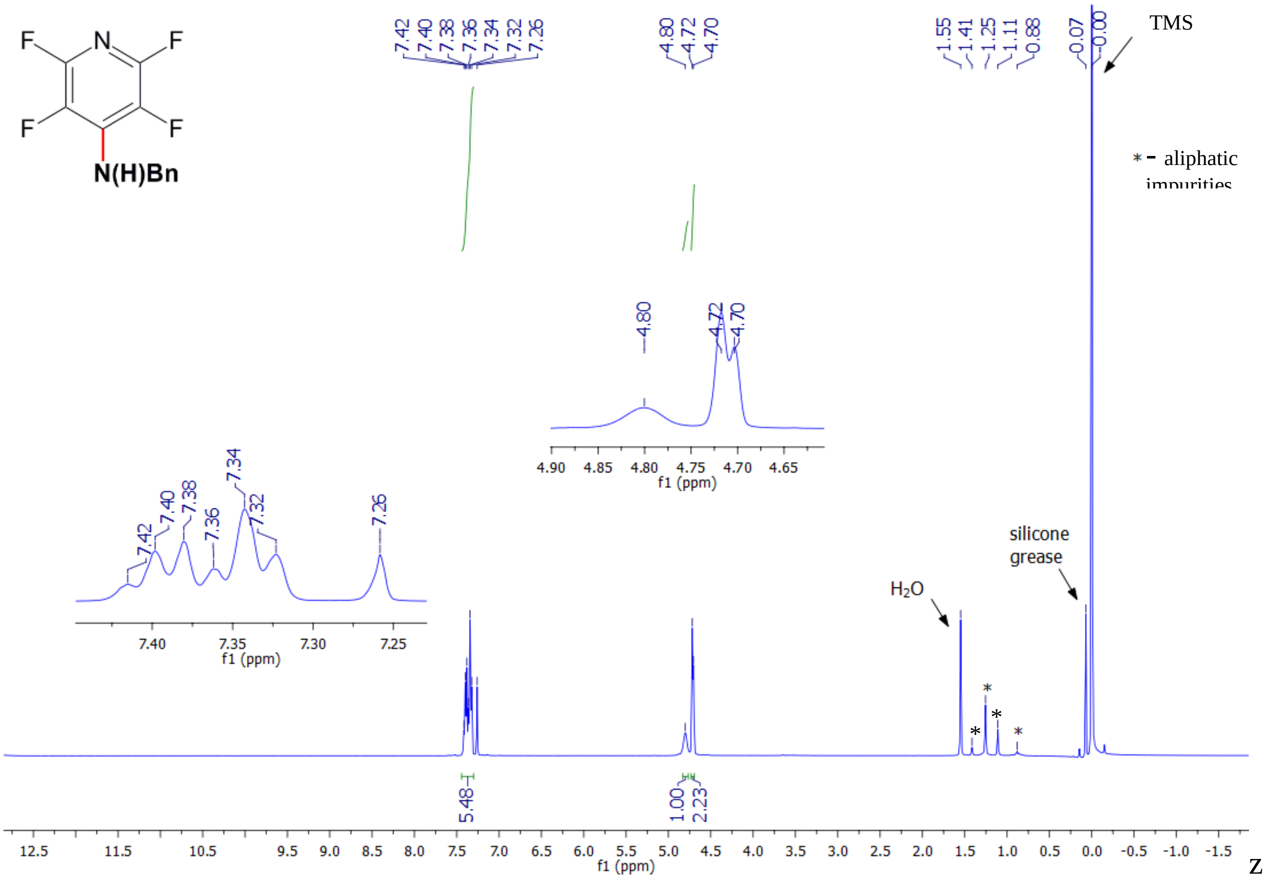

Figure S39. ^{1}H NMR spectrum (400 MHz, $CDCl_3$) of **14a**.

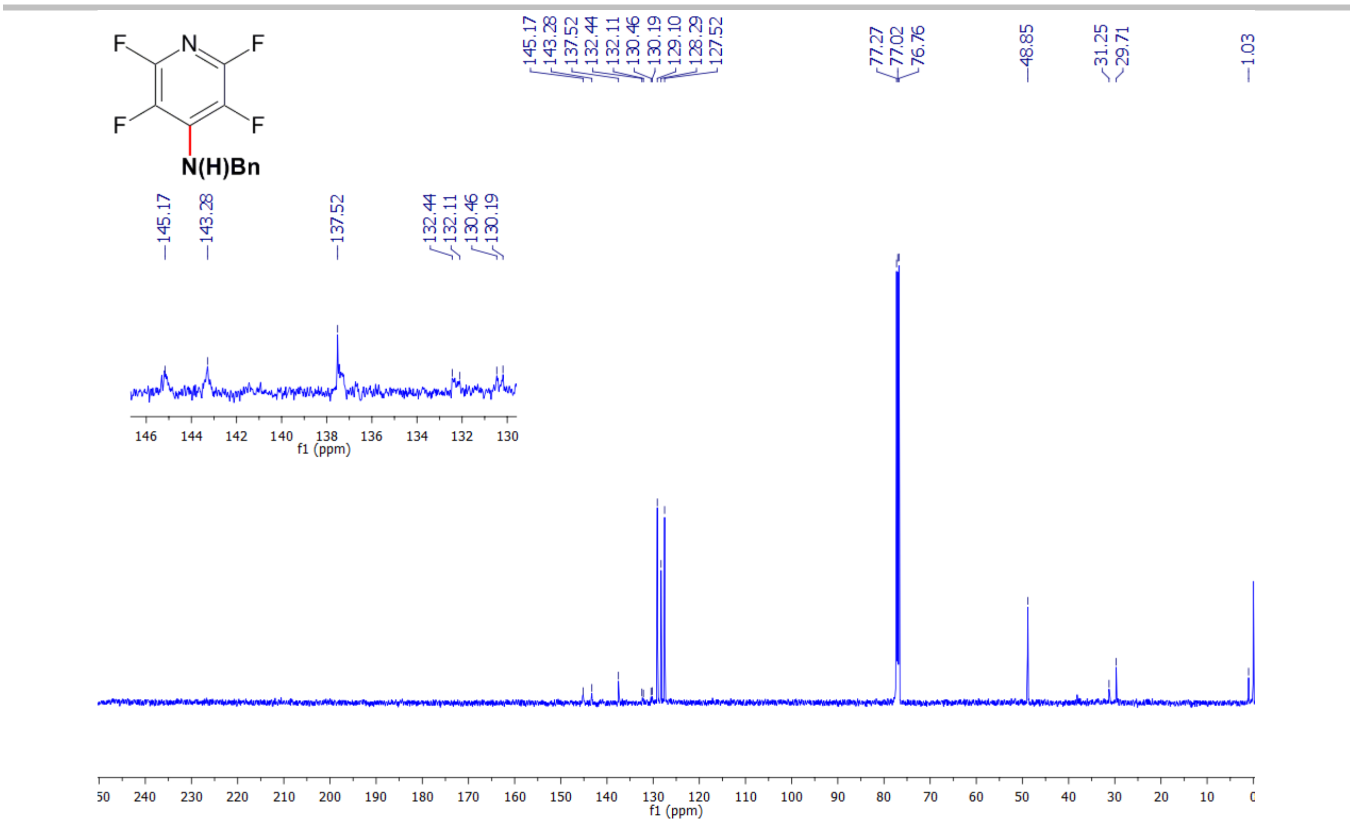

Figure S40. ^{13}C NMR spectrum (100 MHz, $CDCl_3$) of **14a**.

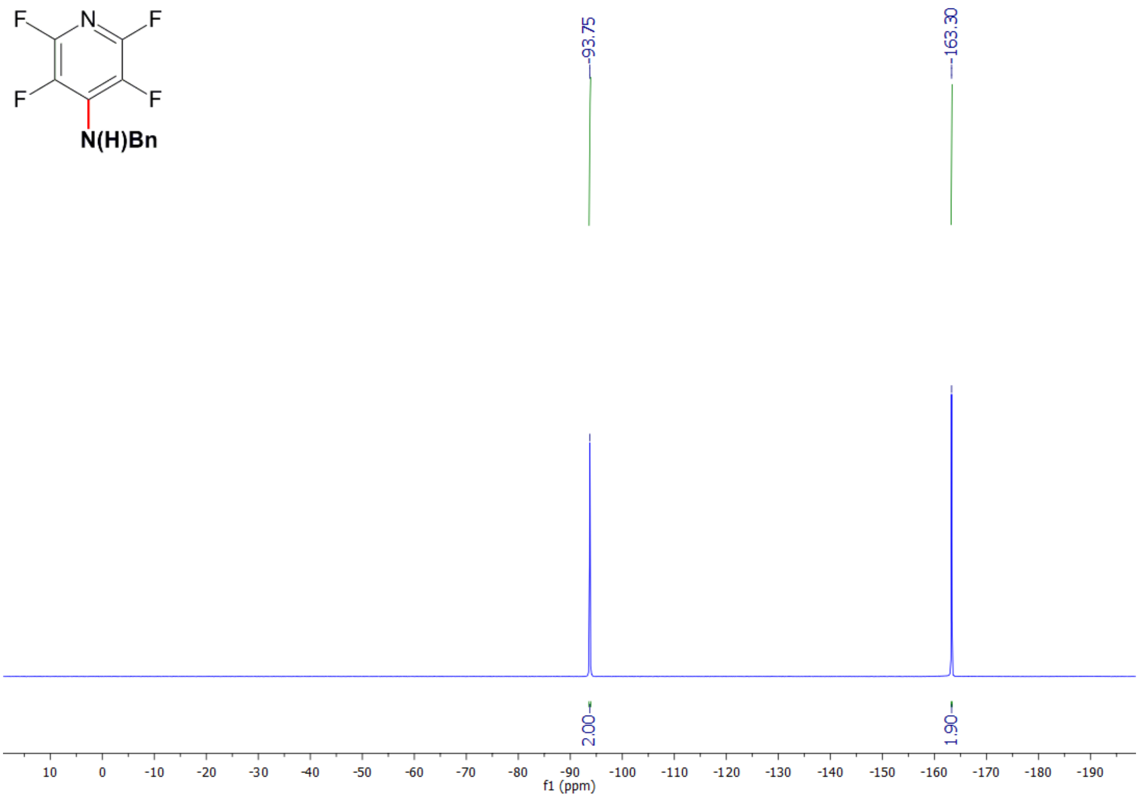

Figure S41. ^{19}F NMR spectrum (376.5 MHz, $CDCl_3$) of **14a**.

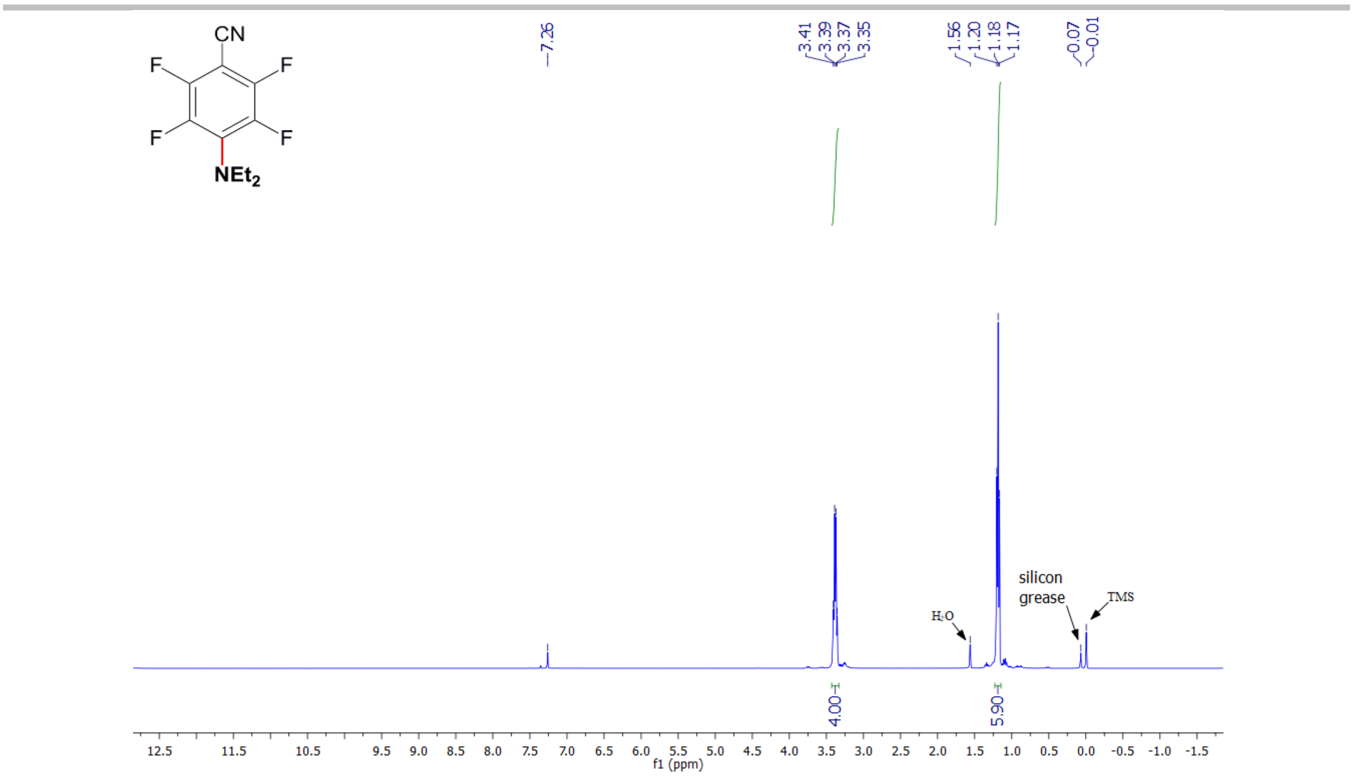

Figure S42. ^{1}H NMR spectrum (400 MHz, $CDCl_3$) of **15**.

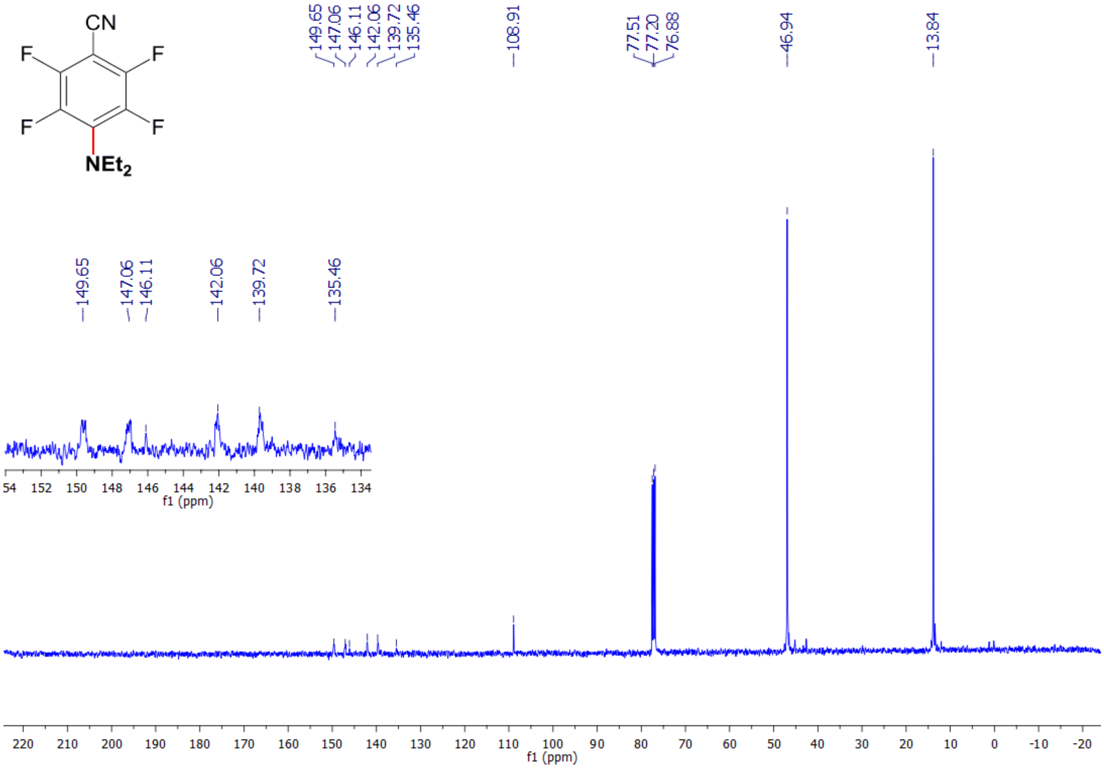

Figure S43. ^{13}C NMR spectrum (100 MHz, $CDCl_3$) of **15**.

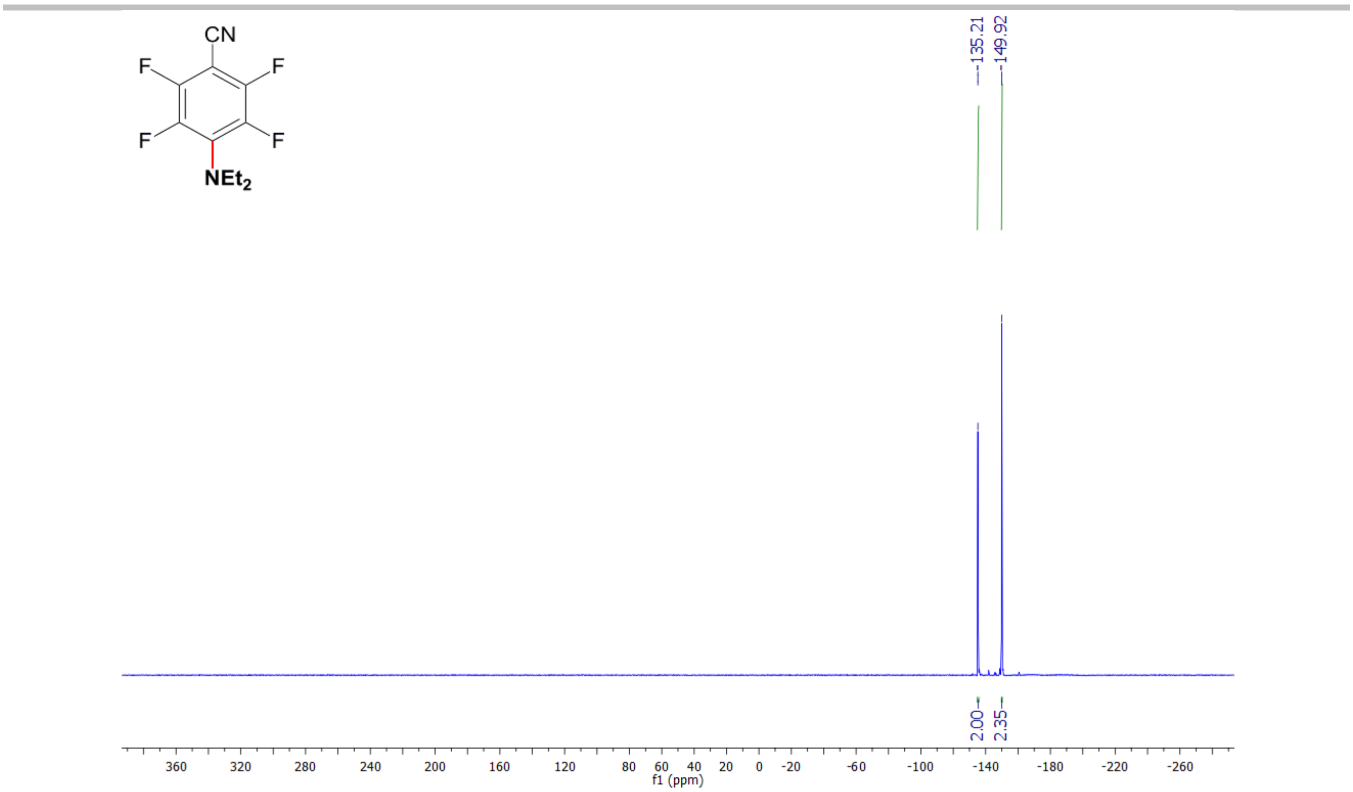

Figure S44. ^{19}F NMR spectrum (376.5 MHz, $CDCl_3$) of **15**.

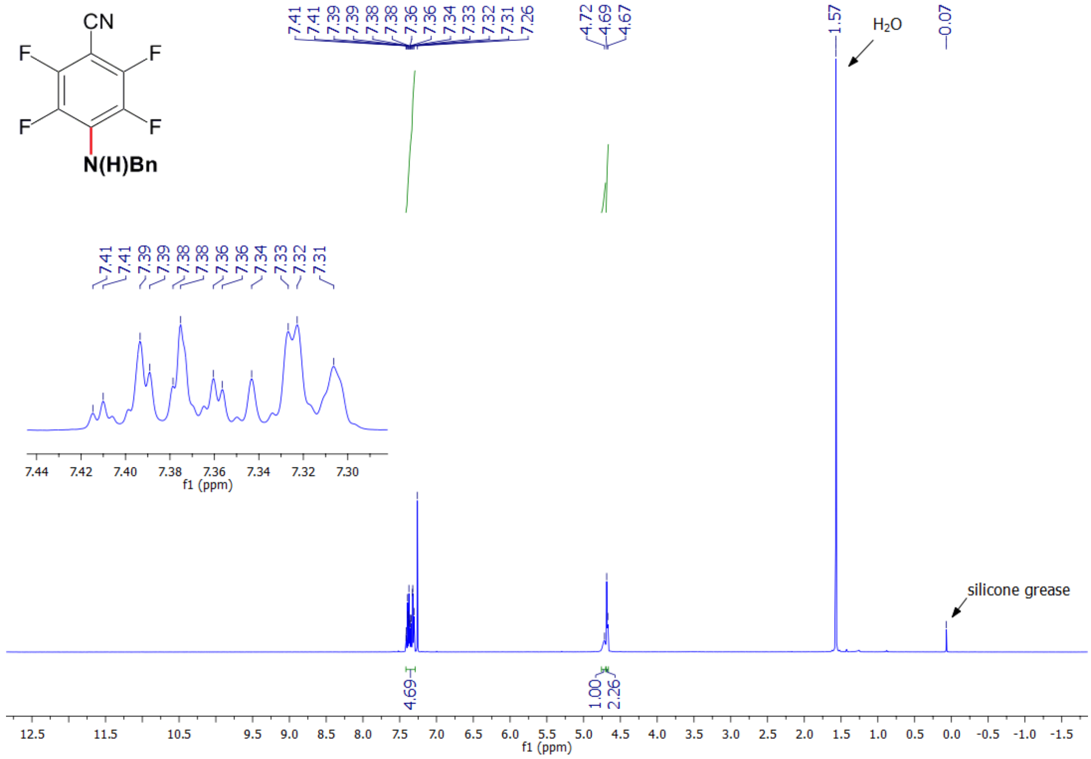

Figure S45. ^{1}H NMR spectrum (400 MHz, $CDCl_3$) of **15a**.

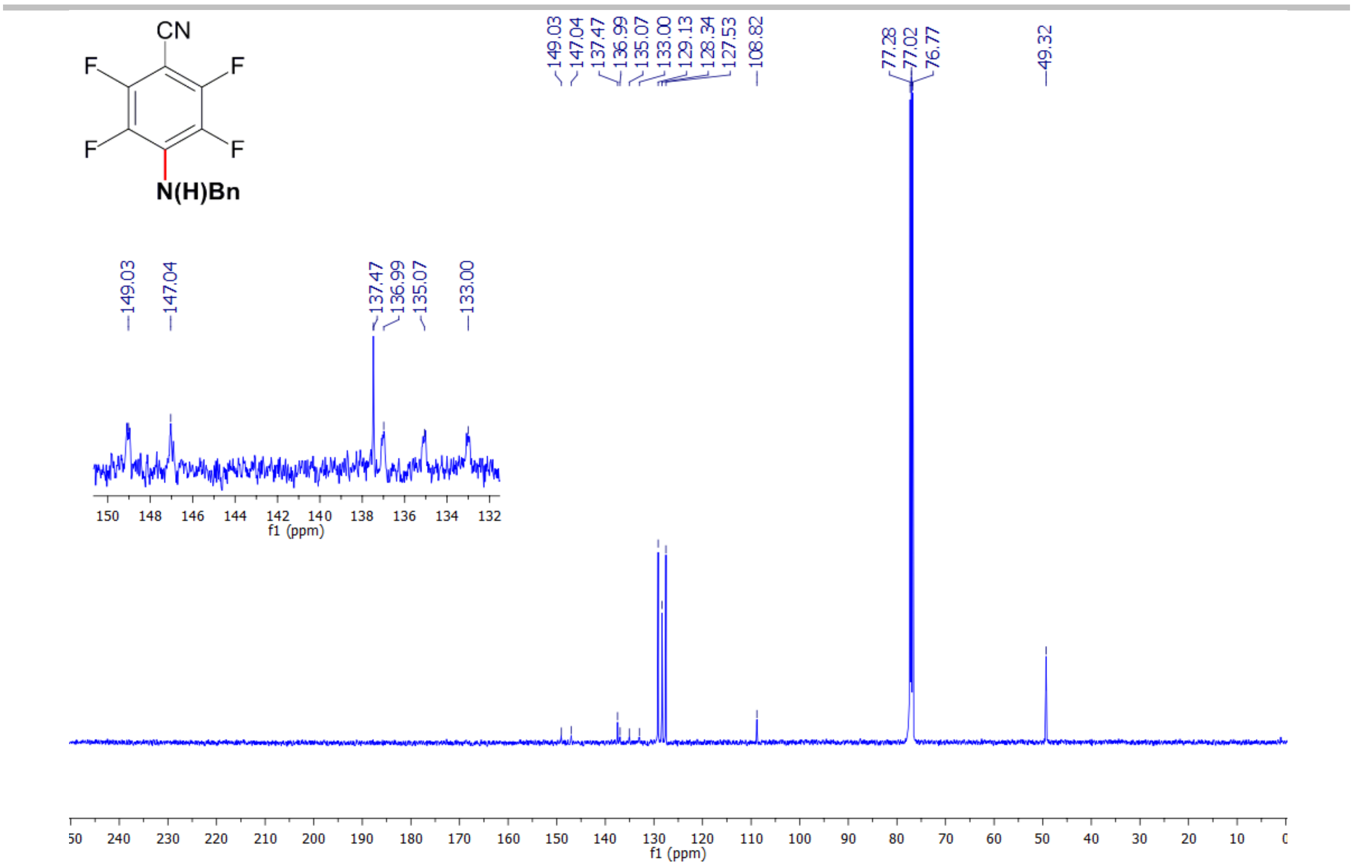

Figure S46. ^{13}C NMR spectrum (100 MHz, $CDCl_3$) of **15a**.

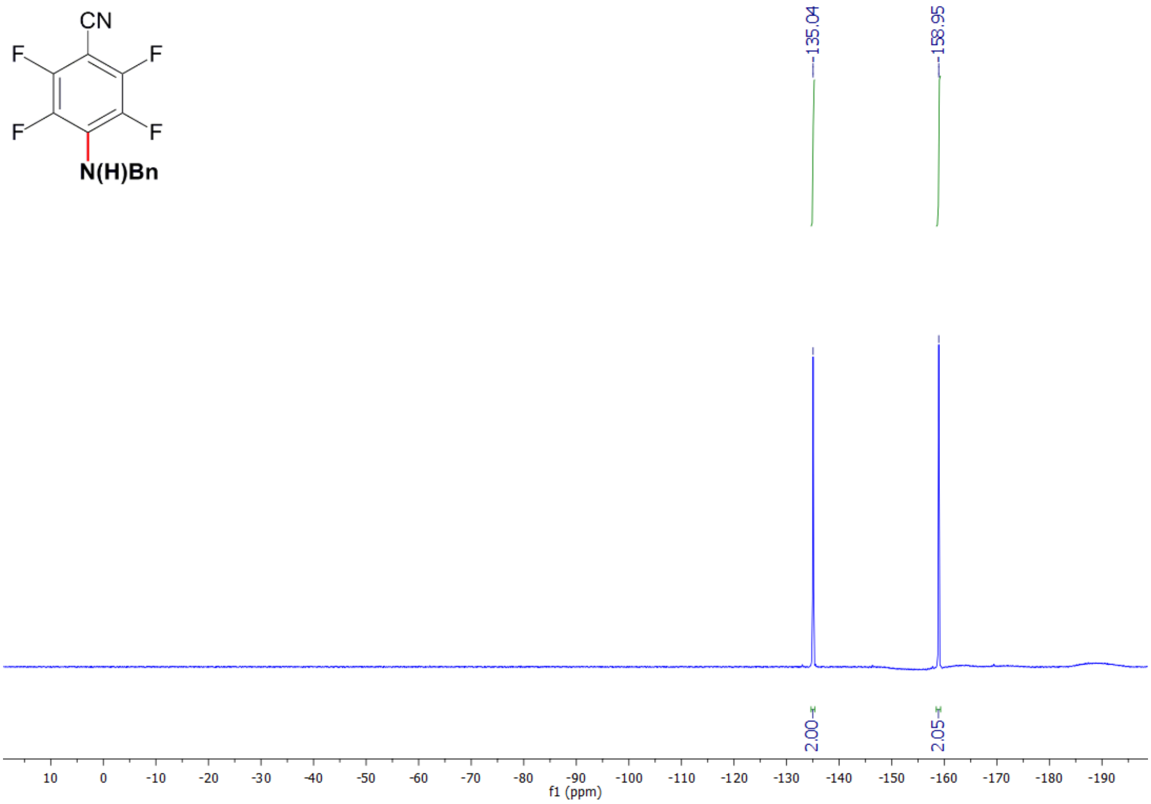

Figure S47. ^{19}F NMR spectrum (376.5 MHz, $CDCl_3$) of **15a**.

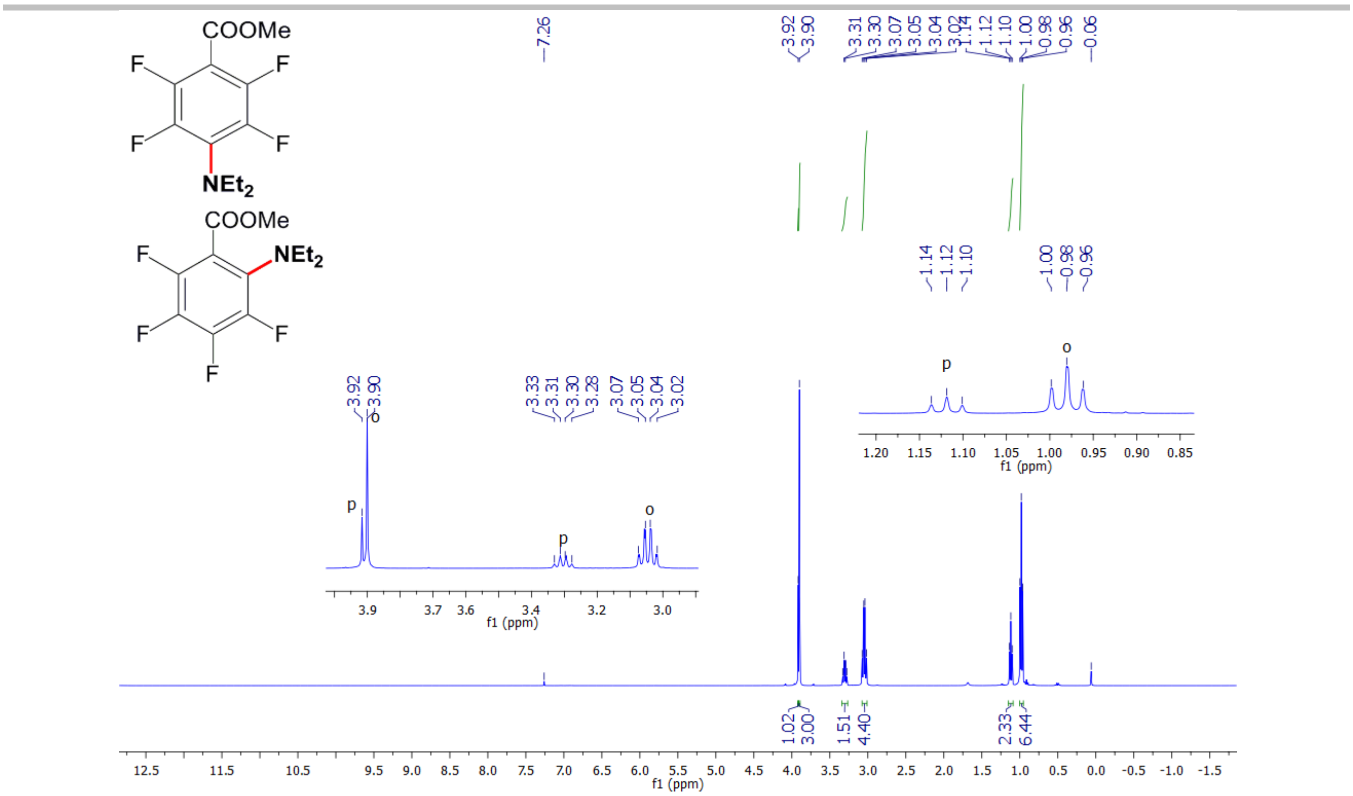

Figure S48. ^{1}H NMR spectrum (400 MHz, $CDCl_3$) of ortho and para isomers of **16** and **17**.

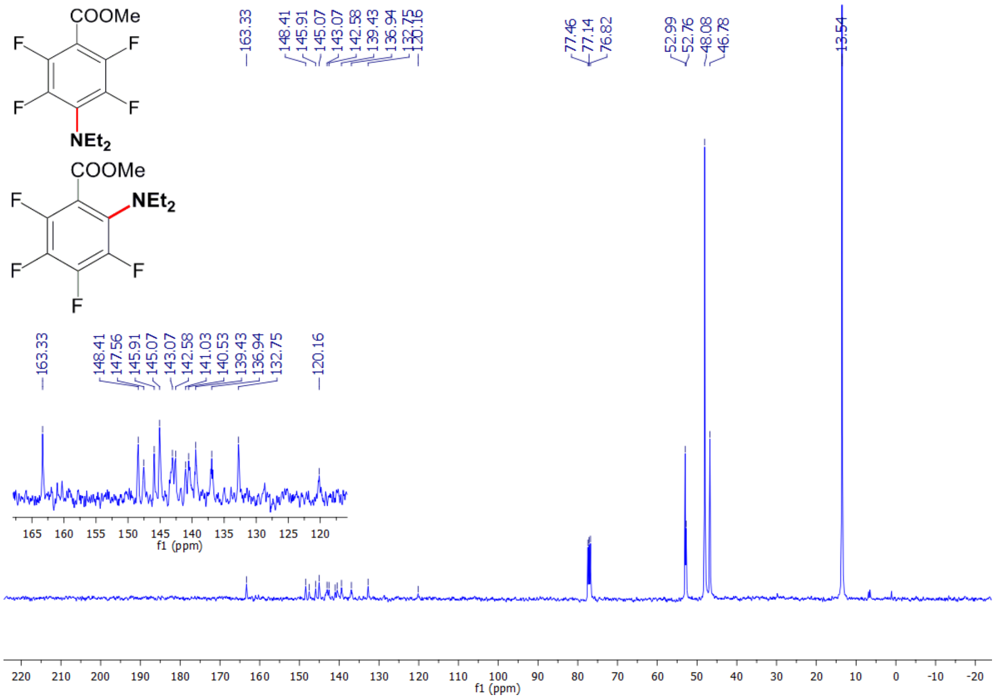

Figure S49. ^{13}C NMR spectrum (100 MHz, $CDCl_3$) of ortho and para isomers of **16** and **17**.

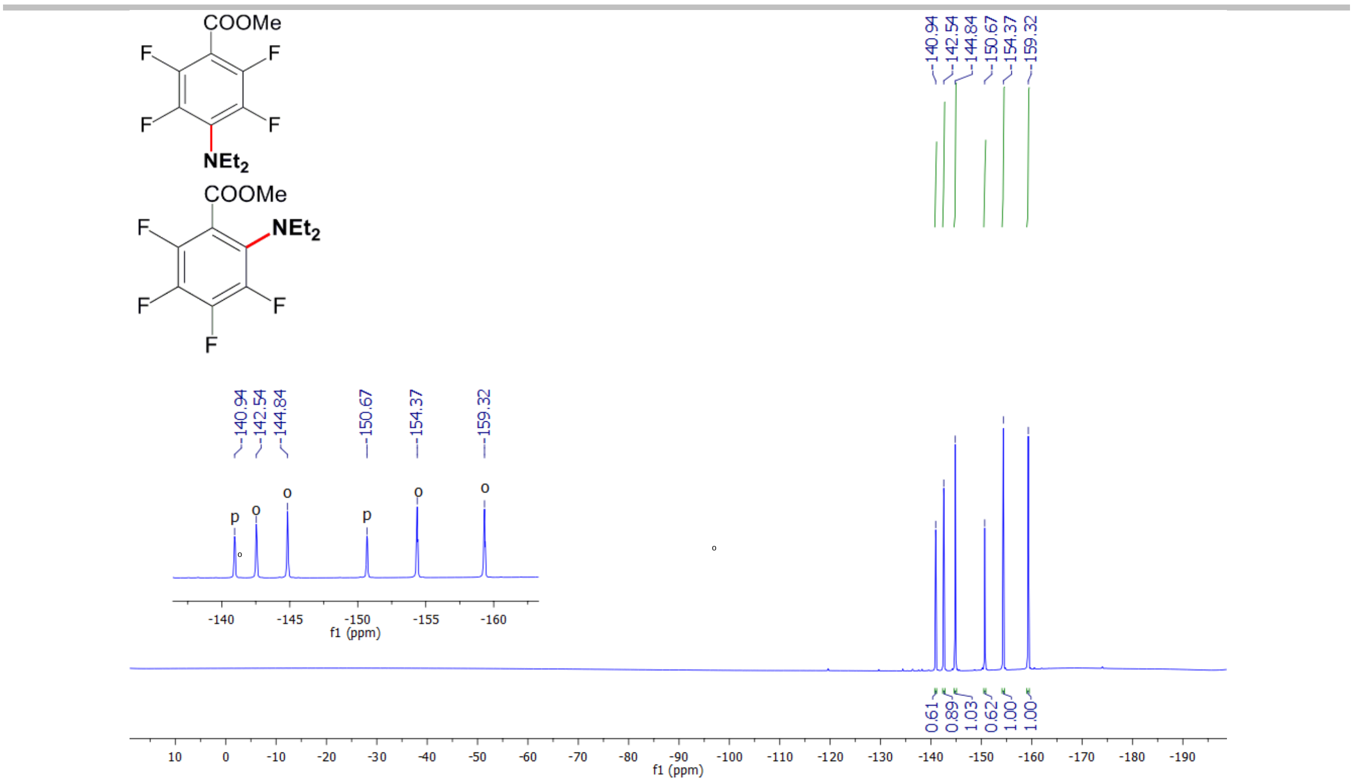

Figure S50. ^{19}F NMR spectrum (376.5 MHz, $CDCl_3$) of ortho and para isomers of **16** and **17**.

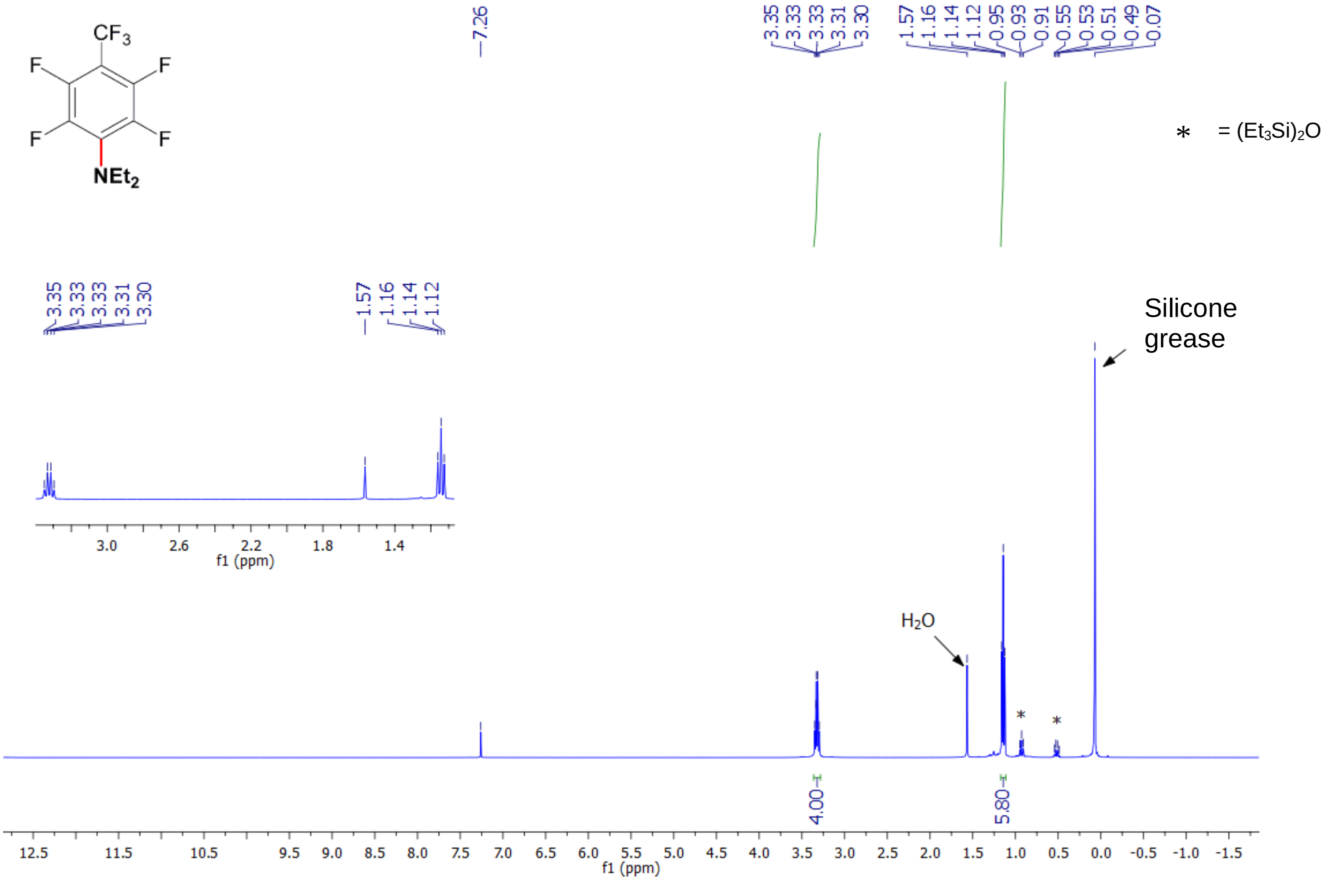

Figure S51. ^{1}H NMR spectrum (400 MHz, $CDCl_3$) of **18**.

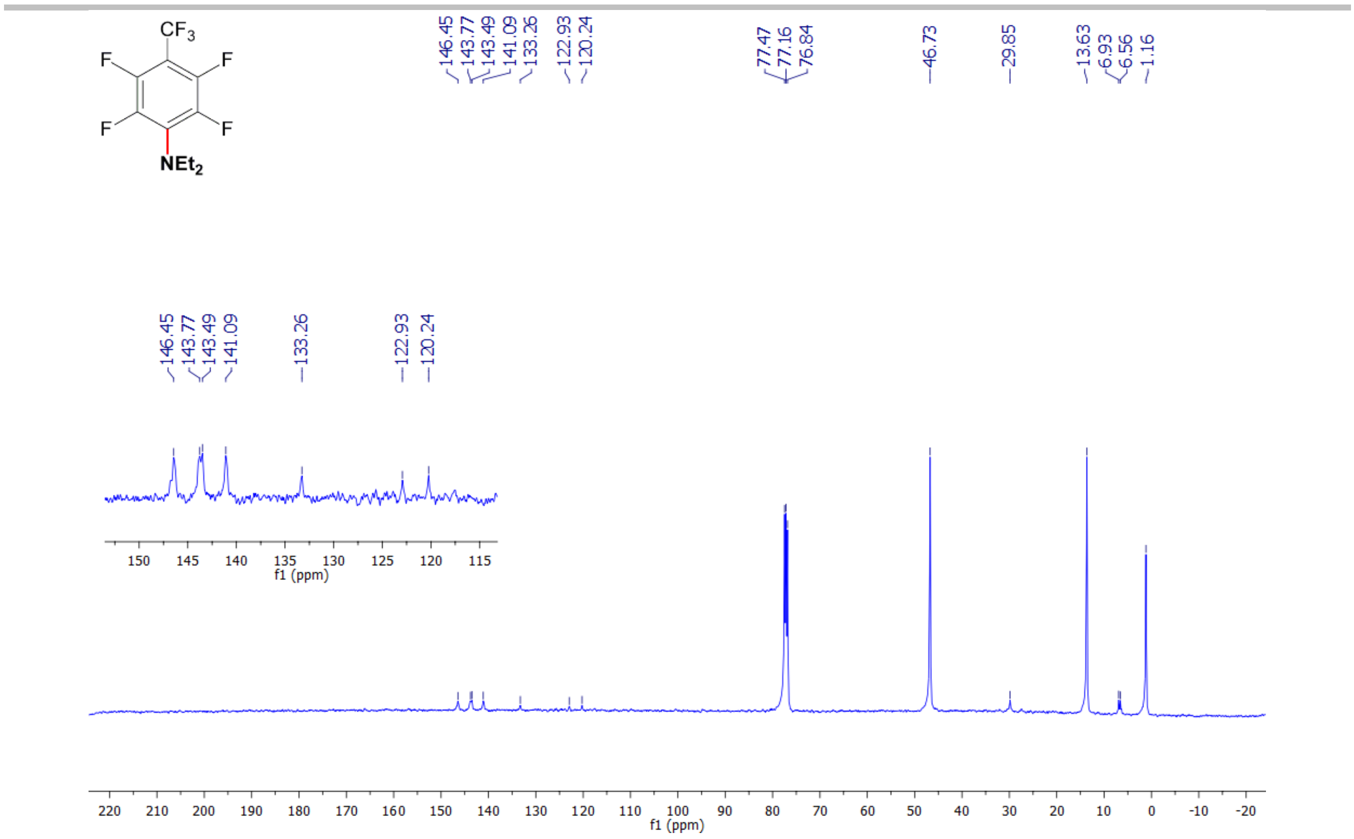

Figure S52. ^{13}C NMR spectrum (100 MHz, $CDCl_3$) of **18**.

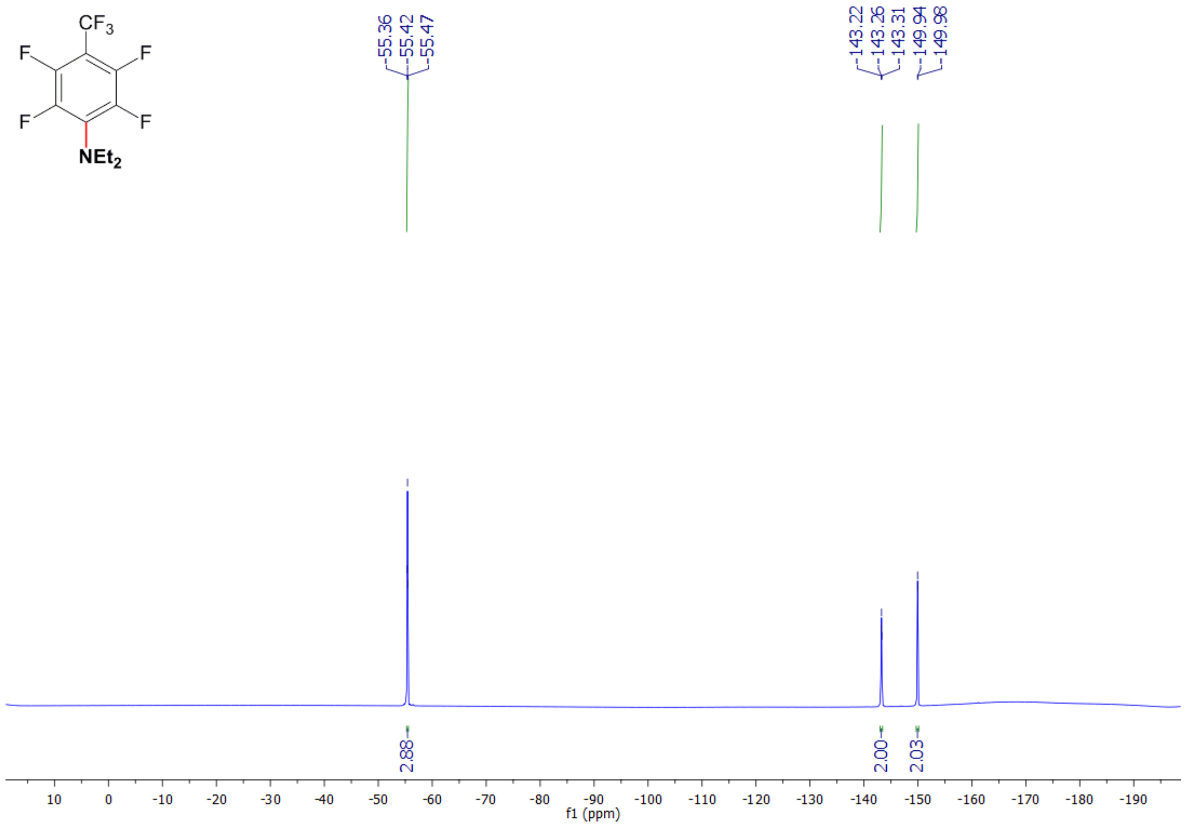

Figure S53. ^{19}F NMR spectrum (376.5 MHz, $CDCl_3$) of **18**.

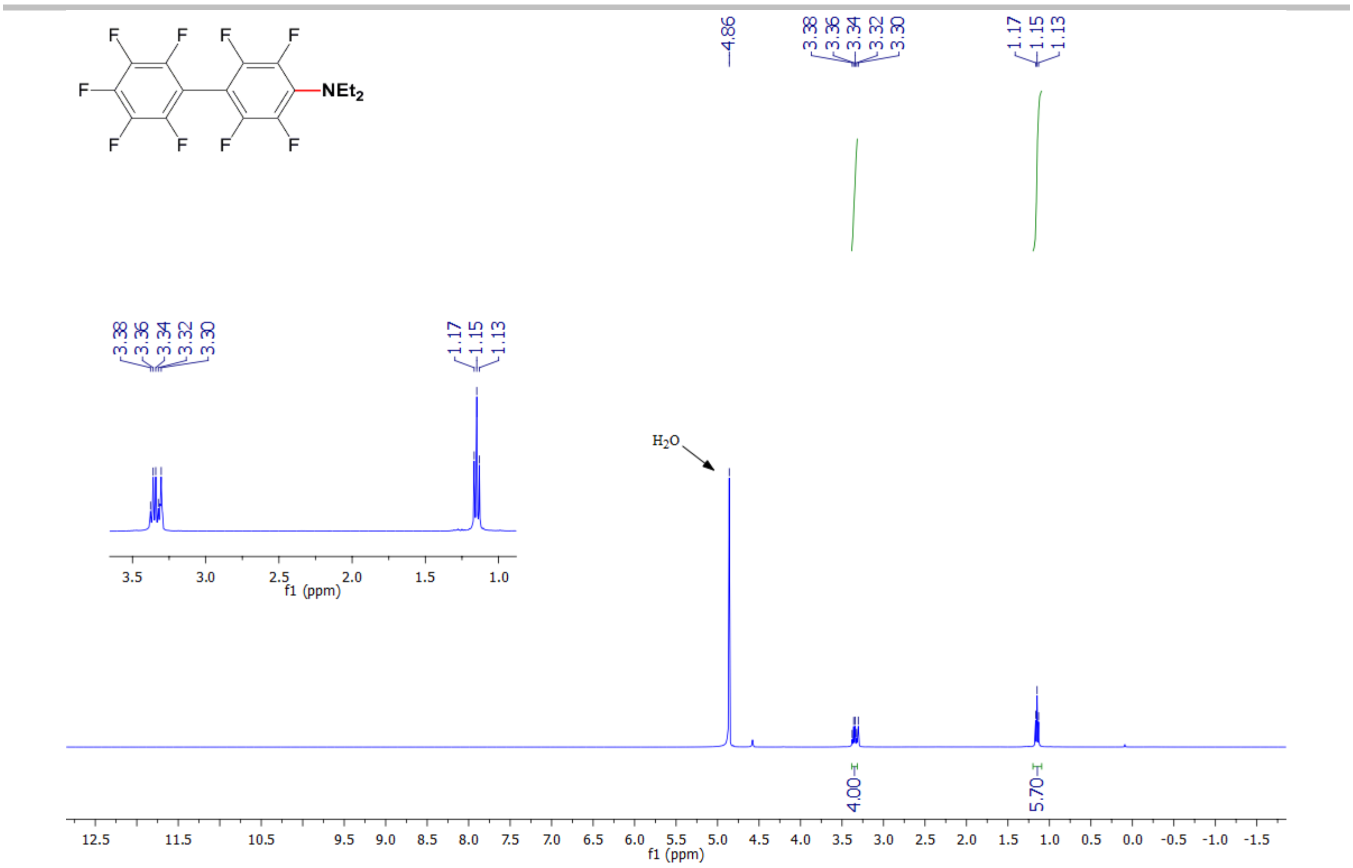

Figure S54. ^{1}H NMR spectrum (400 MHz, $CDCl_3$) of **19**.

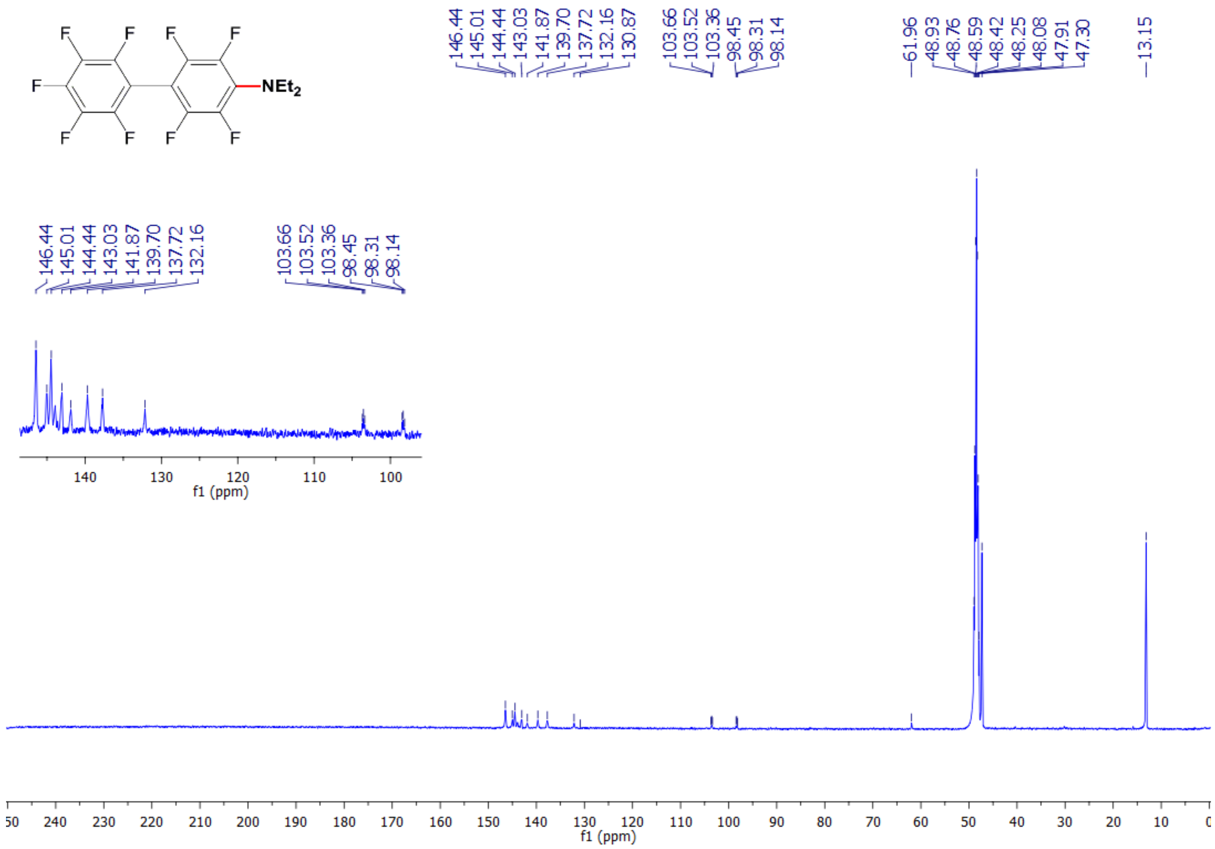

Figure S55. ^{13}C NMR spectrum (100 MHz, $CDCl_3$) of **19**.

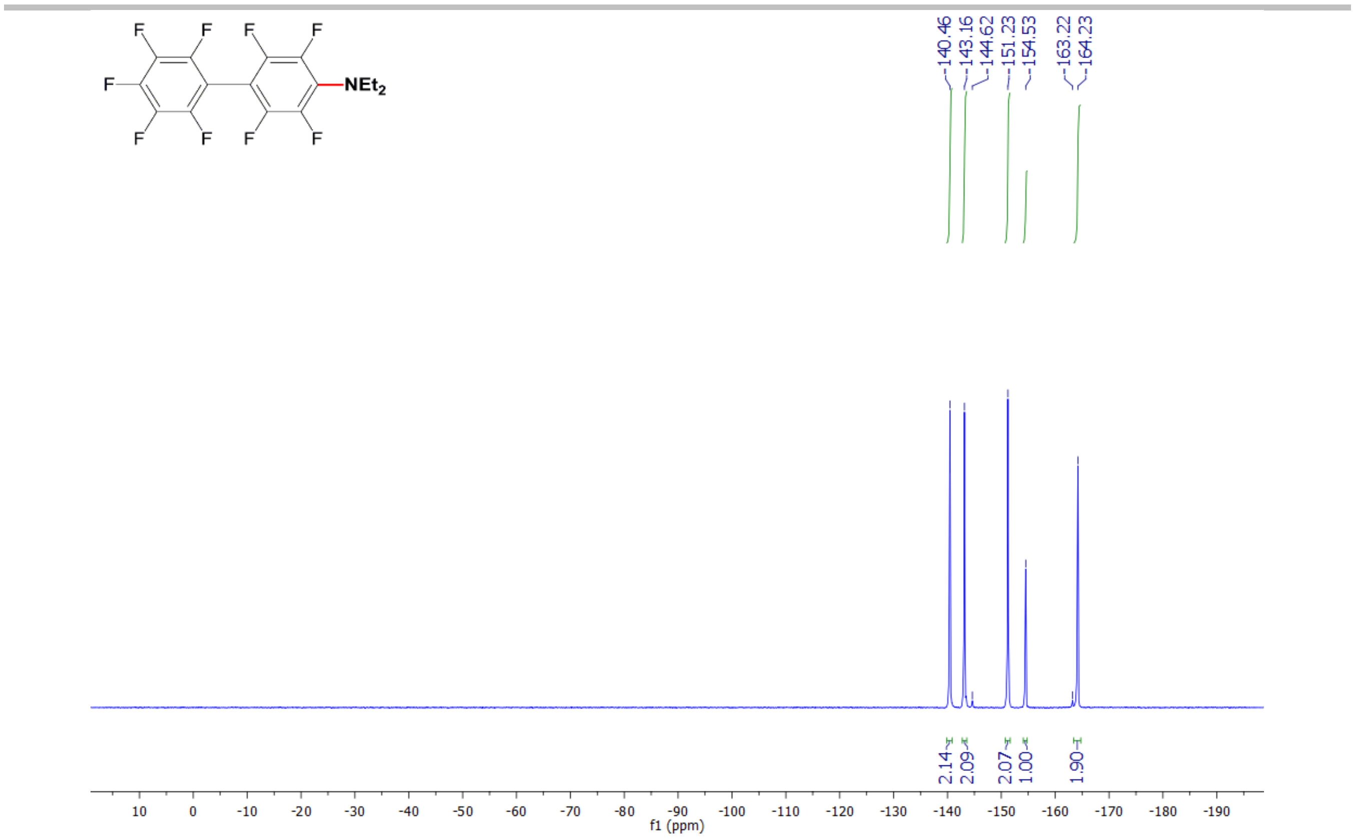

Figure S56. ^{19}F NMR spectrum (375.6 MHz, $CDCl_3$) of **19**.

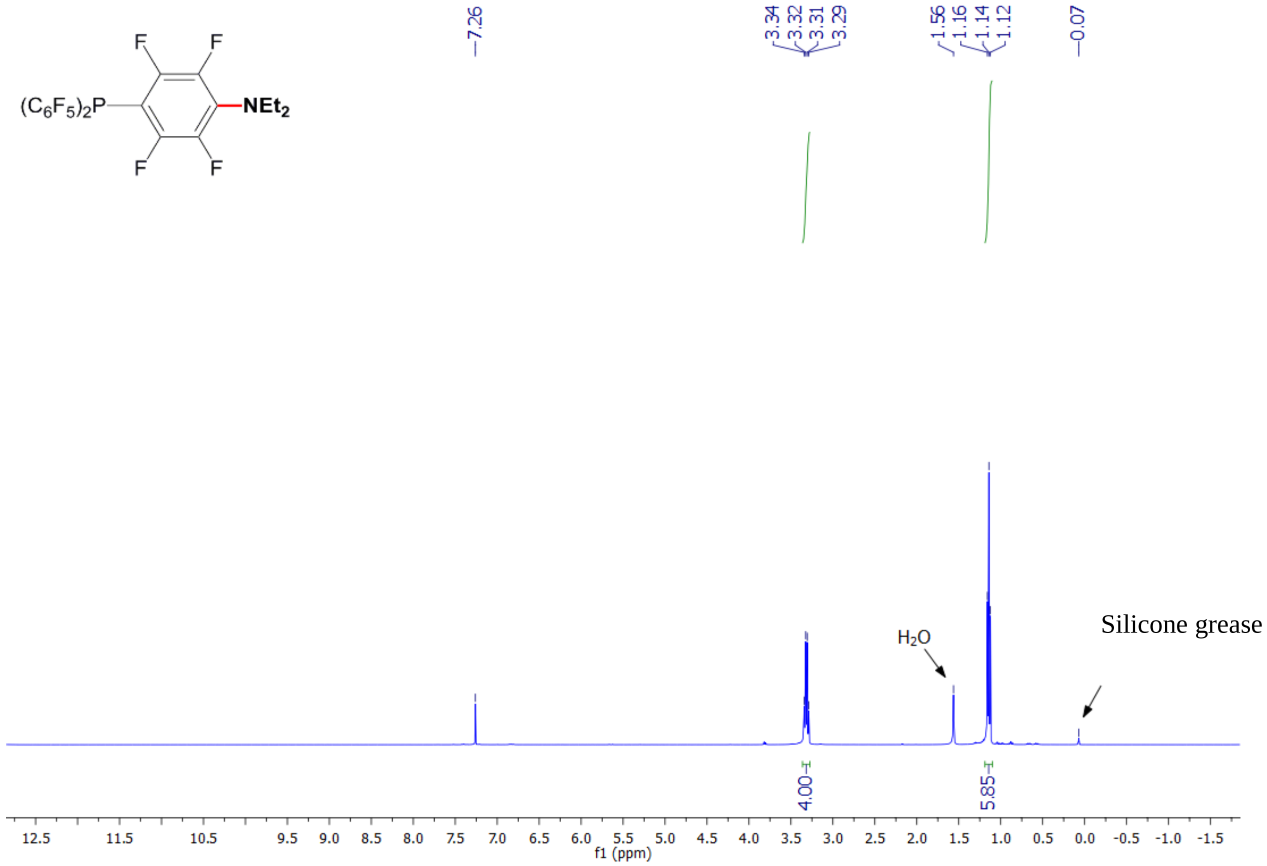

Figure S57. ^{1}H NMR spectrum (400 MHz, $CDCl_3$) of **20**.

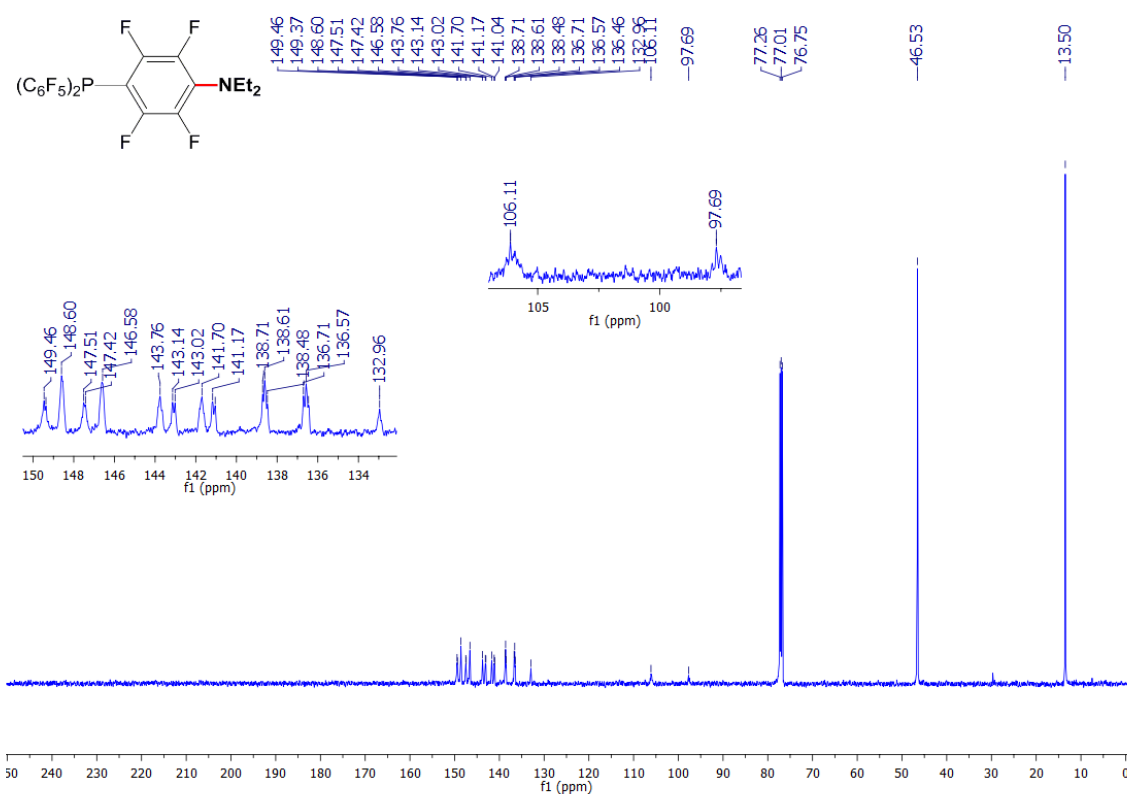

Figure S58. ^{13}C NMR spectrum (100 MHz, $CDCl_3$) of **20**.

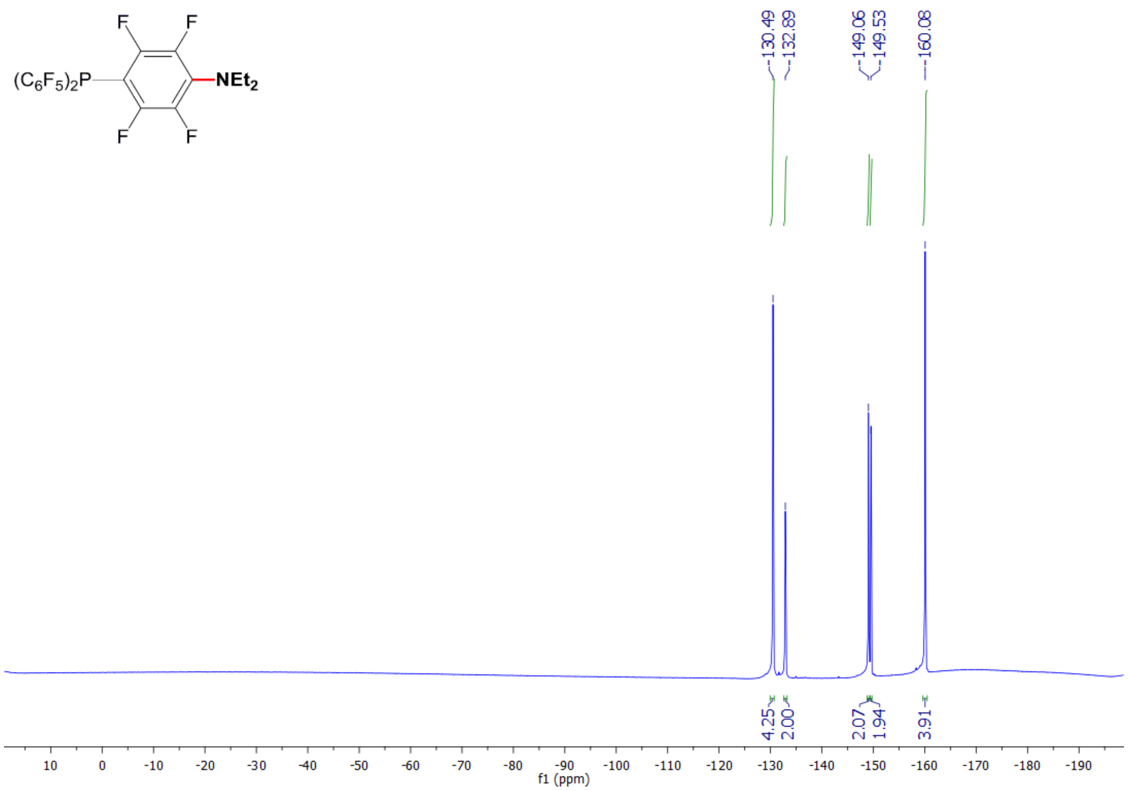

Figure S59. ^{19}F NMR spectrum (376.5 MHz, $CDCl_3$) of **20**.

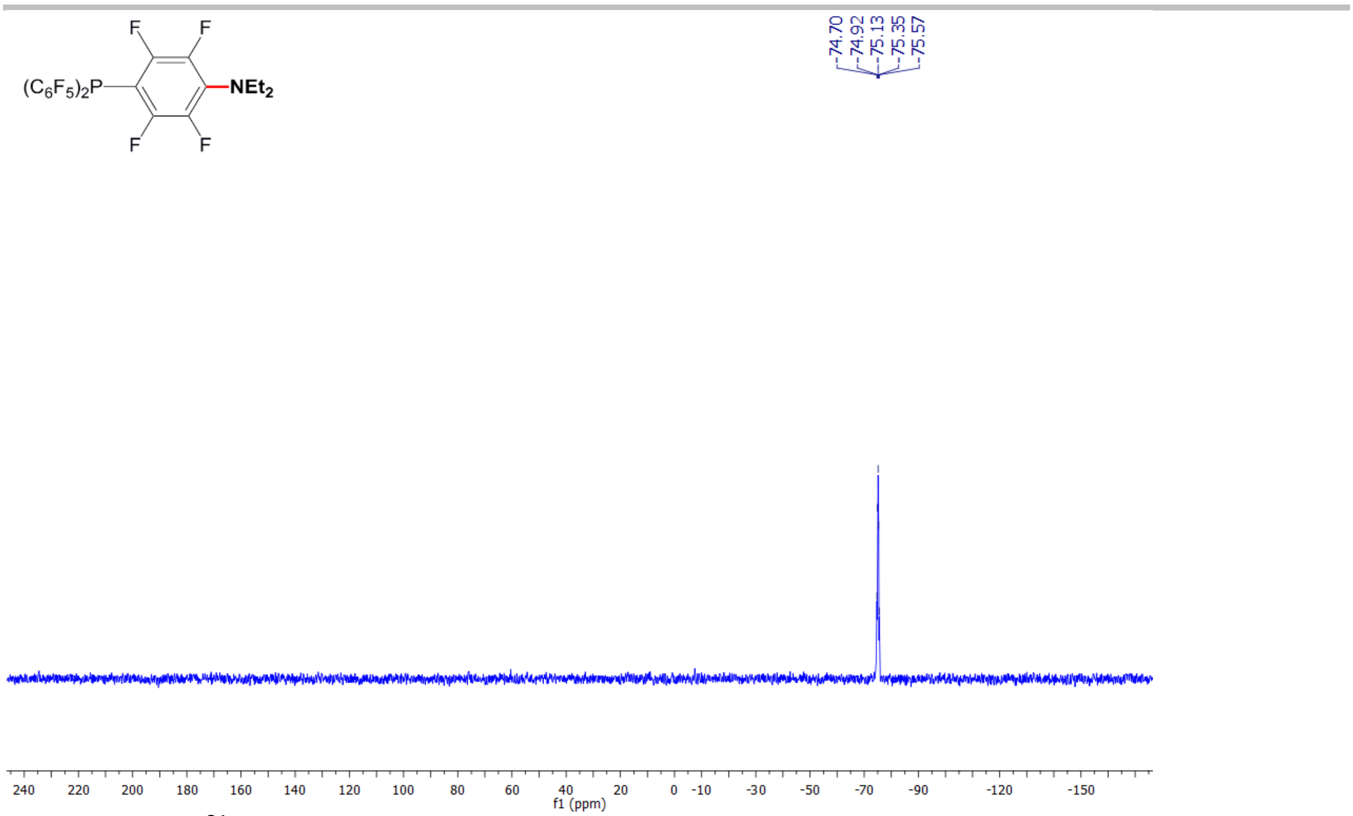

Figure S60. ^{31}P NMR spectrum (162 MHz, $CDCl_3$) of **20**.

4. DFT calculations

DFT calculations were performed using Gaussian 09.2 Geometry optimization of all the molecules, intermediates, and the transition state were carried out using the BP86(D3)/def2-SVP or BP86(D3)/def2-TZVP basis sets implemented in the Gaussian 09 software.[14-16] Thermal energy corrections were extracted from the results of frequency analysis performed at the same level of theory. Frequency analysis of all the molecules and intermediates contained no imaginary frequency showing that these are energy minima. The transition states geometries gave one imaginary frequency at expected reaction coordinates confirming that it is a first-order saddle point.

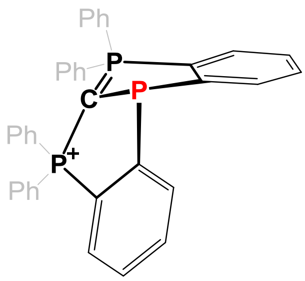

(1$^+$)

(*BP86(D3)/def2-TZVP*):

C	0.05436	0.48748	-0.75896
P	-1.53610	0.00565	-0.30736
P	1.56558	-0.03682	-0.11750
C	-2.32844	1.62531	-0.15768
C	-3.66061	1.83177	0.22623
C	-1.47547	2.71303	-0.43306
C	-4.13462	3.13650	0.36600
H	-4.31825	0.98372	0.42668
C	-1.97484	4.01842	-0.32089
C	-3.29018	4.22429	0.10135
H	-5.16649	3.30969	0.67469
H	-1.34148	4.87387	-0.56540
H	-3.66856	5.24207	0.21101
C	2.14050	1.50471	0.64706
C	1.40567	2.62962	0.21026
C	3.20675	1.64240	1.54540
C	1.76133	3.89953	0.68236
C	3.52027	2.90937	2.04087
H	3.77610	0.76870	1.86970
C	2.79552	4.03066	1.61329
H	1.22675	4.78692	0.33635
H	4.33560	3.02700	2.75610
H	3.04645	5.01803	2.00485

C	-2.39359	-0.98916	-1.55835
C	-3.65147	-1.56045	-1.29290
C	-1.81795	-1.12092	-2.83048
C	-4.32500	-2.25186	-2.30019
H	-4.09653	-1.48006	-0.29901
C	-2.49563	-1.81859	-3.83311
H	-0.83842	-0.67783	-3.02126
C	-3.74841	-2.38042	-3.56976
H	-5.30040	-2.69564	-2.09442
H	-2.04446	-1.92389	-4.82107
H	-4.27752	-2.92454	-4.35394
C	-1.75358	-0.87178	1.26664
C	-1.88606	-0.12592	2.45074
C	-1.74919	-2.27541	1.31750
C	-2.04027	-0.78287	3.67205
H	-1.88187	0.96492	2.41329
C	-1.88822	-2.92436	2.54532
H	-1.64248	-2.85790	0.40124
C	-2.04436	-2.18087	3.71940
H	-2.16251	-0.20224	4.58784
H	-1.87730	-4.01448	2.58389
H	-2.16707	-2.69320	4.67516
C	2.76228	-0.50038	-1.40014
C	2.44096	-0.31374	-2.75209
C	4.03260	-0.98203	-1.03709
C	3.38553	-0.61114	-3.73823
H	1.45405	0.06577	-3.02364
C	4.96974	-1.27878	-2.02630
H	4.28415	-1.13789	0.01426
C	4.64675	-1.09279	-3.37643
H	3.13509	-0.46618	-4.79029
H	5.95405	-1.65706	-1.74588
H	5.38267	-1.32601	-4.14775
C	1.54600	-1.39438	1.07130
C	1.37800	-1.12954	2.44033
C	1.68165	-2.71798	0.61791
C	1.36387	-2.18621	3.35044
H	1.25969	-0.10256	2.78889
C	1.65816	-3.76925	1.53577
H	1.82173	-2.92252	-0.44503
C	1.50653	-3.50314	2.90011
H	1.23565	-1.98104	4.41391
H	1.76792	-4.79673	1.18548
H	1.49857	-4.32603	3.61692

P	0.17141	2.28438	-1.12584

Sum of electronic and zero-point Energies= -2450.857814
Sum of electronic and thermal Energies= -2450.823779
Sum of electronic and thermal Enthalpies= -2450.822835
Sum of electronic and thermal Free Energies= -2450.924188

(*BP86(D3)/def2-SVP*):

P	1.56208	-0.05822	0.09775
P	0.23324	2.19157	1.38888
P	-1.52787	-0.01238	0.37707
C	0.08123	0.41638	0.87834
C	2.09880	1.55197	-0.58320
C	3.11522	1.76205	-1.53524
H	3.67139	0.91099	-1.95963
C	3.40010	3.07288	-1.95170
H	4.17832	3.25071	-2.70965
C	2.69874	4.16021	-1.39314
H	2.92785	5.18519	-1.72431
C	1.71761	3.95228	-0.40766
H	1.20093	4.81589	0.04147
C	1.39042	2.64156	-0.01038
C	-1.45656	2.68025	0.81231
C	-1.95550	3.99821	0.84894
H	-1.30229	4.83146	1.15436
C	-3.29266	4.24855	0.49772
H	-3.67320	5.28181	0.50587
C	-4.15548	3.18834	0.15362
H	-5.20613	3.39435	-0.10191
C	-3.68037	1.86765	0.14216
H	-4.35433	1.03904	-0.12568
C	-2.33061	1.62166	0.46160
C	1.45476	-1.33735	-1.18604
C	1.31064	-0.98721	-2.54591
H	1.25501	0.06996	-2.84616
C	1.23219	-2.00011	-3.51375
H	1.12330	-1.73020	-4.57497
C	1.28129	-3.35236	-3.12943
H	1.22133	-4.14205	-3.89443
C	1.40416	-3.69966	-1.77212
H	1.43996	-4.75828	-1.47204
C	1.49661	-2.69510	-0.79602
H	1.61854	-2.96340	0.26539
C	2.83225	-0.62007	1.28308
C	4.07091	-1.11030	0.81123

H	4.25526	-1.21456	-0.27041
C	5.06450	-1.48107	1.72979
H	6.02912	-1.86821	1.36669
C	4.82695	-1.36042	3.11269
H	5.60979	-1.65184	3.83015
C	3.59410	-0.87223	3.57993
H	3.40989	-0.77963	4.66141
C	2.59199	-0.50056	2.66723
H	1.62204	-0.11492	3.02032
C	-1.76925	-0.63231	-1.32646
C	-1.80331	0.30888	-2.38056
H	-1.71038	1.38472	-2.16231
C	-1.96980	-0.13220	-3.70210
H	-2.01404	0.60148	-4.52188
C	-2.08751	-1.50736	-3.97607
H	-2.22280	-1.85108	-5.01341
C	-2.02828	-2.44438	-2.92938
H	-2.10451	-3.52086	-3.14575
C	-1.87138	-2.01258	-1.60235
H	-1.83001	-2.74834	-0.78481
C	-2.34346	-1.21175	1.48400
C	-1.67809	-1.59851	2.66520
H	-0.67144	-1.19815	2.86723
C	-2.30213	-2.48663	3.55725
H	-1.78240	-2.79309	4.47822
C	-3.58782	-2.98065	3.27453
H	-4.07650	-3.67469	3.97604
C	-4.25276	-2.59422	2.09489
H	-5.25759	-2.98603	1.87364
C	-3.63407	-1.71287	1.19497
H	-4.14942	-1.43054	0.26304

Sum of electronic and zero-point Energies= -2448.982637
Sum of electronic and thermal Energies= -2448.948673
Sum of electronic and thermal Enthalpies= -2448.947729
Sum of electronic and thermal Free Energies= -2449.048981

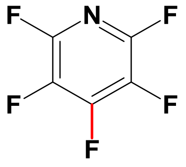

(**3**)

(*BP86(D3)/def2-TZVP*):

C	-0.00000	-1.12830	0.00000
C	1.20938	-0.42279	-0.00001
C	-1.20938	-0.42280	0.00000

C	1.13522	0.97271	-0.00002
C	-1.13522	0.97271	0.00002
N	-0.00000	1.64147	-0.00000
F	2.37823	-1.07821	0.00000
F	2.27302	1.68036	0.00001
F	-2.27302	1.68036	-0.00000
F	-2.37824	-1.07820	-0.00000
F	0.00001	-2.46202	0.00000

Sum of electronic and zero-point Energies= -744.736117
Sum of electronic and thermal Energies= -744.727514
Sum of electronic and thermal Enthalpies= -744.726570
Sum of electronic and thermal Free Energies= -744.770121

(*BP86(D3)/def2-SVP*):

C	-0.00000	1.13775	-0.00000
C	1.21731	0.42677	0.00001
C	-1.21731	0.42679	-0.00001
C	1.14207	-0.97983	0.00003
C	-1.14206	-0.97982	-0.00002
N	-0.00001	-1.64385	-0.00000
F	2.38333	1.07741	-0.00000
F	2.27753	-1.68235	-0.00001
F	-2.27755	-1.68231	0.00001
F	-2.38335	1.07739	0.00000
F	0.00003	2.46732	0.00000

Sum of electronic and zero-point Energies= -743.858087
Sum of electronic and thermal Energies= -743.849437
Sum of electronic and thermal Enthalpies= -743.848493
Sum of electronic and thermal Free Energies= -743.892139

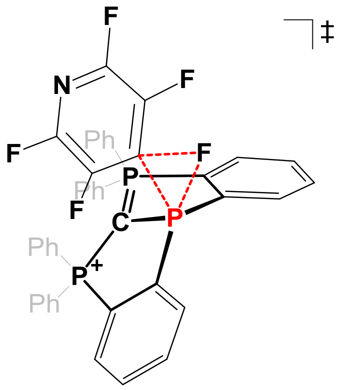

(**TS1**)

(*BP86(D3)/def2-TZVP*):

C	-0.34637	-3.51076	2.04439
C	-0.83690	-3.76581	3.32705
C	-1.33341	-2.72018	4.11863
C	-1.34884	-1.40891	3.63997
C	-0.82213	-1.13984	2.37134
C	-0.33205	-2.19518	1.56733
P	0.25326	-1.67425	-0.07305
C	-0.86038	-2.28017	-1.35743

C	0.19384	0.04950	0.13719
C	1.87441	-2.40083	-0.39229
C	-1.03354	-3.66337	-1.55334
C	-1.91790	-4.11391	-2.53179
C	-2.62662	-3.19380	-3.31570
C	-2.45173	-1.82164	-3.12439
C	-1.56915	-1.36225	-2.14404
C	2.79047	-2.52477	0.66696
C	4.04560	-3.08211	0.43111
C	4.39111	-3.51444	-0.85486
C	3.48454	-3.38348	-1.91041
C	2.22425	-2.82557	-1.68487
P	-0.88072	0.43982	1.49295
C	0.02777	1.82532	2.21196
C	-2.93923	0.92275	0.96518
F	-3.18398	1.33903	2.31937
P	1.39427	1.28116	-0.08735
C	-2.92245	1.95784	-0.02192
C	-3.49678	1.75050	-1.26342
N	-4.13905	0.65257	-1.62918
C	-4.21089	-0.30897	-0.71935
C	-3.66588	-0.23805	0.55092
C	-0.33332	2.55017	3.35509
C	0.37286	3.71153	3.67517
C	1.39665	4.18269	2.84039
C	1.73567	3.48629	1.67804
C	1.06220	2.29854	1.37465
F	-2.23167	3.09414	0.26451
F	-3.39320	2.73051	-2.19105
F	-3.76128	-1.27688	1.41945
F	-4.85923	-1.43329	-1.07761
C	1.19564	2.29387	-1.56879
C	3.11775	0.73350	-0.03894
C	3.81397	0.73299	1.18137
C	5.14317	0.30871	1.22083
C	5.77252	-0.13131	0.05231
C	5.07250	-0.15443	-1.15863
C	3.74855	0.28203	-1.21098
C	-0.07378	2.37279	-2.16114
C	-0.28919	3.23381	-3.23714
C	0.76202	4.01914	-3.72144
C	2.02829	3.94873	-3.12829
C	2.25087	3.09105	-2.05009
H	0.03897	-4.32793	1.43202

H	-0.83941	-4.78661	3.71159
H	-1.72039	-2.93376	5.11612
H	-1.77375	-0.60510	4.24292
H	-0.47694	-4.38518	-0.95246
H	-2.06031	-5.18524	-2.68114
H	-3.32538	-3.55189	-4.07331
H	-3.02353	-1.10107	-3.70930
H	-1.44168	-0.29309	-1.97383
H	2.51751	-2.19109	1.66970
H	4.75706	-3.17953	1.25200
H	5.37297	-3.95628	-1.03297
H	3.75478	-3.72172	-2.91188
H	1.51103	-2.73287	-2.50540
H	-1.16969	2.22251	3.97385
H	0.10836	4.27017	4.57431
H	1.92123	5.10559	3.09112
H	2.51883	3.86224	1.01648
H	3.32331	1.07694	2.09350
H	5.68922	0.32703	2.16528
H	6.81264	-0.45989	0.08482
H	5.55961	-0.51043	-2.06727
H	3.20959	0.27882	-2.15985
H	-0.89195	1.76934	-1.76725
H	-1.28173	3.29379	-3.68428
H	0.59429	4.69212	-4.56398
H	2.84569	4.56409	-3.50739
H	3.24057	3.03678	-1.59281

Sum of electronic and zero-point Energies= -3195.584163
Sum of electronic and thermal Energies= -3195.540383
Sum of electronic and thermal Enthalpies= -3195.539439
Sum of electronic and thermal Free Energies= -3195.662331

(*BP86(D3)/def2-SVP*):

C	0.22636	0.06199	0.17903
P	1.59207	1.12643	-0.02039
P	-0.00692	-1.64940	-0.09259
C	1.37133	2.16840	1.46535
C	2.19201	3.25887	1.80254
C	0.24031	1.84529	2.26284
C	1.89287	4.00859	2.95379
H	3.05636	3.52508	1.17333
C	-0.08419	2.63184	3.38372
C	0.76296	3.69536	3.73682
H	2.53489	4.85803	3.23255
H	-1.00139	2.42674	3.95590
H	0.52793	4.30444	4.62327

C	-0.62264	-2.13289	1.56191
C	-0.96292	-1.03133	2.39367
C	-0.78724	-3.45083	2.02532
C	-1.51866	-1.24840	3.66643
C	-1.30094	-3.66043	3.31722
H	-0.49885	-4.30735	1.39546
C	-1.66643	-2.56673	4.12824
H	-1.83833	-0.39715	4.28610
H	-1.42228	-4.68719	3.69480
H	-2.07810	-2.74691	5.13323
C	3.22471	0.32584	0.01035
C	3.76269	-0.20433	-1.18434
C	3.91668	0.18014	1.23269
C	4.99981	-0.86485	-1.15227
H	3.22018	-0.09590	-2.13637
C	5.15978	-0.47275	1.25053
H	3.49060	0.58729	2.16322
C	5.70150	-0.99098	0.06065
H	5.41840	-1.28253	-2.08031
H	5.71061	-0.57201	2.19859
H	6.67779	-1.49979	0.07862
C	1.52758	2.18527	-1.49752
C	2.68440	2.83217	-1.99110
C	0.26442	2.44613	-2.06855
C	2.56523	3.72756	-3.06622
H	3.67275	2.63359	-1.54713
C	0.15449	3.34592	-3.13950
H	-0.62985	1.94799	-1.66837
C	1.30453	3.98331	-3.63904
H	3.46272	4.22921	-3.45992
H	-0.83818	3.54696	-3.56814
H	1.21928	4.68833	-4.48064
C	1.48343	-2.60400	-0.49205
C	2.42365	-2.85702	0.53251
C	1.69704	-3.08344	-1.80195
C	3.57468	-3.60067	0.24030
H	2.25164	-2.47273	1.55004
C	2.85886	-3.82179	-2.08379
H	0.95256	-2.89285	-2.59110
C	3.79123	-4.08327	-1.06491
H	4.31012	-3.80238	1.03356
H	3.02895	-4.20408	-3.10200
H	4.69560	-4.67074	-1.28736
C	-1.26559	-1.96344	-1.36148
C	-2.13924	-3.06967	-1.27936
C	-1.33855	-1.06891	-2.45160
C	-3.08014	-3.27811	-2.29836
H	-2.10877	-3.74961	-0.41483
C	-2.28747	-1.28606	-3.46200
H	-0.67958	-0.18781	-2.48718
C	-3.15335	-2.39054	-3.38692

H	-3.78004	-4.12440	-2.22837
H	-2.36875	-0.57354	-4.29596
H	-3.91149	-2.54572	-4.16950
P	-0.81161	0.56356	1.52924
C	-2.73025	1.15363	0.97911
C	-2.61651	2.17383	-0.03265
C	-3.54401	0.04031	0.55435
C	-3.16276	1.98007	-1.29925
C	-4.06880	-0.00507	-0.73731
N	-3.89500	0.93447	-1.65797
F	-1.78671	3.21689	0.23379
F	-2.92239	2.91350	-2.24632
F	-4.80411	-1.07484	-1.08406
F	-3.72483	-0.98854	1.41745
F	-2.99728	1.60642	2.33216

Sum of electronic and zero-point Energies= -3192.836320
Sum of electronic and thermal Energies= -3192.792837
Sum of electronic and thermal Enthalpies= -3192.791892
Sum of electronic and thermal Free Energies= -3192.912504

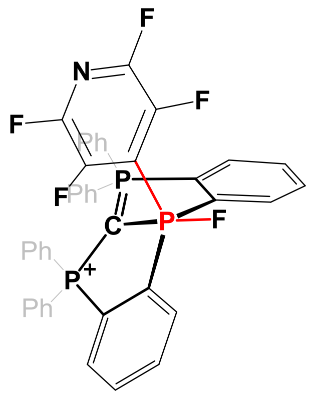

(**4**$^+$)
(*BP86(D3)/def2-TZVP*):

C	-0.25830	0.00741	0.34785
P	-0.00813	-1.56546	-0.26116
P	-1.51851	1.10898	0.06680
C	0.42801	-2.37208	1.29571
C	0.40813	-3.74819	1.53599
C	0.78473	-1.45584	2.30590
C	0.71019	-4.22007	2.81780
H	0.13030	-4.44360	0.74125
C	1.11278	-1.94323	3.57533
C	1.05109	-3.31877	3.83018
H	0.67465	-5.29061	3.02561
H	1.41772	-1.25337	4.35967
H	1.28157	-3.68803	4.83084
C	-1.30503	2.12814	1.53705
C	-0.23705	1.75404	2.37527
C	-2.16830	3.17304	1.87889
C	-0.03965	2.46588	3.56706
C	-1.96038	3.87215	3.07115
H	-3.00623	3.42892	1.22721

C	-0.90002	3.51577	3.90802
H	0.78308	2.20203	4.22715
H	-2.62985	4.68807	3.34767
H	-0.73786	4.05677	4.84168
C	-1.43602	-2.35239	-1.03016
C	-1.52391	-2.45021	-2.42867
C	-2.49822	-2.79035	-0.22142
C	-2.68404	-2.96405	-3.01345
H	-0.68537	-2.13711	-3.05427
C	-3.64909	-3.30749	-0.81350
H	-2.42367	-2.71439	0.86391
C	-3.74507	-3.38830	-2.20773
H	-2.75405	-3.04243	-4.09947
H	-4.47631	-3.64014	-0.18554
H	-4.64875	-3.79203	-2.66731
C	1.39429	-1.67514	-1.40631
C	2.27219	-2.76962	-1.38580
C	1.62435	-0.60101	-2.28351
C	3.37765	-2.78551	-2.23863
H	2.11639	-3.59006	-0.68372
C	2.72488	-0.63026	-3.14088
H	0.96454	0.26902	-2.26498
C	3.60408	-1.71845	-3.11377
H	4.07455	-3.62407	-2.20639
H	2.91540	0.21194	-3.80747
H	4.48013	-1.72508	-3.76388
C	-3.20032	0.41872	0.15212
C	-3.57421	-0.17319	1.37342
C	-4.10819	0.44942	-0.91522
C	-4.84292	-0.72922	1.52041
H	-2.86643	-0.19658	2.20568
C	-5.37270	-0.13013	-0.76764
H	-3.83930	0.92660	-1.85771
C	-5.74174	-0.71437	0.44600
H	-5.13334	-1.17532	2.47308
H	-6.07348	-0.11404	-1.60373
H	-6.73414	-1.15399	0.56007
C	-1.40711	2.08048	-1.46097
C	-1.22918	3.47150	-1.45861
C	-1.42610	1.38007	-2.68218
C	-1.07487	4.15451	-2.66811
H	-1.18398	4.02097	-0.51784
C	-1.27579	2.06910	-3.88575
H	-1.55734	0.29467	-2.69312
C	-1.09949	3.45784	-3.87927
H	-0.92790	5.23557	-2.66126
H	-1.29295	1.52192	-4.82978
H	-0.97638	3.99573	-4.82060
P	0.85178	0.34727	1.82778
C	2.31539	0.77392	0.77039
C	3.41449	-0.08359	0.64079

C	2.34528	1.93246	-0.01196
C	4.43204	0.23586	-0.26585
C	3.41686	2.15960	-0.87667
N	4.43053	1.32726	-1.00144
F	3.49830	-1.22857	1.34466
F	5.46377	-0.60669	-0.40842
F	3.42375	3.26625	-1.63519
F	1.33799	2.83150	0.04925
F	1.86131	0.62702	3.17888

Sum of electronic and zero-point Energies= -3195.646734
Sum of electronic and thermal Energies= -3195.603179
Sum of electronic and thermal Enthalpies= -3195.602235
Sum of electronic and thermal Free Energies= -3195.722850

(*BP86(D3)/def2-SVP*)**:**

C	0.27883	0.02481	0.36613
P	-0.00487	1.56255	-0.34536
P	1.55357	-1.09072	0.13860
C	-0.46049	2.45787	1.17197
C	-0.46011	3.85306	1.33499
C	-0.82160	1.59024	2.23169
C	-0.79405	4.39185	2.59154
H	-0.17815	4.51352	0.49943
C	-1.18590	2.14224	3.47179
C	-1.14755	3.53837	3.65115
H	-0.77497	5.48219	2.74276
H	-1.49890	1.47930	4.28885
H	-1.40678	3.96374	4.63311
C	1.31014	-2.06497	1.65063
C	0.22790	-1.65545	2.46582
C	2.16207	-3.11369	2.03558
C	-0.00352	-2.33688	3.67651
C	1.92274	-3.78203	3.24952
H	3.01308	-3.40118	1.39778
C	0.84444	-3.39236	4.06173
H	-0.84604	-2.03776	4.31223
H	2.58381	-4.60493	3.56222
H	0.65758	-3.91366	5.01335
C	1.42324	2.30994	-1.17751
C	1.50770	2.31062	-2.58724
C	2.50046	2.78090	-0.39484
C	2.68551	2.76005	-3.20854
H	0.65492	1.96747	-3.19457
C	3.66959	3.23003	-1.02515
H	2.42458	2.78501	0.70255
C	3.76535	3.21224	-2.42928
H	2.75615	2.76370	-4.30717
H	4.51316	3.58729	-0.41572
H	4.68658	3.56254	-2.92018
C	-1.43226	1.56446	-1.48386
C	-2.38205	2.60775	-1.47825

C	-1.61366	0.43565	-2.31530
C	-3.51301	2.51810	-2.30643
H	-2.26367	3.46858	-0.80264
C	-2.74011	0.36292	-3.14872
H	-0.89544	-0.39947	-2.27579
C	-3.69076	1.40029	-3.14060
H	-4.26990	3.31672	-2.28529
H	-2.89503	-0.52370	-3.78206
H	-4.58974	1.32351	-3.77084
C	3.23637	-0.37305	0.23684
C	3.53744	0.33520	1.42370
C	4.20020	-0.48380	-0.78485
C	4.79657	0.92864	1.58406
H	2.77512	0.42436	2.21521
C	5.45383	0.13470	-0.62561
H	3.98321	-1.05059	-1.70232
C	5.75331	0.83594	0.55409
H	5.03371	1.46953	2.51336
H	6.20338	0.05751	-1.42819
H	6.74038	1.30843	0.67719
C	1.47109	-2.11794	-1.36763
C	1.22852	-3.50620	-1.32662
C	1.55326	-1.44909	-2.61223
C	1.07753	-4.22096	-2.52730
H	1.13358	-4.02772	-0.36262
C	1.40406	-2.17169	-3.80596
H	1.73283	-0.36150	-2.64914
C	1.16571	-3.55843	-3.76385
H	0.88230	-5.30387	-2.49390
H	1.47154	-1.65018	-4.77335
H	1.04429	-4.12390	-4.70071
P	-0.85528	-0.25282	1.85369
C	-2.30753	-0.73972	0.80290
C	-3.42005	0.10580	0.63865
C	-2.30003	-1.92883	0.05315
C	-4.42217	-0.25929	-0.28438
C	-3.36555	-2.20045	-0.82297
N	-4.38943	-1.38070	-0.97837
F	-3.51516	1.27808	1.28257
F	-5.44784	0.57196	-0.48204
F	-3.34099	-3.32168	-1.54922
F	-1.26960	-2.79187	0.13824
F	-1.87528	-0.46497	3.23702

Sum of electronic and zero-point Energies= -3192.892956
Sum of electronic and thermal Energies= -3192.849531
Sum of electronic and thermal Enthalpies= -3192.848586
Sum of electronic and thermal Free Energies= -3192.968671

$PhSiH_3$
(*BP86(D3)/def2-TZVP*)**:**

C	-2.35380	-0.00017	0.01082

C	-1.65231	-1.21000	0.00301
C	-0.25447	-1.20780	-0.01032
C	0.46927	0.00022	-0.01404
C	-0.25469	1.20789	-0.00992
C	-1.65276	1.20968	0.00342
Si	2.34829	0.00012	0.00597
H	2.87555	-0.05597	1.40543
H	2.86376	-1.19346	-0.73161
H	2.85871	1.24985	-0.63559
H	-3.44571	-0.00038	0.01845
H	-2.19545	-2.15746	0.00353
H	0.27793	-2.16296	-0.02334
H	0.27783	2.16290	-0.02248
H	-2.19611	2.15702	0.00424

Sum of electronic and zero-point Energies= -522.949514
Sum of electronic and thermal Energies= -522.942249
Sum of electronic and thermal Enthalpies= -522.941305
Sum of electronic and thermal Free Energies= -522.982128

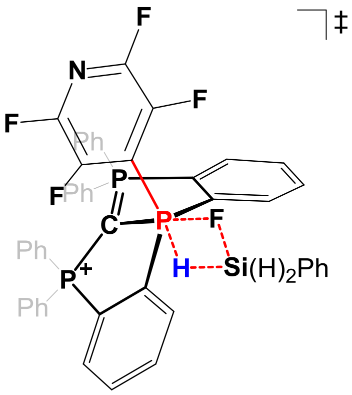

(**TS2**)
(*BP86(D3)/def2-TZVP*):

C	-0.62250	-0.36921	-0.03764
P	-1.01818	0.21544	-1.59946
P	-1.55378	-1.22711	1.11560
C	0.63874	0.18786	-2.29684
C	0.90811	0.19542	-3.67063
C	1.66738	0.14748	-1.33136
C	2.23466	0.14410	-4.10312
H	0.08704	0.22360	-4.39041
C	2.98938	0.12877	-1.78684
C	3.26486	0.11229	-3.15963
H	2.46180	0.12881	-5.17005
H	3.81794	0.13345	-1.08514
H	4.30516	0.07695	-3.48729
C	-0.53502	-0.92481	2.56807
C	0.67432	-0.26083	2.29864

C	-0.90708	-1.26579	3.87463
C	1.48400	0.12376	3.37047
C	-0.08090	-0.88952	4.93496
H	-1.84120	-1.80010	4.06169
C	1.09986	-0.18239	4.68003
H	2.41823	0.65201	3.18386
H	-0.36147	-1.13832	5.95933
H	1.73724	0.12589	5.51057
C	-2.19896	-0.82060	-2.48628
C	-3.36632	-0.28797	-3.05546
C	-1.96001	-2.20603	-2.52776
C	-4.30249	-1.14569	-3.63901
H	-3.55019	0.78663	-3.02961
C	-2.90172	-3.05494	-3.10689
H	-1.04860	-2.61579	-2.09208
C	-4.07694	-2.52557	-3.65560
H	-5.21188	-0.73342	-4.07892
H	-2.71975	-4.13039	-3.12748
H	-4.81549	-3.19210	-4.10436
C	-1.61381	1.93004	-1.60082
C	-0.83275	2.95219	-2.16544
C	-2.78833	2.25209	-0.89564
C	-1.22576	4.28536	-2.02452
H	0.09202	2.71076	-2.69171
C	-3.17514	3.58499	-0.76540
H	-3.38658	1.46842	-0.42853
C	-2.39377	4.60239	-1.32541
H	-0.60875	5.07854	-2.44890
H	-4.07869	3.83148	-0.20615
H	-2.68757	5.64566	-1.20178
C	-1.65686	-3.02036	0.84654
C	-0.55138	-3.66231	0.26318
C	-2.75248	-3.77812	1.29066
C	-0.55105	-5.04759	0.10675
H	0.30728	-3.07382	-0.06676
C	-2.74742	-5.16629	1.12756
H	-3.61051	-3.29027	1.75592
C	-1.65175	-5.80027	0.53397
H	0.31181	-5.54027	-0.34420
H	-3.60267	-5.75308	1.46652
H	-1.65214	-6.88446	0.40929
C	-3.24880	-0.61963	1.28619
C	-3.58272	0.33971	2.25495
C	-4.19418	-1.00214	0.31434

C	-4.85295	0.91995	2.24432
H	-2.84903	0.64383	3.00150
C	-5.45757	-0.40969	0.30736
H	-3.94101	-1.74912	-0.44052
C	-5.78628	0.55266	1.26903
H	-5.11177	1.66502	2.99806
H	-6.18503	-0.70055	-0.45188
H	-6.77547	1.01357	1.26151
C	1.01687	1.98017	0.69446
C	2.02744	2.85151	0.25894
C	-0.10265	2.62520	1.24727
C	1.84882	4.23944	0.34052
C	-0.17815	4.01807	1.29043
N	0.77221	4.80856	0.83892
F	3.19378	2.40614	-0.24127
F	2.82120	5.04545	-0.10840
F	-1.27001	4.59824	1.81541
F	-1.14815	1.93024	1.74816
F	3.02989	-0.00231	1.05350
P	1.04524	0.07666	0.47477
Si	3.17602	-1.91473	0.93462
H	1.62178	-1.49133	0.43306
H	2.75143	-3.23601	0.30961
H	3.04369	-2.09296	2.40188
C	4.90625	-1.76191	0.23048
C	5.34635	-2.72555	-0.69634
C	5.80047	-0.73745	0.60643
C	6.63573	-2.67074	-1.23510
H	4.67068	-3.52801	-1.00375
C	7.08977	-0.68430	0.07477
H	5.48041	0.03039	1.31288
C	7.50848	-1.64959	-0.84939
H	6.95897	-3.42514	-1.95504
H	7.77241	0.11117	0.38001
H	8.51594	-1.60415	-1.26754

Sum of electronic and zero-point Energies= -3718.565465
Sum of electronic and thermal Energies= -3718.514373
Sum of electronic and thermal Enthalpies= -3718.513429
Sum of electronic and thermal Free Energies= -3718.651499

PhSi(F)H$_2$:

C	2.69890	-0.00030	0.18163
C	2.00654	-1.21171	0.08297
C	0.62287	-1.20974	-0.11200

C	-0.09270	0.00025	-0.20964
C	0.62325	1.20995	-0.11136
C	2.00693	1.21138	0.08359
Si	-1.94072	0.00059	-0.44847
H	-2.37207	-1.23451	-1.16446
F	-2.70483	-0.00104	0.98368
H	-2.37188	1.23734	-1.16171
H	3.78048	-0.00051	0.33191
H	2.54649	-2.15796	0.15587
H	0.09397	-2.16344	-0.19273
H	0.09464	2.16385	-0.19158
H	2.54718	2.15742	0.15697

Sum of electronic and zero-point Energies= -622.309742
Sum of electronic and thermal Energies= -622.301941
Sum of electronic and thermal Enthalpies= -622.300997
Sum of electronic and thermal Free Energies= -622.343697

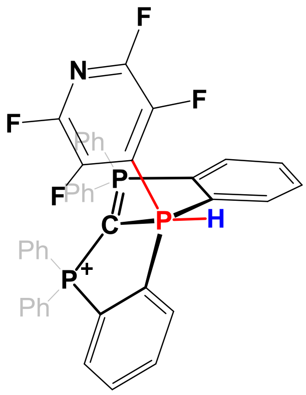

(**6**$^+$)
(*BP86(D3)/def2-TZVP*):

C	-0.45038	-3.65521	1.75958
C	-0.83539	-4.05445	3.04393
C	-1.25265	-3.10392	3.98211
C	-1.29700	-1.74851	3.63967
C	-0.88682	-1.34104	2.36859
C	-0.46156	-2.29563	1.43470
P	0.04413	-1.57856	-0.15381
C	-1.22451	-2.02471	-1.37869
C	0.11819	0.06827	0.28914
C	1.57082	-2.38777	-0.68922
C	-1.42951	-3.37428	-1.71937
C	-2.45306	-3.72225	-2.60033
C	-3.27779	-2.73091	-3.14711
C	-3.07168	-1.38872	-2.81874
C	-2.04670	-1.03722	-1.93700
C	2.52232	-2.77014	0.27180
C	3.71235	-3.37167	-0.13447
C	3.96139	-3.58814	-1.49458
C	3.02235	-3.19642	-2.45282

C	1.82576	-2.59565	-2.05473
P	-0.96363	0.45091	1.87523
C	0.12791	1.88651	2.30140
C	-2.45069	0.88678	0.84677
P	1.38932	1.16538	0.00558
C	-2.51446	2.06090	0.08558
C	-3.59689	2.27177	-0.77135
N	-4.59559	1.41933	-0.89744
C	-4.57583	0.32649	-0.16332
C	-3.54194	0.01921	0.72918
C	-0.12424	2.64442	3.44764
C	0.67407	3.75603	3.73597
C	1.71646	4.11523	2.87510
C	1.96989	3.36302	1.72314
C	1.17602	2.24698	1.44530
F	-1.52820	2.97991	0.14278
F	-3.63091	3.38195	-1.52242
F	-3.59298	-1.13583	1.42152
F	-5.58924	-0.53546	-0.30779
C	1.31049	2.16926	-1.50541
C	3.08891	0.52233	0.07763
C	3.68818	0.31142	1.33122
C	4.97734	-0.21759	1.40763
C	5.66693	-0.55241	0.23745
C	5.06401	-0.36476	-1.00992
C	3.77914	0.17422	-1.09446
C	0.14867	2.14407	-2.28812
C	0.04344	2.95931	-3.41628
C	1.09925	3.80680	-3.76453
C	2.26422	3.83420	-2.98852
C	2.37497	3.01705	-1.86316
H	-0.12129	-4.39864	1.03142
H	-0.80911	-5.11167	3.31280
H	-1.55103	-3.42073	4.98273
H	-1.65398	-1.00607	4.35769
H	-0.78528	-4.15267	-1.30607
H	-2.61111	-4.77008	-2.86037
H	-4.08233	-3.00807	-3.83011
H	-3.71470	-0.61258	-3.23685
H	-1.89026	0.00639	-1.66570
H	2.33097	-2.59731	1.33204
H	4.45035	-3.66815	0.61188
H	4.89271	-4.06322	-1.80743
H	3.21695	-3.36404	-3.51329

H	1.08663	-2.30268	-2.80243
H	-0.94758	2.36581	4.11004
H	0.48013	4.34592	4.63319
H	2.33510	4.98559	3.09970
H	2.78287	3.64783	1.05307
H	3.15103	0.57306	2.24513
H	5.44673	-0.36285	2.38203
H	6.67625	-0.96302	0.29807
H	5.59470	-0.63978	-1.92249
H	3.31616	0.32494	-2.07066
H	-0.67069	1.48502	-2.00258
H	-0.86470	2.93579	-4.02042
H	1.01725	4.44633	-4.64495
H	3.09023	4.49178	-3.26412
H	3.29507	3.02678	-1.27580
H	-1.73916	0.71284	3.09522

Sum of electronic and zero-point Energies= -3096.295683
Sum of electronic and thermal Energies= -3096.252895
Sum of electronic and thermal Enthalpies= -3096.251951
Sum of electronic and thermal Free Energies= -3096.372070

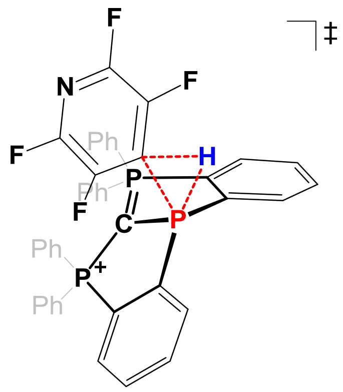

(**TS3**)

(*BP86(D3)/def2-TZVP*):

C	0.09677	-3.58641	1.95311
C	-0.45621	-3.94533	3.18821
C	-1.18698	-3.01476	3.93415
C	-1.37140	-1.71040	3.46203
C	-0.81042	-1.34368	2.23682
C	-0.07014	-2.28236	1.48528
P	0.49776	-1.65505	-0.12594
C	-0.59234	-2.34508	-1.39222
C	0.26943	0.02836	0.15689
C	2.18395	-2.19511	-0.45059
C	-0.55029	-3.71930	-1.69289
C	-1.48995	-4.26248	-2.56806
C	-2.47596	-3.44510	-3.13596

C	-2.51581	-2.08015	-2.84120
C	-1.57295	-1.52700	-1.97236
C	3.10389	-2.24656	0.61157
C	4.41967	-2.63332	0.36416
C	4.82175	-2.96442	-0.93530
C	3.91080	-2.90112	-1.99367
C	2.58984	-2.51471	-1.75705
P	-0.98259	0.26625	1.42001
C	-0.36116	1.82391	2.10014
C	-2.68292	0.47094	0.87782
P	1.26794	1.40919	-0.05648
C	-3.07346	1.68088	0.19782
C	-4.19515	1.71903	-0.61095
N	-4.96442	0.68060	-0.89636
C	-4.58779	-0.47171	-0.35769
C	-3.49615	-0.64808	0.46823
C	-0.90012	2.51498	3.18922
C	-0.36016	3.75326	3.54934
C	0.70802	4.30158	2.83027
C	1.24660	3.61738	1.73565
C	0.72050	2.37281	1.38132
F	-2.32117	2.80918	0.37163
F	-4.51277	2.89092	-1.19900
F	-3.18163	-1.89309	0.92295
F	-5.31194	-1.55943	-0.69182
C	0.94531	2.38358	-1.54381
C	3.04798	1.11740	0.05328
C	3.68846	1.16787	1.30300
C	5.05284	0.88699	1.39372
C	5.77445	0.53962	0.24740
C	5.13268	0.46636	-0.99343
C	3.77239	0.75716	-1.09656
C	-0.33349	2.30447	-2.11887
C	-0.66505	3.12717	-3.19500
C	0.27652	4.03108	-3.69852
C	1.54906	4.11832	-3.12254
C	1.88743	3.30181	-2.04203
H	0.66007	-4.31640	1.36886
H	-0.31318	-4.95738	3.56985
H	-1.61521	-3.30465	4.89473
H	-1.95127	-0.98300	4.03184
H	0.21241	-4.36212	-1.24795
H	-1.46065	-5.32781	-2.80154
H	-3.21849	-3.87791	-3.80820

H	-3.29057	-1.44481	-3.27234
H	-1.61546	-0.46657	-1.72270
H	2.78818	-1.98601	1.62339
H	5.13474	-2.67508	1.18662
H	5.85173	-3.27276	-1.12228
H	4.22594	-3.15869	-3.00594
H	1.87420	-2.47327	-2.57996
H	-1.74374	2.08823	3.73473
H	-0.77868	4.29552	4.39842
H	1.12212	5.26830	3.12055
H	2.06760	4.05274	1.16228
H	3.12464	1.43654	2.19815
H	5.55396	0.94381	2.36131
H	6.84131	0.32228	0.32111
H	5.69291	0.18151	-1.88487
H	3.27605	0.70775	-2.06724
H	-1.07188	1.61210	-1.71120
H	-1.66214	3.06799	-3.63336
H	0.01687	4.67390	-4.54125
H	2.28053	4.82632	-3.51528
H	2.88015	3.37624	-1.59420
H	-2.63308	0.56767	2.40874

Sum of electronic and zero-point Energies= -3096.250410
Sum of electronic and thermal Energies= -3096.207521
Sum of electronic and thermal Enthalpies= -3096.206577
Sum of electronic and thermal Free Energies= -3096.327609

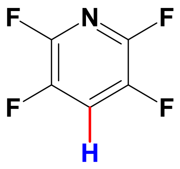

(**7**)

(*BP86(D3)/def2-TZVP*):

N	0.00000	0.00000	1.37068
C	0.00000	1.13515	0.70193
C	0.00000	1.19307	-0.69704
C	0.00000	0.00000	-1.41406
C	0.00000	-1.19307	-0.69704
C	0.00000	-1.13515	0.70193
F	0.00000	-2.37737	-1.33673
F	0.00000	-2.27434	1.41088
F	0.00000	2.27434	1.41088
F	0.00000	2.37737	-1.33673
H	0.00000	0.00000	-2.50360

Sum of electronic and zero-point Energies= -645.457844

Sum of electronic and thermal Energies= -645.450196
Sum of electronic and thermal Enthalpies= -645.449252
Sum of electronic and thermal Free Energies= -645.489935

$Me_3SiNMe2$
(*BP86(D3)/def2-SVP*):

Si	-0.61666	0.00001	-0.00456
C	-1.33066	1.54702	-0.82916
H	-2.43974	1.48580	-0.83568
H	-1.05679	2.47969	-0.29308
H	-0.98453	1.63538	-1.88013
C	-1.10082	-0.00016	1.83261
H	-2.20390	-0.00018	1.96845
H	-0.69582	-0.89713	2.34804
H	-0.69583	0.89670	2.34822
C	-1.33070	-1.54683	-0.82944
H	-0.98458	-1.63501	-1.88043
H	-1.05686	-2.47961	-0.29353
H	-2.43979	-1.48559	-0.83595
N	1.13775	0.00000	-0.23648
C	1.91360	-1.20980	-0.01589
H	1.31698	-2.11531	-0.24865
H	2.81258	-1.23210	-0.67821
H	2.28856	-1.31510	1.03675
C	1.91365	1.20976	-0.01587
H	2.81264	1.23204	-0.67819
H	1.31707	2.11530	-0.24861
H	2.28861	1.31503	1.03678

Sum of electronic and zero-point Energies= -543.419276
Sum of electronic and thermal Energies= -543.407108
Sum of electronic and thermal Enthalpies= -543.406164
Sum of electronic and thermal Free Energies= -543.457072

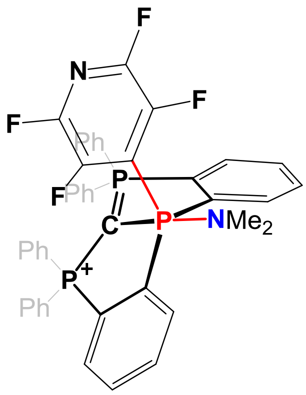

(6-N$^+$)

(*BP86(D3)/def2-SVP*):

C	0.29110	0.12284	-0.17980

P	0.19254	-1.51734	-0.64634
P	1.45666	0.95990	0.73251
C	-0.23916	-1.24340	-2.40473
C	-0.00945	-2.16308	-3.44088
C	-0.65094	0.08511	-2.68829
C	-0.13806	-1.74264	-4.77894
H	0.31788	-3.18953	-3.21082
C	-0.75528	0.49889	-4.02701
C	-0.48426	-0.41230	-5.06705
H	0.06074	-2.45200	-5.59705
H	-1.04160	1.53630	-4.25512
H	-0.54669	-0.07470	-6.11312
C	1.22425	2.60987	-0.00365
C	0.26057	2.67364	-1.03631
C	2.12685	3.66527	0.21993
C	0.27257	3.78818	-1.89936
C	2.09476	4.79240	-0.61849
H	2.88406	3.58602	1.01625
C	1.18984	4.83450	-1.69341
H	-0.45124	3.82943	-2.72537
H	2.79951	5.62164	-0.45261
H	1.19274	5.69358	-2.38215
C	1.74716	-2.45120	-0.50446
C	1.95223	-3.29851	0.60732
C	2.79377	-2.21449	-1.42250
C	3.20944	-3.89448	0.80315
H	1.12852	-3.49990	1.31073
C	4.04053	-2.82445	-1.22434
H	2.63452	-1.54822	-2.28364
C	4.25167	-3.65664	-0.10969
H	3.37166	-4.55477	1.66904
H	4.85745	-2.63682	-1.93713
H	5.23526	-4.12655	0.04520
C	-1.09363	-2.48884	0.21349
C	-1.79430	-3.52777	-0.43269
C	-1.39476	-2.14615	1.54981
C	-2.80321	-4.21581	0.26008
H	-1.58026	-3.77742	-1.48361
C	-2.39484	-2.84897	2.23981
H	-0.88098	-1.29572	2.02592
C	-3.10226	-3.87804	1.59266
H	-3.37018	-5.01124	-0.24741
H	-2.64746	-2.56789	3.27339
H	-3.90663	-4.40860	2.12482

C	3.20994	0.54909	0.36926
C	3.66140	0.82842	-0.94248
C	4.08841	-0.02620	1.30858
C	4.98658	0.54926	-1.30166
H	2.96923	1.26868	-1.67866
C	5.41002	-0.32838	0.93135
H	3.75519	-0.23136	2.33655
C	5.86169	-0.03437	-0.36539
H	5.33957	0.78368	-2.31800
H	6.09228	-0.78521	1.66467
H	6.90172	-0.25828	-0.64967
C	1.24702	0.93433	2.54494
C	0.95255	2.09784	3.28429
C	1.28759	-0.32471	3.18959
C	0.70346	2.00004	4.66399
H	0.89209	3.07524	2.78207
C	1.04437	-0.41245	4.56893
H	1.50251	-1.23915	2.61064
C	0.75000	0.75028	5.30590
H	0.46366	2.90736	5.23950
H	1.07806	-1.39216	5.07033
H	0.55044	0.67964	6.38636
P	-0.94644	1.22869	-1.23850
C	-2.28476	0.67506	-0.03280
C	-3.24399	-0.30600	-0.32965
C	-2.38329	1.29210	1.22391
C	-4.20764	-0.63738	0.64292
C	-3.38117	0.88248	2.12321
N	-4.26385	-0.05810	1.82771
F	-3.21449	-0.98145	-1.49788
F	-5.09453	-1.59977	0.37322
F	-3.44928	1.45019	3.33105
F	-1.49776	2.25541	1.56915
N	-2.18310	2.31037	-2.06932
C	-3.33029	1.71683	-2.76360
H	-4.17451	1.47750	-2.06797
H	-3.72377	2.44063	-3.51152
H	-3.07281	0.78328	-3.29283
C	-2.67785	3.42838	-1.25172
H	-3.02382	4.25685	-1.90855
H	-3.54529	3.12810	-0.61162
H	-1.90187	3.83624	-0.57746

Sum of electronic and zero-point Energies= -3227.480441
Sum of electronic and thermal Energies= -3227.433654

Sum of electronic and thermal Enthalpies= -3227.432710
Sum of electronic and thermal Free Energies= -3227.559330

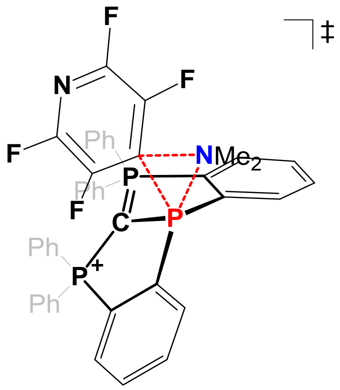

(**TS3-N**)

(*BP86(D3)/def2-SVP*):

C	0.40697	-0.05986	0.23320
P	0.81489	1.61260	0.26445
P	1.12534	-1.47751	-0.43008
C	0.27908	1.95178	1.98736
C	0.66140	3.06358	2.75245
C	-0.52393	0.92657	2.55906
C	0.26911	3.13838	4.10350
H	1.28932	3.85500	2.31368
C	-0.91048	1.00720	3.90501
C	-0.49893	2.10989	4.67745
H	0.57852	4.00057	4.71395
H	-1.52486	0.20850	4.34553
H	-0.78412	2.16577	5.73924
C	0.53568	-2.67099	0.82400
C	-0.36295	-2.13040	1.78060
C	1.02263	-3.98096	0.96204
C	-0.73155	-2.89160	2.90049
C	0.63563	-4.74420	2.08000
H	1.72741	-4.39427	0.22321
C	-0.21767	-4.19263	3.05294
H	-1.41830	-2.46429	3.64543
H	1.01906	-5.76895	2.20039
H	-0.49252	-4.78418	3.93977
C	2.58397	1.95782	0.05158
C	3.06098	2.46343	-1.17796
C	3.48591	1.59487	1.07571
C	4.44413	2.60580	-1.37692
H	2.35311	2.75082	-1.97121
C	4.86375	1.74934	0.86894
H	3.10671	1.18722	2.02596
C	5.34261	2.25056	-0.35600
H	4.82032	3.00080	-2.33315
H	5.57004	1.46777	1.66451
H	6.42614	2.36569	-0.51416
C	-0.09684	2.64344	-0.92397
C	-0.01238	4.05297	-0.87087

C	-0.89379	2.00835	-1.89924
C	-0.73715	4.82198	-1.79313
H	0.61449	4.55129	-0.11389
C	-1.61413	2.78801	-2.81987
H	-0.97562	0.91053	-1.91459
C	-1.53846	4.18924	-2.76411
H	-0.68420	5.92076	-1.75108
H	-2.26226	2.29049	-3.55667
H	-2.11647	4.79760	-3.47683
C	2.94940	-1.50488	-0.38420
C	3.57324	-1.69236	0.87153
C	3.73087	-1.30029	-1.54033
C	4.97198	-1.69447	0.96099
H	2.96250	-1.84461	1.77547
C	5.13241	-1.27677	-1.43496
H	3.25369	-1.17505	-2.52396
C	5.75221	-1.48083	-0.19059
H	5.45702	-1.85919	1.93561
H	5.74198	-1.11127	-2.33642
H	6.85082	-1.47703	-0.11703
C	0.53552	-1.88879	-2.10141
C	-0.14848	-3.09098	-2.37473
C	0.63332	-0.88275	-3.09198
C	-0.74488	-3.27667	-3.63270
H	-0.25803	-3.86016	-1.59657
C	0.04890	-1.08532	-4.35055
H	1.13766	0.07296	-2.87155
C	-0.64897	-2.27849	-4.61666
H	-1.30511	-4.20148	-3.83705
H	0.11931	-0.30047	-5.11926
H	-1.13108	-2.42490	-5.59540
P	-0.94397	-0.43213	1.40133
C	-2.52336	-0.26407	0.46058
C	-3.00160	1.04614	0.09720
C	-2.77560	-1.22627	-0.58238
C	-3.77280	1.23904	-1.04674
C	-3.52043	-0.89923	-1.71305
N	-4.04040	0.29989	-1.94737
F	-2.62515	2.11839	0.85604
F	-4.21835	2.47973	-1.30886
F	-3.70253	-1.84556	-2.65152
F	-2.22462	-2.47563	-0.46461
N	-2.98176	-0.80173	2.23651
C	-3.82272	0.22212	2.85033
H	-4.76752	0.36440	2.27344
H	-4.10398	-0.06425	3.89010
H	-3.30799	1.19938	2.88087
C	-3.67662	-2.08475	2.17787
H	-3.95439	-2.43745	3.19821
H	-4.61778	-2.00257	1.58340
H	-3.05275	-2.86105	1.69802

Sum of electronic and zero-point Energies= -3227.454835
Sum of electronic and thermal Energies= -3227.408297
Sum of electronic and thermal Enthalpies= -3227.407353
Sum of electronic and thermal Free Energies= -3227.534104

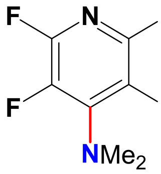

(**7-N**)

(*BP86(D3)/def2-SVP*):

C	0.61464	0.00003	0.00010
C	-0.15922	-1.19896	-0.05933
C	-0.15942	1.19901	0.05906
C	-1.55913	-1.13036	-0.03887
C	-1.55923	1.13032	0.03878
N	-2.24525	-0.00010	0.00002
F	0.44914	-2.39994	-0.17324
F	-2.25375	-2.27595	-0.09556
F	-2.25408	2.27575	0.09579
F	0.44903	2.39996	0.17285
N	1.99124	0.00028	0.00044
C	2.77188	1.08619	-0.58380
H	3.19693	1.76772	0.18761
H	3.61435	0.65168	-1.16531
H	2.15713	1.68668	-1.27717
C	2.77185	-1.08613	0.58386
H	3.19610	-1.76761	-0.18805
H	3.61487	-0.65207	1.16488
H	2.15733	-1.68656	1.27748

Sum of electronic and zero-point Energies= -778.509758
Sum of electronic and thermal Energies= -778.497431
Sum of electronic and thermal Enthalpies= -778.496487
Sum of electronic and thermal Free Energies= -778.548349

Me_3SiF

(*BP86(D3)/def2-SVP*):

Si	0.00001	0.00033	0.02383
C	1.44911	1.06192	-0.53161
H	1.50352	1.10548	-1.64045
H	1.35043	2.10116	-0.15396
H	2.41073	0.65104	-0.15895
C	-1.64395	0.72305	-0.53336
H	-1.70537	0.75469	-1.64224
H	-2.49380	0.11283	-0.16215
H	-1.77392	1.75861	-0.15501
C	0.19612	-1.78523	-0.53226
H	1.14750	-2.21752	-0.15750
H	-0.63809	-2.41411	-0.15676

H	0.20337	-1.85461	-1.64113
F	-0.00135	-0.00006	1.67978

Sum of electronic and zero-point Energies= -508.828938
Sum of electronic and thermal Energies= -508.820371
Sum of electronic and thermal Enthalpies= -508.819427
Sum of electronic and thermal Free Energies= -508.860916

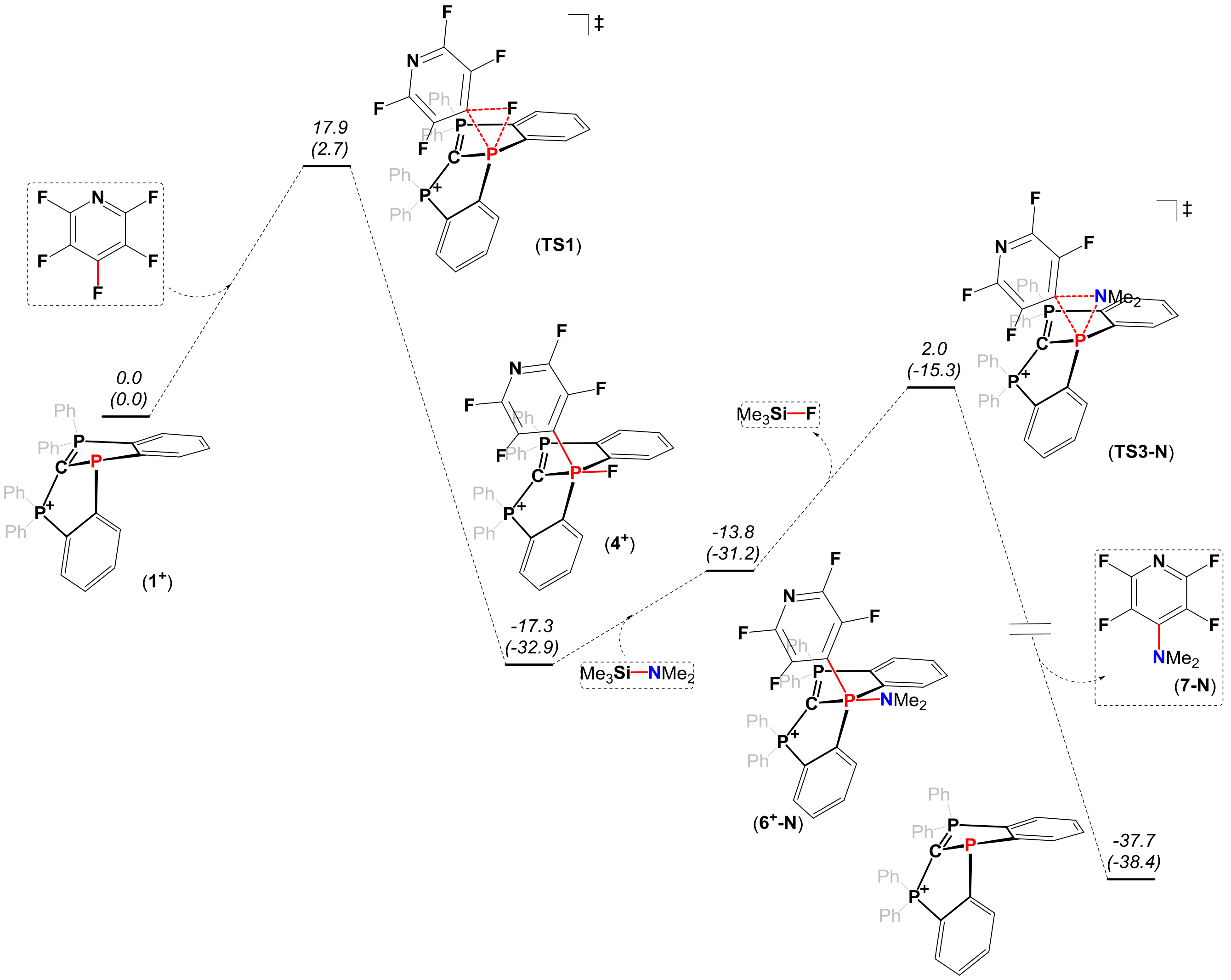

Figure S61. DFT calculated (BP86-D3/def2SVP)[15,16] potential energy surface of the proposed mechanism of **1⁺** catalyzed C−N bond cross-coupling reaction. Free Gibbs energies (enthalpies) are given relative to the starting materials.

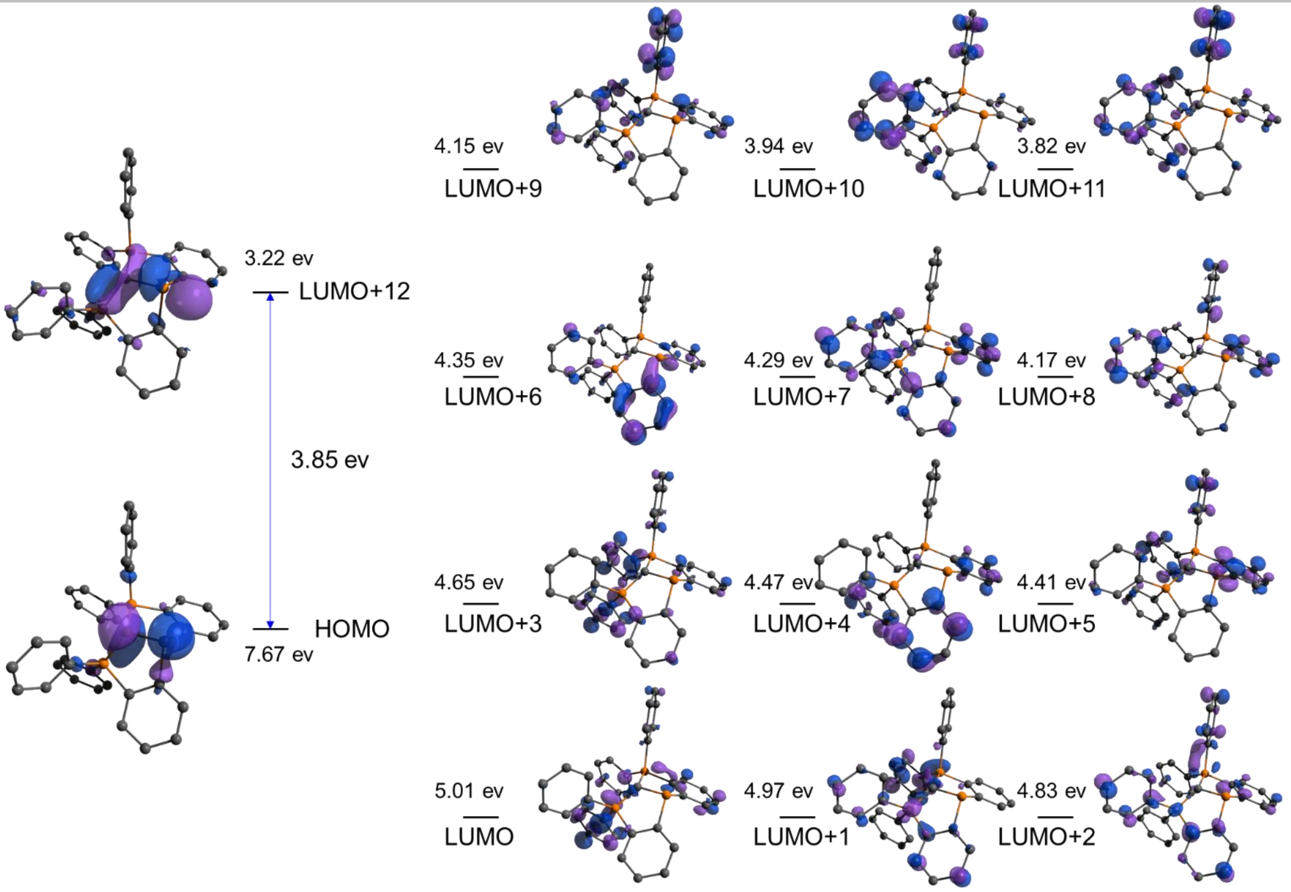

Figure S62. Frontier orbitals of **1**$^{+}$.

5. References

(1) S. C. Böttger, C. Poggel C. and J. Sundermeyer, *Organometallics* **39**, 3789–3793 (2020).
(2) Bruker (2012). Apex-II. Bruker AXS Inc., Madison, Wisconsin, USA.
(3) Bruker (2013). SAINT v8.34A. Bruker AXS Inc., Madison, Wisconsin, USA.
(4) Bruker (2014/5). Sadabs, 2014/5. Bruker AXS Inc., Madison, Wisconsin, USA.
(5) O. V. Dolomanov, L. J. Bourhis, R. J. Gildea, J. A. K. Howard, H. *J.* Puschmann*. Appl. Crystallogr*. **2009**, *42*, 339-341.
(6) G. M. Sheldrick, *Acta Cryst. A*, **2015**, *71*, 3-8.
(7) S. C. Böttger, C. Poggel C. and J. Sundermeyer. *Organometallics* **39**, 3789–3793 (2020).
(8) A. V. Gutov, E. B. Rusanov, A. B. Ryabitskii & A. N. Chernega. Octafluoro-4,4′-bipyridine and its derivatives: Synthesis, molecular and crystal structure. *J. Fluor. Chem.* **131**, 278–281 (2010).
(9) Y. Pang, M. Leutzsch, N. Nöthling, F. Katzenburg & Josep Cornella. Catalytic Hydrodefluorination via Oxidative Addition, Ligand Metathesis, and Reductive Elimination at Bi(I)/Bi(III) Centers. J. Am. Chem. Soc. 2021, 143, 12487−12493
(10) H. Yoshida, T. Minabe, J. Ohshita & A. Kunai. Aminosilylation of arynes with aminosilanes: synthesis of 2-silylaniline derivatives. *Chem. Commun.* 3454 (2005).
(11) R. D. Chambers, G. Sandford & J. Trmcic. Continuous flow glassware reactors for the laboratory. *J. Fluor. Chem.* **128**, 1439–1443 (2007).
(12) N. Matsuda, K. Hirano, T. Satoh, & M. Miura. Copper-Catalyzed Direct Amination of Electron-Deficient Arenes with Hydroxylamines. *Org. Lett.* **13**, 2860–2863 (2011).

(13) L. J. Bole, L. Davin, A. R. Kennedy, R. McLellan & E. Hevia. Magnesium-mediated arylation of amines via C–F bond activation of fluoroarenes. *Chem. Commun.* **55**, 4339–4342 (2019).
(14) Gaussian 09, Revision D.01, M. J. Frisch, G. W. Trucks, H. B. Schlegel, G. E. Scuseria, M. A. Robb, J. R. Cheeseman, G. Scalmani, V. Barone, B. Mennucci, G. A. Petersson, H. Nakatsuji, M. Caricato, X. Li, H. P. Hratchian, A. F. Izmaylov, J. Bloino, G. Zheng, J. L. Sonnengerg, M. Hada, M. Ehara, K. Toyota, R. Fukuda, J. Hasegawa, M. Ishida, T. Nakajima, Y. Honda, O. Kitao, H. Nakai, T. Vreven, J. A. Montgomery Jr, J. E. Peralta, F. Ogliaro, M. Bearpark, J. J. Heyd, E. Brothers, K. N. Kudin, V. N. Staroverov, T. Keith, R. Kobayashi, J. Normand, K. Raghavachari, A. Rendell, J. C. Burant, S. S. Iyengar, J. Tomasi, M. Cossi, N. Rega, N. J. Millam, M. Klene, J. E. Knox, J. B. Cross, V. Bakken, C. Adamo, J. Jaramillo, R. Gomperts, R. E. Stratmann, O. Yazyev, A. J. Austin, R. Cammi, C. Pomelli, J. W. Ochterski, R. L. Martin, K. Morokuma, V. G. Zakrzewski, G. A. Voth, P. Salvador, J. J. Dannenberg, S. Dapprich, A. D. Daniels, Ö. Farkas, J. B. Foresman, J. V. Ortiz, J. Cioslowski and D. J. Fox, Gaussian, Inc., Wallingford CT, 2010.
(15) a) A. D. Becke, *Phys. Rev. A: At., Mol. Opt. Phys*. **1988**, *38*, 3098-3100. b) J. P. Perdew, *Phys. Rev. B: Condens. Matter Mater. Phys*. **1986**, *34*, 7406.
(16) F. Weigend, R. Ahlrichs, *Phys. Chem. Chem. Phys.* **2005**, 7, 3297-3305.
